# A critical assessment of *Mus musculus *gene function prediction using integrated genomic evidence

**DOI:** 10.1186/gb-2008-9-s1-s2

**Published:** 2008-06-27

**Authors:** Lourdes Peña-Castillo, Murat Tasan, Chad L Myers, Hyunju Lee, Trupti Joshi, Chao Zhang, Yuanfang Guan, Michele Leone, Andrea Pagnani, Wan Kyu Kim, Chase Krumpelman, Weidong Tian, Guillaume Obozinski, Yanjun Qi, Sara Mostafavi, Guan Ning Lin, Gabriel F Berriz, Francis D Gibbons, Gert Lanckriet, Jian Qiu, Charles Grant, Zafer Barutcuoglu, David P Hill, David Warde-Farley, Chris Grouios, Debajyoti Ray, Judith A Blake, Minghua Deng, Michael I Jordan, William S Noble, Quaid Morris, Judith Klein-Seetharaman, Ziv Bar-Joseph, Ting Chen, Fengzhu Sun, Olga G Troyanskaya, Edward M Marcotte, Dong Xu, Timothy R Hughes, Frederick P Roth

**Affiliations:** 1 Donnelly Centre for Cellular and Biomolecular Research, University of Toronto, Toronto, ON M5S3E1, Canada; 2 Department of Biological Chemistry and Molecular Pharmacology, Harvard Medical School, Boston, MA 02115, USA; 3 Lewis-Sigler Institute for Integrative Genomics and Department of Molecular Biology, Princeton University, Princeton, NJ 08544, USA; 4 Department of Information and Communications, Gwangju Institute of Science and Technology, Gwangju, 500-712 Republic of Korea; 5 Digital Biology Laboratory, Computer Science Department and Christopher S Bond Life Sciences Center, University of Missouri, Columbia, MO 65211, USA; 6 ISI Foundation, Torino, 10133, Italy; 7 Center for Systems and Synthetic Biology, Institute for Cellular and Molecular Biology, University of Texas at Austin, Austin, TX 78712, USA; 8 Department of Electrical and Computer Engineering, Institute for Cellular and Molecular Biology, University of Texas at Austin, Austin, TX 78712, USA; 9 Department of Statistics, UC Berkeley, Berkeley, CA 94720-3860, USA; 10 School of Computer Science, Carnegie Mellon University, Pittsburgh, PA 15213, USA; 11 Department of Computer Science, University of Toronto, Toronto, ON M5S3G4, Canada; 12 Department of Electrical and Computer Engineering, UC San Diego, La Jolla, CA 92093-0407, USA; 13 Department of Genome Sciences, University of Washington, Seattle, WA 98195-5065, USA; 14 Department of Computer Science, Princeton University, Princeton, NJ 08544, USA; 15 Bioinformatics and Computational Biology, The Jackson Laboratory, Bar Harbor, ME 04609, USA; 16 Gatsby Computational Neuroscience Unit, London, WC1N 3AR, UK; 17 School of Mathematical Sciences and Center for Theoretical Biology, Peking University, Beijing 100871, PRC; 18 Department of Electrical Engineering and Computer Science, and Department of Statistics, UC Berkeley, Berkeley, CA 94720-1776, USA; 19 Department of Genome Sciences, and Department of Computer Science and Engineering, University of Washington, Seattle, WA 98195, USA; 20 Banting and Best Department of Medical Research, University of Toronto, Toronto, ON M5S 3E1, Canada; 21 Department of Structural Biology, University of Pittsburgh School of Medicine, Pittsburgh, PA 15260, USA; 22 Molecular and Computational Biology Program, Department of Biological Sciences, University of Southern California, Los Angeles, CA 90089, USA; 23 Center for Cancer Systems Biology, Dana-Farber Cancer Institute, Boston, MA 02115, USA

## Abstract

**Background:**

Several years after sequencing the human genome and the mouse genome, much remains to be discovered about the functions of most human and mouse genes. Computational prediction of gene function promises to help focus limited experimental resources on the most likely hypotheses. Several algorithms using diverse genomic data have been applied to this task in model organisms; however, the performance of such approaches in mammals has not yet been evaluated.

**Results:**

In this study, a standardized collection of mouse functional genomic data was assembled; nine bioinformatics teams used this data set to independently train classifiers and generate predictions of function, as defined by Gene Ontology (GO) terms, for 21,603 mouse genes; and the best performing submissions were combined in a single set of predictions. We identified strengths and weaknesses of current functional genomic data sets and compared the performance of function prediction algorithms. This analysis inferred functions for 76% of mouse genes, including 5,000 currently uncharacterized genes. At a recall rate of 20%, a unified set of predictions averaged 41% precision, with 26% of GO terms achieving a precision better than 90%.

**Conclusion:**

We performed a systematic evaluation of diverse, independently developed computational approaches for predicting gene function from heterogeneous data sources in mammals. The results show that currently available data for mammals allows predictions with both breadth and accuracy. Importantly, many highly novel predictions emerge for the 38% of mouse genes that remain uncharacterized.

## Background

Determination of gene function is a central goal of modern biology, and is a starting point for detailed mechanistic studies. Computational approaches can provide predictions of gene function based on the integration of heterogeneous data sources [1-10]. These predictions can serve as a principled method of 'triage', focusing experimental resources on the hypotheses (predictions) that are more likely to be true. Moreover, predictions that are associated with measures of confidence allow experimental biologists to adjust the number of predictions they are willing to consider based on the trade-off between false positive rate, the importance of the biological question, and the cost of follow-up experiments. For example, mouse researchers have been faced for years with the problem of deciding which genes to mutate in reverse-genetic studies, and the problem of deciding which physiological and molecular phenotypes to assay for each mutant strain. Today, there are thousands of Gene Trap alleles [11], and within a few years investigators will have access to a virtually complete collection of engineered knockouts [12]. Issues of both expense and ethics that are associated with model organism experiments motivate the thoughtful justification of planned experiments.

Several algorithms have been applied to heterogeneous data sources to predict gene function [1-10,13], with the integration of these sources clearly improving prediction performance [14,15]. However, these studies have been primarily focused on the yeast *Saccharomyces cerevisiae *and other non-mammalian model organisms [16-18], and it has not been clear how well such algorithms will scale to the large genomes and networks of mammals, despite the basic genetic, biochemical and cellular organizational principles that are shared across the eukaryotic kingdom [19-21]. Moreover, it is unclear whether accurate function predictions can be made given the amount and quality of genomic and function annotation data available for mammals. (Although genes with even a single annotation are often referred to as genes of 'known function', only a minority has been exhaustively studied. Therefore, most 'known function' genes are still incompletely annotated.) Although comparisons using standardized data sets and performance criteria are the best way to assess the strengths and weaknesses of the algorithms employed [22-24], our ability to predict gene function using integrated genomic data has not been systematically compared in this way across multiple bioinformatics groups in any organism.

We assembled a large collection of *Mus musculus *data, independently developed nine different computational methods using these data to predict gene functions, and compared the predictive performance of each submission using held-out genes, a prospective evaluation, and a focused literature-based assessment of the top novel predictions. We have provided confidence scores and estimates of prediction accuracy (precision) at different levels of sensitivity (recall), and combined the best submissions in a single set of predictions. We report thousands of predicted functions for mouse genes that are supported by multiple data types and algorithms, and share the results via a web resource that facilitates searching and browsing in the context of the underlying supporting evidence.

This community effort has suggested new function assignments or refinements of previous annotations for the majority of mouse genes. Based on a prospective evaluation of entirely novel predictions, including many for uncharacterized (without any function annotations) genes, we expect that predictions provided here will productively guide further experimentation towards more likely hypotheses.

## Results

### Organization of a community function prediction comparison

The overall structure of our study was to provide groups of investigators (participants) with a collection of data sets in which the gene identifiers were standardized and associated with known functional annotations. The participants then used their algorithms to assign a score reflecting confidence in whether each gene had each function. To enable evaluation of the results, and to calibrate confidence scores for novel predictions within each category, a subset of genes with known functions was 'held out' (that is, function annotations were not given to the participants).

We therefore began by assembling an extensive collection of *M. musculus *data, including gene expression across multiple tissues, protein sequence pattern annotations, protein-protein interactions, phenotype annotations, disease associations (of human orthologs), gene function annotations, and phylogenetic profiles from a variety of publicly available sources. (Table 1 summarizes the data sources; for a full description of the data see the references cited in Table 1.) These data sets were chosen because they encompass many genes, and have been shown to contain information reflecting gene function [7,21,25-27]. Protein interaction data include 'interologs' transferred from other organisms via orthology [28,29]. To avoid circularity, the data collection did not directly include protein or DNA sequences, since homology was employed in establishing many of the annotations, but allowed sequenced-based inference indirectly via phylogenetic profiles and matches to protein sequence patterns. The complete data collection is available from the MouseFunc I website [30].

**Table 1:** Data collection description: summary of the data sources

Data type	Description	Representation
Gene expression	Expression data from oligonucleotide arrays for 13,566 genes across 55 mouse tissues (Zhang *et al*. [21])	Median-subtracted, arcsinh intensity measurements
	Expression data from Affymetrix arrays for 18,208 genes across 61 mouse tissues (Su *et al*. [44])	gcRMA-condensed intensity measurements
	Tag counts at quality 0.99 cut-off from 139 SAGE libraries for 16,726 genes [45]	Average and total tag counts
Sequence patterns	Protein sequence pattern annotations from Pfam-A (release 19) for 15,569 genes with 3,133 protein families [46]	Binary annotation patterns
	Protein sequence pattern annotations from InterPro (release 12.1) for 16,965 genes with 5,404 sequence patterns [47]	Binary annotation patterns
Protein interactions	Protein-protein interactions from OPHID for 7,125 genes [28] (downloaded on 20 April 2006)	Binary interaction patterns and shortest path between genes
Phenotypes	Phenotype annotations from MGI for 3,439 genes with 33 phenotypes [48] (downloaded on 21 February 2006 from [49])	Binary annotation patterns
Conservation profile	Conservation pattern from Ensembl (v38) for 15,939 genes across 18 species [50]	Binary conservation patterns and conservation scores
	Conservation pattern from Inparanoid (v4.0) for 15,703 genes across 21 species [51]	Binary conservation patterns and Inparanoid scores
Disease associations	Disease associations from OMIM for 1,938 genes to 2,488 diseases/phenotypes [52,53] (downloaded on 6 June 2006 from [54])	Binary annotation patterns

gcRMA, robust multi-array analysis with background adjustment for GC content of probes; OMIM, Online Mendelian Inheritance in Man; OPHID, Online Predicted Human Interaction Database; SAGE, serial analysis of gene expression.

To integrate these diverse data sets and associate them with functional annotations, we mapped the gene (or gene product) identifiers used in each data set to a common set of Mouse Genome Informatics (MGI) gene identifiers (as defined 21 February 2006), which are, in turn, associated with Gene Ontology (GO) terms curated by MGI [31,32]. Thus, annotations for each gene were the union of annotations made to the set of the gene products for that gene. We excluded GO annotations based solely on the 'inferred from electronic annotation' (IEA) evidence code, since many of these annotations are themselves computational predictions that have not been reviewed by a curator [33]. We also excluded GO terms with too few training examples, that is, those annotated to fewer than three genes in the training set, expecting that it would be extremely difficult for current classifiers to deal with such a limited number of positive training examples. To focus on predictions most likely to suggest specific follow-up experiments, we considered only GO terms associated with 300 or fewer mouse genes in the training set. (This threshold was chosen by manually examining GO terms ranked in descending order by the number of genes currently annotated to each term, and subjectively assessing whether predictions of that GO term would immediately suggest a follow-up validation experiment.) The final data collection contained information on 21,603 MGI genes, of which 8,506 were associated with at least one of the 2,815 individual GO terms we considered.

An invitation to participate in this assessment was circulated among research groups known to work in gene function prediction. Nine groups ultimately participated by submitting predictions. (For a brief description of the methods used by each, see Table 2; for more details see Additional data files 20 and 21.) The data and annotations were distributed in a form intended to prevent participants from using additional data sources, and to enable cross-validation. First, data were distributed to participants in an 'anonymized' form, with each MGI gene identifier replaced with a randomly generated identifier and presented to participants in permuted order. Thus, participants made predictions without knowing the gene identities or any gene information outside the training data. Second, annotations were omitted for a randomly selected 10% of genes (the 'held-out set').

**Table 2:** Brief description of function prediction methods used

Submission identifier	Approach	Name	Author initials
A	Compute several kernel matrices (SVM) for each data matrix, train one GO term specific SVM per kernel, and map SVMs' discriminants to probabilities using logistic regression	Calibrated ensembles of SVMs	GO, GL, JQ, CG, MJ, and WSN
B	Four different kernels are used per data set. Integration of best kernels and data sources is done using the kernel logistic regression model	Kernel logistic regression [55]	HL, MD, TC, and FS
C	Construct similarity kernels, assign a weight to each kernel using linear regression, combine the weighted kernels, and use a graph based algorithm to obtain the score vector	geneMANIA	SM, DW-F, CG, DR, and QM
D	Train SVM classifiers on each GO term and individual data sets, construct several Bayesian networks that incorporate diverse data sources and hierarchical relationships, and chose for each GO term the Bayes net or the SVM yielding the highest AUC	Multi-label hierarchical classification [56] and Bayesian integration	YG, CLM, ZB, and OGT
E	Combination of an ensemble of classifiers (naïve Bayes, decision tree, and boosted tree) with guilt-by-association in a functional linkage network, choosing the maximum score	Combination of classifier ensemble and gene network	WKK, CK, and EMM
F	Code the relationship between functional similarity and the data into a functional linkage graph and predict gene functions using Boltzmann machine and simulated annealing	GeneFAS (gene function annotation system) [2,3]	TJ, CZ, GNL, and DX
G	Two methods with scores combined by logistic regression: guilt-by-association using a weighted functional linkage graph generated by probabilistic decision trees; and random forests trained on all binary gene attributes	Funckenstein	WT, MT, FDG, and FPR
H	Pairwise similarity features for gene pairs were derived from the available data. A Random Forest classifier was trained using pairs of genes for each GO term. Predictions are based on similarity between the query gene and the positive examples for that GO term	Function prediction through query retrieval	YQ, JK, and ZB
I	Construct an interaction network per data set, merge data set graphs into a single graph, and apply a belief propagation algorithm to compute the probability for each protein to have a specific function given the functions assigned to the proteins in the rest of the graph	Function prediction with message passing algorithms [57]	ML and AP

AUC, area under the receiver operating characteristic curve; GO, Gene Ontology.

Each group developed and implemented their prediction methodology independently. Each submission was required, for each gene-GO term combination, to include a score (ranging from 0 to 1) reflecting prediction confidence. The data collection was released in July 2006 (with GO annotations obtained from the GO website on 17 February 2006; version 1.612). Initial prediction results were submitted in October 2006, with seven groups submitting complete prediction sets. After viewing performance measures (but not gene identities or information on the veracity of any specific prediction), it was noted that some groups did not provide a complete set of predictions; also, one group withdrew their predictions upon discovering a coding error. In an effort to increase the number and quality of submitted predictions, all groups were given the opportunity to alter their methods and submit new predictions for a second December 2006 deadline, and five groups did so.

### Performance evaluation

To evaluate each set of predictions, we first used the set of held-out genes. GO annotations are an evolving target (annotations are continuously added, deleted, and modified), which enabled us also to perform a prospective evaluation. For this purpose, we also identified the set of genes that had newly acquired an association to a GO term during the eight months since downloading of the version of MGI GO annotation used in training. The GO annotations used for prospective evaluation were obtained from the GO website on 20 October 2006 (version 1.641). To obtain a baseline performance against which to compare predictions from each approach, we employed a naïve Bayes 'straw man' approach. To train this 'straw man' classifier, we used the six sets of binary gene features that are natively in the (gene, property) form, and did not use feature selection (Additional data file 21). We assessed success for each GO term using area under the receiver operating characteristic (ROC) curve (AUC) [34]; precision was assessed at several fixed recall values (all measures used are defined in Materials and methods). For evaluation purposes, we grouped GO terms in twelve evaluation categories corresponding to all combinations of the three GO branches - Biological process, Molecular function, or Cellular component - with four ranges of 'specificity', that is, the number of genes in the training set with which each term is annotated ({3-10}, {11-30}, {31-100}, and {101-300}).

**Figure 1:**
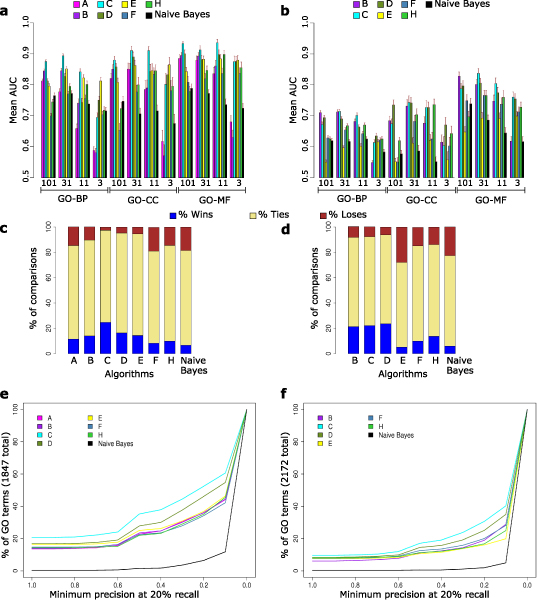
Measures of performance for the initial round of GO term predictions. **(a) **Mean area under the receiver operating characteristic curve (AUC) within each evaluation category, evaluated using the held-out genes. Gene Ontology Biological process (GO-BP), Cellular component (GO-CC), and Molecular function (GO-MF) branches are indicated on the x-axis, grouped by specificity (indicated by the minimum number of genes in the training set associated with each GO term in a given category). Upper case letters associated with the color code correspond to submission identifier. **(b) **Mean AUC within each evaluation category, evaluated prospectively using newly annotated genes. **(c) **For each pair of submissions X and Y, we test for difference in AUC value for every GO term (evaluated using held-out genes). Color bars indicate fraction of pairwise comparisons for which X's AUC is significantly higher (blue), not significantly different (beige), and significantly lower (maroon). **(d) **As (c), except evaluated using the newly annotated genes. **(e) **The fraction of GO terms exceeding the indicated precision at 20% recall (P20R) value, evaluated using held-out genes. The black line corresponds to the fraction of GO terms for which the 'straw man' approach achieved the indicated precision. **(f) **As (e), except with P20R values derived prospectively from newly annotated genes.

Figure 1 shows some performance measures of the first round of submissions. Note that team I submitted partial results and was, therefore, not assessed for overall performance in each evaluation category. Team E's results for the prospective evaluation were based on a partial implementation of their algorithm (see details in Additional data file 20, Box 5). Figure 1a,b shows the mean AUC of GO terms within each evaluation category, evaluated using the held-out and newly annotated genes, respectively. Figure 1c,d shows for each submission how often its AUC value was significantly better (or worse) than the AUC value of another submission. We assessed significance of difference in AUC between two submissions for each GO term (α = 0.05) using a Z-test [34].

In this analysis, most submissions beat the 'straw man' in all categories (both by mean AUC and by number of wins and losses); however, the overall differences among groups were not dramatic. (See Additional data file 1 for a summary of the number of significant wins and losses per evaluation category.) The complete set of performance measures evaluated with the held-out gene set may be found in Additional data file 7 (initial predictions) and Additional data file 9 (revised predictions), while the corresponding prospective evaluation results may be found in Additional data files 8 and 10. Performance measures reported here are conservative in the sense that false positive predictions (genes predicted as having a GO term that were not currently annotated with that GO term) may actually be correct but not yet annotated as such.

In contrast to AUC, the precision at fixed recall values was dramatically higher for all submissions than for the 'straw man'; Figure 1e,f shows the proportion of GO annotations reaching various precision values at 20% recall (a threshold selected as 'midrange' for display). Additional data file 2 shows the mean precision at 20% recall for GO terms within each evaluation category, evaluated using both held-out and newly annotated genes. Due to the small number of positives (genes carrying a given annotation) relative to negatives (genes that do not carry the annotation), this characteristic would usually be reflected only in the very left part of the ROC, and is not generally captured by the more commonly used AUC measure. However, precision is a more relevant measure to many end users, since it reflects the proportion of validation experiments for top-scoring predictions that would prove successful.

Performance of all submissions differed markedly depending on whether evaluation was on the held-out genes or on newly annotated genes (Figure 1a,c,e compared with Figure 1b,d,f), suggesting that emerging annotations are qualitatively different from a random sample of previously existing annotations - a variable that is only rarely considered in large-scale predictions of gene function.

In fact, the main type of evidence supporting the annotations differs between the new and the held-out annotations; while 50% and 2.5% of newly acquired annotations were derived from sequence or structural similarity (ISS) and reviewed computational analysis (RCA), respectively, the corresponding proportions for held-out annotations were 9% and 31% (Additional data file 3).

**Figure 2:**
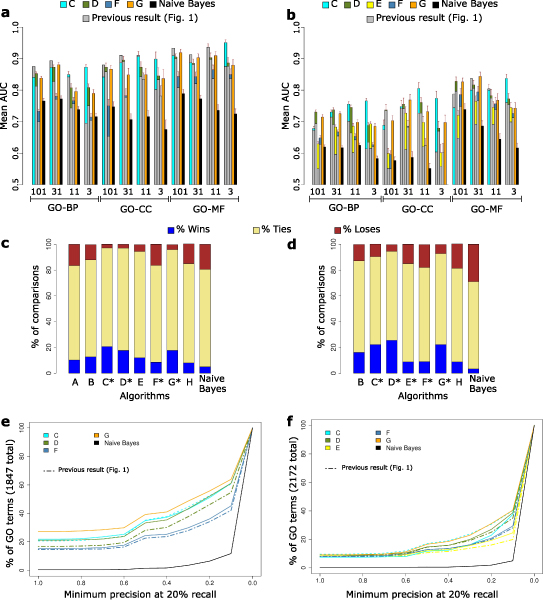
Measures of performance for the second round of GO term predictions. **(a, b) **As described in Figure 1a, b, except that the gray color area indicates performance in the first set of submissions. **(c-f) **As described in Figure 1c-f, except that asterisks in (c) and (d) indicate second-round submissions and dashed lines in (e) and (f) indicate the performance of an earlier submission by the same group. GO, Gene Ontology.

Figure 2 shows the performance of the second round of submissions (Additional data file 2). In most cases, revised predictions slightly outperform the original ones. All subsequent analyses described here used only one submission per group, choosing the most recent where there were two submissions. The complete evaluation results are available from the MouseFunc I website [30].

### Factors affecting prediction performance

**Figure 3:**
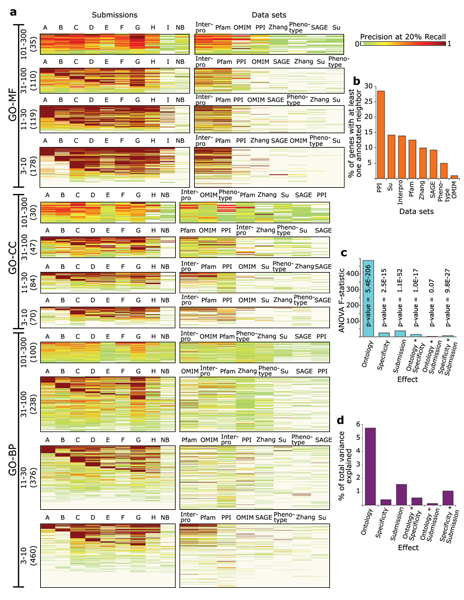
Factors affecting prediction performance. **(a) **Precision at 20% recall (P20R) values evaluated using held-out annotations on all Gene Ontology (GO) terms (vertical axis) within each of the 12 evaluation categories for each submission (left panel) and for a simple guilt-by association using each data set in turn as its sole evidence source (right panel). The number of genes in each evaluation category is shown in parentheses. GO-BP, GO Biological process; GO-CC, GO Cellular component; GO-MF, GO Molecular function; NB, naïve Bayes. Data sets are described in Table 1. **(b) **Fraction of the 21,603 genes in the data collection with at least one annotated neighbor per data set. **(c) **Analysis of variance (ANOVA), exploring the effects of various factors on P20R values. **(d) **Fraction of total variance in P20R values that is explained by each effect. Asterisks in (c, d) indicate interaction between two factors.

To ask whether some data sets were more useful than others, and how their value might vary among evaluation categories, we applied a simple guilt-by-association approach similar to a previously described method [35]. The confidence score for gene X and GO term Y is simply the number of 'neighbors' of X that are currently annotated with Y (see Materials and methods). We evaluated performance after applying this method to only one data set at a time. Figure 3a shows precision at 20% recall (P20R) values obtained by each submission on every GO term, and by using each one of the data types as input to the guilt-by-association approach. A striking observation is that protein sequence pattern annotations are the most predictive data type overall and are especially useful for predicting Molecular function GO terms. Expression data, and phenotype and disease associations are important contributors for more general Cellular component and Biological process GO terms. Moreover, interaction data comprise a remarkably useful evidence source, considering that only a small proportion of protein interactions in mammals is known. Figure 3a also indicates that hard to learn GO terms are the ones where there is absence of predictive power in all data types. This is especially clear in the specificity range {3-10} in all GO branches. We also examined maximum coverage (number of genes present in a given data set with at least one annotated 'neighbor' when using the simple guilt-by-association method), noting that this coverage allowed functional associations for at most 30% of the 21,603 genes to be predicted given any single data set (Figure 3b).

Analysis of variance (ANOVA; Additional data file 11) verified what is clear from Figures 1a,b, 2a,b and 3a; the branch of the ontology is the main factor to explain variance in performance as shown in Figure 3c,d. Biological process GO terms, which reflect what biologists would typically consider to be physiological function of genes and most related to phenotypes, are apparently more difficult to predict than Molecular function or Cellular component terms. As expected, more specific GO terms in each evaluation category were more difficult to predict.

To explore whether there were commonalities in pattern of performance among the submissions, we examined the correlations among P20R values and grouped the submissions using hierarchical clustering (Additional data file 4). We identified three pairs of submissions that were grouped together by several correlation measures (data not shown). These pairs of submissions were ('F', 'G'), ('A', 'B'), and ('C', 'D'). Submissions 'F' and 'G' both employ functional linkage, while submissions 'A' and 'B' are mainly kernel-based methods. (Despite the fact that submissions 'E' and 'I' also used functional linkage, their results were uncorrelated with 'F' and 'G'.) Submissions 'A', 'B', 'C', and 'D' each used weighted combinations of diverse data sets, but neither 'A' nor 'B' gave highly correlated results with 'C' or 'D'. Since all participant methods combine several algorithms, require the use of multiple parameters, and vary the procedure for feature design and selection, it is not surprising that differences in results cannot be simply attributed to any one algorithmic choice.

To assess the stability of the prediction performance, we measured the performance variability in five randomly chosen subsets of the training data and measured the standard deviations of AUC and P20R performance measures within each evaluation category. The median standard deviations of AUC and P20R across all evaluation categories were 0.01 and 0.02, respectively, suggesting that our performance measures were robustly determined (Additional data file 12).

One of the major challenges in training a classifier is overfitting, that is, generating models that precisely fit the idiosyncrasies of training data at the expense of their accuracy when applied to new data. We assessed overfitting using a standard approach - examining the extent to which performance estimates are exaggerated when one calculates them based on the training data rather than on the held-out test set (Additional data file 12). For example, Biological process GO terms with specificity {31-100} had a mean P20R value that was increased by a factor of 1.3 (averaged over all submissions) when it was calculated based on the training data rather than the held-out gene set.

We note that submissions 'C', 'D' and 'G' are among the top performers on most evaluation categories by various measures. The performance of submission 'C' was particularly strong with respect to AUC. Submission 'D' performs stably across the range of the number of genes annotated to each GO term and its performance was especially good for prospective predictions. Submission 'G' has a strong performance in precision across a range of recalls (Additional data files 5 and 6). Submission 'E' and 'H' perform better for the most specific evaluation categories. Thus, different methods had different strengths and no prediction method was clearly superior by every criterion.

### Integration of submissions in a single set of predictions

**Figure 4:**
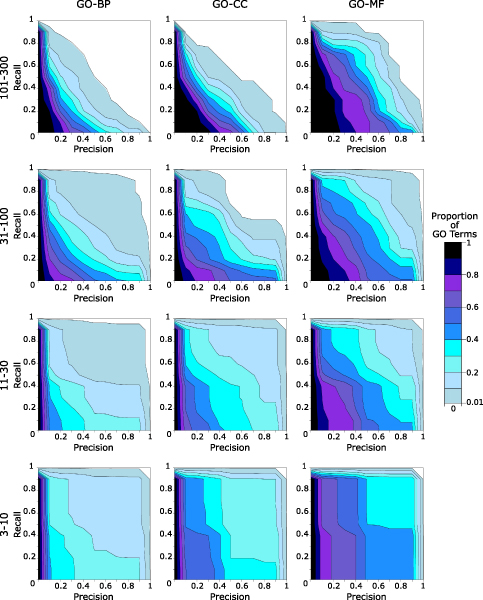
Distribution of GO terms at several precision/recall performance points. Proportion of Gene Ontology (GO) terms per evaluation category with a precision/recall performance point that is both above and to the right of a given precision/recall point in the contour plots. GO-BP, GO Biological process; GO-CC, GO Cellular component; GO-MF, GO Molecular function.

To simplify subsequent analyses for ourselves and other investigators, we derived a single set of prediction scores from the set of submitted scores. We unified the independent submissions for each evaluation category by adopting the scores from the submission with the best P20R value for that evaluation category (evaluated using held-out genes). The combined predictions averaged 41% precision at 20% recall with 26% of GO terms having a P20R value greater than 90%. Figure 4 indicates the proportion of GO terms at different precision and recall values. (Also see Additional data file 19; Additional data file 13 lists the precision achieved by the unified predictions at several recall values for each GO term.) To put this prediction performance into perspective, random predictions for a GO term with 30 genes left to be identified would be expected to yield a P20R value of 0.15%. In addition, these precision estimates are conservative since many predictions may ultimately prove correct despite not being currently annotated.

### Impact of predictions among GO terms for which precision can be well estimated

**Figure 5:**
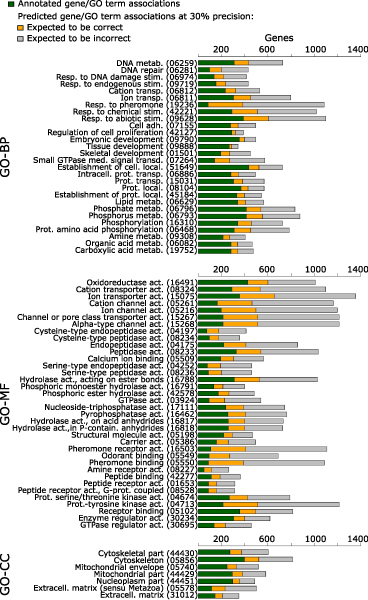
Number of high-precision predictions among GO terms for which precision can be confidently estimated. Number of currently annotated (green) versus predicted genes (orange, predictions expected to be correct; gray, predictions expected to be incorrect) for a subset of Gene Ontology (GO) terms for which 30% precision on held-out annotations was achieved while recovering at least 10 positives in the held-out set. The number of predicted genes displayed was limited to 1,000. GO terms were ordered according to similarity of prediction/annotation patterns. Terminal digits of GO term identifiers are shown in parentheses. GO-BP, GO Biological process; GO-CC, GO Cellular component; GO-MF, GO Molecular function.

To gain insight into the potential impact of predictions on the current state of gene function annotation, we more closely examined a subset of GO terms in the unified set of predictions. For each GO term, we established the lowest score at which a precision of 30% or better was achieved while recovering at least 10 true positives within the held-out test set (allowing precision to be well estimated). There were 71 GO terms with predictions meeting this criterion (tending to be the less specific GO terms due to the number of required positive genes in the training set). Figure 5 shows the number of currently annotated and predicted genes for each GO term, including 9,429, 2,087, and 19,849 predictions in the Biological process, Cellular component, and Molecular function branches, respectively. (The maximum number of predictions displayed was limited to 1,000.) This figure illustrates the potential future impact of these predictions on the state of function annotation should the expected 30% or more of these predictions prove true.

While Figure 5 shows the impact for more general GO terms, we note that performance for more specific GO terms was also quite good. For example, the mean P20R from the best-performing submission for the most specific {3-10} versus least specific {101-300} category was 21% versus 37%, 38% versus 50%, and 51% versus 53% for Biological process, Cellular component, and Molecular function branches, respectively. Thus, predictions for more specific GO terms offer a similarly high impact on current function annotation (and there are many more specific GO terms than general GO terms).

Predictions have varying degrees of novelty, ranging from 're-predictions' and 'refinement predictions' to 'highly novel'. Re-predictions are cases in which the gene is currently annotated with that GO term based solely on IEA evidence; these are often unverified predictions made previously by others. Refinement predictions are cases in which the gene is currently annotated with an ancestor of the predicted GO term. We describe all other predictions as 'highly novel'. Among the number of predictions displayed in Figure 5, the percentages of refinements are 18%, 21%, and 17% for Biological process, Cellular component, and Molecular function branches, respectively, while the percentages of re-predictions are 43%, 37%, and 32%. Thus, 3,677 (39%), 877 (42%), and 10,123 (51%) predictions for Biological process, Cellular component, and Molecular function branches, respectively, were highly novel.

### Literature evaluation for top-scoring predictions with a high degree of novelty

To gain intuition into the quality of those predictions with the highest degree of novelty, we performed a focused literature analysis on highly novel top-scoring predictions. For this, we identified the top three predictions from each of the twelve evaluation categories, excluding re-predictions and refinement predictions.

To avoid over-weighting particular GO terms or genes, we also allowed only one prediction per evaluation category for any given gene or GO term. Investigators with extensive experience with literature curation and knowledge of mouse gene function (DPH and JAB) examined published literature relating to these 36 high-scoring highly novel predictions, and scored each prediction according to the nature of published evidence. Additional data file 14 contains the list of highly novel predictions investigated.

Out of the 36 high-scoring predictions examined, 21 (58%) were found to be true or likely to be true based on experimental data reported in the literature. Since six other cases could neither be confirmed nor refuted by current literature, we estimate that the true precision for top novel high-scoring predictions lies between 58% and 75%. Of the 21 found to be true, 9 (43%) were strongly supported but were not annotated simply because the literature had not yet been curated. For example, annotation of the gene encoding Slfn8 (schlafen 8) with the GO term 'negative regulation of cell proliferation' is supported [36], with evidence corresponding to the inferred from direct assay (IDA) evidence code [33]. This gene currently does not have any functional annotation in the MGI system, and thus exemplifies the novel assignment of function to unannotated genes.

Other reasonable annotations identified in this set of 36 examples include 12 cases where the genes are members of characterized gene families. It is likely that the genes play at least a similar role as predicted, although the evidence is not strong enough to support the annotation using GO Consortium annotation policy. An example of this is the mouse gene 4930430D24Rik, which is predicted to be involved in biological process 'protein amino acid methylation'. This gene is defined solely by cDNA clone data and has no experimental information associated with it. However, it has sequence similarity with the gene encoding Btg1, which has been documented as interacting with protein methyl transferases.

Another 6 cases (17%) of the 36 examined could be neither confirmed nor refuted by current literature. For example, the gene *Klhl12 *(encoding Kelch-like 12) was associated with the cellular component term 'stress fiber'. This gene is homologous to members of the kelch family of genes found in *Drosophila*. The *Drosophila *gene products are found in a variety of cellular locations. Although some members of this family regulate stress fiber formation through the Ras pathway, there is evidence that the human ortholog binds proteins in a variety of locations and that this protein functions in the context of the ubiquitin E3 ligase complex. As a result, we currently cannot infer cellular location of this gene product and thereby judge the prediction.

The remaining 9 (25%) of the 36 predictions examined were considered to be incorrect based on current literature (see Additional data file 14 for the list of predictions investigated). For example, the gene *Grm4 *(encoding the metabotropic glutamate receptor 4) is predicted to have the molecular function 'calcium channel regulator activity'. However, although other G protein coupled receptors regulate calcium levels, there is no current evidence that this gene functions in this way.

Taken together, these results suggest that high-scoring predictions based on large-scale data integration comprise a promising resource to guide both curators and experimentalists to correct hypotheses about gene function in mammals.

### A resource for browsing predictions and underlying evidence

So that researchers may browse predictions and gain intuition about evidence that underlies predicted annotations, an online resource allowing browsing by GO term or gene is available [37]. To facilitate follow-up experimental study, this resource contains links to existing Gene Trap alleles available as heterozygous mouse embryonic stem cell lines.

### Illustration of the evidence underlying predictions for two GO terms

**Figure 6:**
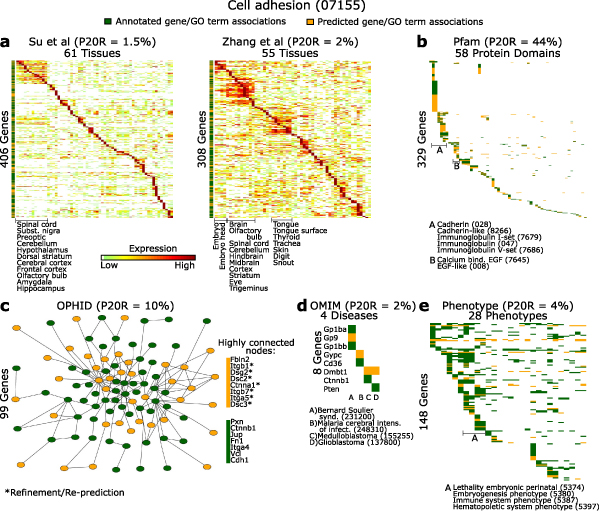
Illustration of evidence underlying predictions for the GO term 'Cell adhesion'. As an assessment of predictive usefulness, the precision at 20% recall (P20R) value based on each single data source is shown in parentheses. **(a) **Expression levels of annotated genes (dark green) and predictions (orange), grouped by Pearson correlation and complete-linkage hierarchical clustering. **(b) **Protein domains in common among predictions and annotated genes. **(c) **Largest protein-protein interaction network among predictions and annotated genes. OPHID, Online Predicted Human Interaction Database. **(d) **Disease and **(e) **phenotype annotations in common between predictions and annotated genes. Terminal digits of identifiers are shown in parentheses. OMIM, Online Mendelian Inheritance in Man.

**Figure 7:**
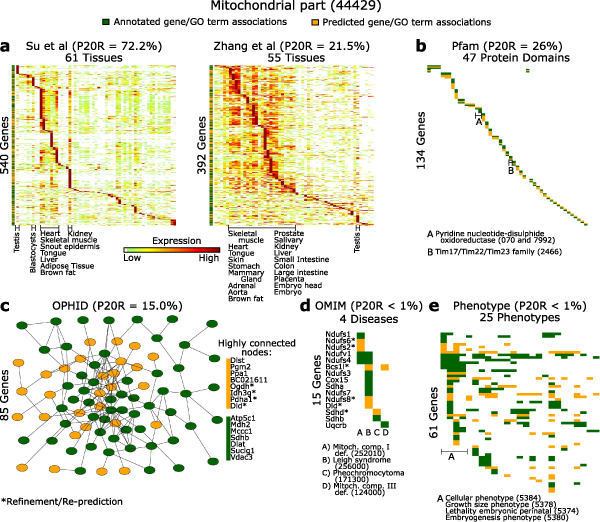
Illustration of evidence underlying predictions for the GO term 'Mitochondrial part'. **(a-e) **As described in Figure 6a-e. GO, Gene Ontology.

To gain insight into the prediction process and the nature of supporting evidence, we examined predictions for two specific GO terms in greater detail. Genes currently annotated with 'Cell adhesion' (Figure 6) and 'Mitochondrial part' (Figure 7) are shown together with genes newly predicted to have these GO terms, in the context of supporting evidence. These GO terms were chosen to illustrate different facets of biology and the utility of multiple data types. Based on the predictive power of each data source in isolation, protein sequence pattern annotations are the most useful source to predict genes involved in cell adhesion, while gene expression data are more relevant for predictions of mitochondrial part. (The value of each data set is based on precision of predictions at 20% recall based solely on that data set, considering genes present in each data set.)

To further validate mitochondrial part predictions, we asked if mitochondrially localized proteins (according to [38]) were enriched among mitochondrial part predictions. Indeed, out of 108 mitochondrial part predictions with available data [38], 83 were mitochondrially localized (*P *= 2.3 × 10^-7^; cumulative hypergeometric test). Additional data file 15 contains mitochondrial part predictions with available mitochondrial localization data [38].

Figures 6 and 7 illustrate that, as intuitively expected, the patterns of expression and other data types among genes annotated and predicted in these categories are quite similar. In addition, the graph formed by protein interactions among annotated and predicted genes contains a connected component (that is, a subset of nodes that are mutually connected by some path) that is larger than expected by chance (*P *< 0.0001; based on a permutation test of 10,000 random networks). Collectively, this figure illustrates the origin of predictions within diverse genomic and proteomic evidence (see Additional data files 16 and 17 for the data underlying Figures 6 and 7).

## Discussion

Prediction confidence scores fall along a continuum from 0 (predicted not to be true) to 1 (predicted to be true). Whether a score between 0 and 1 should be treated as a prediction for or against the annotation (or as a non-prediction) depends on the user's application-dependent trade-off between precision and recall, and an expert biologist may wish to filter the list further based on their knowledge and intuition before proceeding to carry out experiments. Users performing medium-scale genomic experiments may favor recall over precision and select predictions using a higher recall threshold where the search space (and costs) will be reduced without losing recall. Alternatively, users requiring higher precision can take only the top few predictions.

The performance differences among the methods examined here could have a substantial practical impact. For example, suppose a user plans to order ten mouse mutant strains at a cost of $10,000 each to assay a physiological phenotype caused by 20 unidentified genes. Since the combined predictions averaged 41% precision at 20% recall, the user may expect to see four mutants showing the expected phenotype at a cost of $25,000 per successful experiment; on the other hand, if a simple guilt-by-association approach having only one source of evidence as input (with average precision at 20% recall of 10%) is used to select the genes to assay, the user may expect to see only one mutant with the desired phenotype at a cost of $100,000 per successful experiment.

Annotation efforts such as FANTOM [39] have populated a high-quality reference database of function assignments in which each annotation is highly likely to be true. This encyclopedic approach is valuable, but necessarily discards partial information, or 'medium-confidence' predictions. A full spectrum of confidence measures can serve as a form of principled triage, in which experimentalists are guided towards those hypotheses that are more likely to prove true but which have not yet been proven. Furthermore, quantitative function prediction should also prove useful as a resource to assist more qualitative encyclopedic efforts.

Variation in performance between submissions is more substantial when the evaluation criterion is precision at a given recall, rather than AUC, as shown in Figure 3. The variation in performance between groups and between first and second submissions from the same group indicates that, as a community, we have not yet converged on an asymptotic limit to performance. Also, ANOVA results indicate that GO branch is a greater contributor to variation in performance than the prediction method used. The difficulty of predicting GO terms is highest in the Biological process branch followed by the Cellular component and then Molecular function branches. Also, the difficulty decreases as the number of genes currently annotated to that GO term increases.

Our assessment indicates that many submissions were more successful in predicting for held-out genes than for the newly annotated set of genes. This suggests the problem of predicting novel annotations may be qualitatively different from the problem of predicting previously known but held-out annotations. Approximately 50% of new annotations were annotated on the basis of sequence or structural similarity (evidence code ISS; Additional data file 3), as opposed to 9% for held-out annotations. This indicates that a greater proportion of recent annotations has been made by transfer of annotation from other species via homology.

Although we considered homology to proteins in other species through phylogenetic profiling and use of protein domain matches, we did not allow transfer of functions from other species via orthology for several reasons. First, function transfer by orthology is the most mature method for function prediction and we consider that the need is greatest to improve methods that integrate and analyze newer large-scale experimental data types. Second, use of GO annotation from other species would have rendered our cross-validation performance estimates uninterpretable by allowing circular predictions. For example, a held-out mouse GO annotation that had previously been transferred by homology from a mouse gene to a human gene might then be transferred back to mouse as a 'prediction'. Third, a function determined in a single organism can quickly spread via orthology to many organisms so that a single piece of evidence might be overcounted as an independent fact in multiple organisms. The second and third issues might be circumvented by only considering annotation from other species based on experiments carried out in that organism. While some evidence codes in GO annotations indicate within-organism support (for example, IDA, IMP [inferred from mutant phenotype], IEP [inferred from expression pattern], IPI [inferred from physical interaction]), other evidence codes such as TAS [traceable author statement], NAS [non-traceable author statement], ISS, and RCA are ambiguous [33]. Careful curation of the organism from which function annotation evidence has been derived would greatly facilitate the use of orthology-based function transfer in future integrative studies.

We found that submissions from every group were subject to overfitting in most GO categories. While the presence of overfitting is not surprising given the paucity of available training data, it does suggest that future performance gains will come from classifier training methodology that further limits overfitting. Another future improvement to predictions might be a unified score based on all submissions, via an ensemble or 'combination of experts' method [40]. In addition, to facilitate interpretation, scores might be transformed to accurately reflect the probability that a prediction is correct. Another possible improvement would be the use of a more refined subset of GO terms as gold standard. For example, predictions could be judged according to a reduced subset of GO terms that are relatively independent of one another and each specific enough to suggest a follow-up experiment [24]. Furthermore, to improve prediction accuracy in future function prediction efforts, data sources containing additional evolutionary, structural, enzymatic and sequence similarity information might be integrated. It would also be interesting to perform a factorial analysis on variations of the classifiers that performed best here, in order to obtain biological intuition or insight into why these classifiers performed well. Our prediction effort was focused on identifying 'errors of omission' in GO annotation. It would also be worthwhile to explore whether low prediction scores for current annotations (apparently 'false negatives') could be useful in recognizing erroneous functional annotations ('errors of commission').

A major implication of our analysis is that protein sequence patterns from Pfam and InterPro are extremely useful evidence sources not only for Molecular function GO terms (as expected, since these primarily reflect biochemical activities) but also for inference of Cellular component and Biological process terms. This trend may be due, in part, to the incorporation of biochemical terms in the Biological process ontology (for example, 'protein amino acid phosphorylation' is listed as a Biological process, and its known members overlap with 'protein kinase activity', which is a Molecular function) as well as the fact that protein sequence patterns do relate to substrates associated with specific physiological processes and cellular compartments (for example, DNA-binding proteins are primarily found in the nucleus). Nevertheless, we note that the proportion of genes with protein sequence pattern annotations is much lower in the 8,851 unannotated genes (62%; this includes genes with annotations based solely on IEA evidence) than it is among the 12,752 annotated mouse genes (90%) in the data collection. This indicates that sequence features may be less useful in future predictions of function for currently uncharacterized genes. This is particularly true of Biological process terms, which are the least predictable using sequence features alone, and conceptually most closely related to phenotype. In future, it will be valuable to predict phenotypes as well as functions. Phenotype predictions are immediately testable, and phenotype data in mammalian organisms and cell culture models have a rapid rate of emergence that will permit prospective evaluation of predictions.

## Conclusion

We performed a systematic evaluation of diverse, independently developed computational approaches for predicting gene function from heterogeneous data sources in mammals. The results show that currently available data for mammals allow predictions with both breadth and accuracy. At a recall rate of 20%, a unified set of predictions averaged 41% precision, with 26% of GO terms achieving a precision better than 90%. Predictions with comparable precision have been successfully used in yeast [41]. A striking finding is that predictions for GO terms in the most specific evaluation category (ten or fewer annotated genes) have a precision comparable to that obtained in the more general evaluation categories. For Biological process GO terms, we achieved a mean precision at 20% recall for blinded predictions ranging from 28% to 46%, depending on evaluation category specificity. Corresponding performance for Cellular component and Molecular function terms was even higher, ranging from 38% to 58% and from 56% to 64%, respectively. Importantly, many highly novel function predictions emerge for the 38% of mouse genes that remain uncharacterized.

## Materials and methods

### Performance statistics

To assess performance of function predictions by each method, we obtained the ROC curve and the AUC for each GO term using the trapezoidal rule [42]. (The AUC corresponds to the probability that a random positive instance will be scored higher than a random negative instance.) For this assessment, GO annotations were up-propagated. That is, if a gene is associated with a GO term, then this gene is also associated with all the ancestor GO terms of that GO term. During evaluation, refinement predictions are considered false positives.

We assessed whether observed differences in AUC between submissions X and Y were statistically significant [34] and computed the precision at various recall rates as previously described [43]. Precision is defined as the number of genes correctly classified as having a given GO term divided by the total number of genes classified as having that GO term $\left(\frac{TP}{TP+FP}\right)$. Recall is defined as the percentage of genes annotated with a given GO term that were classified as having that GO term $\left(\frac{TP}{TP+FN}\right)$. Other performance measures included the AUC up to the first 50 false positives, and the recall obtained at 1% false positive rate. False positive rate is defined as the fraction of genes not annotated with a given GO term that were classified as having that GO term $\left(\frac{FP}{FP+TN}\right)$. Tables with the median, mean and standard deviation of all performance measures over the GO terms in each evaluation category are provided for each submission (Additional data files 7 to 10).

### Assessing the predictive value of each data type

To determine the value of each data type in predicting function, we used the following simple guilt-by-association method; for protein-protein interaction data, we counted the number of times each GO term is annotated among direct interaction partners ('neighbors'). For data sets composed of binary gene features, we considered the neighbors of gene X to be those genes annotated to have the same specific feature, for example, a specific phenotype, disease association, or protein sequence pattern annotation. In the case of non-binary data, for example, expression or phylogenetic profile, neighbors are genes that correlate with X (Pearson correlation coefficient > 0.5). After determining the neighbors of each gene, we sum for each GO term, based on the type of data, either the correlation coefficients, or the number of shared features per neighbor, or the number of the neighbors annotated with GO term X. This value is then used as a score of the function prediction. The contribution of each data set is then assessed considering genes with at least one annotated neighbor in the data set. Tables with the median, mean, and standard deviation of the performance measures over GO terms in each evaluation category per data set are provided in Additional data file 18.

### Score transformation

Since scores were not necessarily calibrated across GO terms, we developed a monotonic transformation to make scores for different GO terms more comparable. Letting *n *be the total number of genes considered, *t *be the number of existing positive annotations for the current GO term, and *s*_*i *_be the un-calibrated score for the *i*^th ^gene, the calibrated score for the *i*^th ^gene $s_{i}^{*}$ is defined as: $s_{i}^{*}=\frac{L\cdot s_{i}}{L\cdot s_{i}-s_{i}+1}$ where *L *is the free (non-negative) parameter chosen such that $\sum_{i=1}^{n}s_{i}^{*}=t$. *L *is found separately for each GO term via a MATLAB optimization routine. After this transformation, the average score for each GO term is equal to the fraction of genes currently annotated with that GO term.

### Generating a list of high scoring novel predictions for manual investigation

To evaluate the quality of top-scoring predictions more closely, we identified the set of submitted predictions that performed best within each of the 12 evaluation categories (according to the P20R measure on held-out genes). Within each of the 12 evaluation categories, gene/term pairs were pooled and ranked by calibrated scores (described above). All currently annotated gene/term pairs were removed, resulting in a ranked list of predictions that are considered classification errors according to current GO annotations, but may in fact be correct. To focus on the highly novel predictions, we also excluded re-predictions and refinement predictions from the list.

## Abbreviations

ANOVA, analysis of variance; AUC, area under the ROC curve; GO, Gene Ontology; IDA, inferred from direct assay; IEA, inferred from electronic annotation; ISS, inferred from sequence or structural similarity; MGI, Mouse Genome Informatics; P20R, precision at 20% recall; RCA, reviewed computational analysis; ROC, receiver operating characteristic.

## Competing interests

The authors declare that they have no competing interests.

## Authors' contributions

The study was designed and organized by LP-C, TRH, and FPR, with advice from many others. LP-C assembled the data set (with help from GFB), anonymized gene identifiers in isolation from all participants, and calculated performance measures. Team A analysis was performed by GO with contributions from GL, JQ, CG, and MJ, and design and supervision from WSN. Team B analysis was performed by HL with contributions from MD and design and supervision from TC and FS. Team C analysis was performed by SM with contributions from CG, DW-F, and DR, and design and supervision from QM. Team D analysis was performed by YG and CLM with contributions by ZB, and design and supervision from OGT. Team E analysis was performed by WKK and CK with design and supervision from EMM. Team F analysis was performed by TJ and CZ with contributions from GNL and design and supervision from DX. Team G analysis was performed by MT and WT with contributions from FDG, and design and supervision from FPR. Team H analysis was performed by YQ with design and supervision from JK and ZB. Team I analysis was designed and implemented by ML and AP. Post-submission analysis was performed by LP-C, except that CLM performed ANOVA on submission performance and MT generated 'straw man' predictions and classified prediction novelty. DPH and JAB performed literature evaluation. The manuscript was prepared by LP-C, TRH, and FPR and figures by LP-C. All authors read and approved the final manuscript.

## Additional data files

The following additional data are available with the online version of this paper. Additional data file 1 is a figure showing bar graphs of pairwise comparisons of AUC within each evaluation category. Additional data file 2 is a figure showing bar graphs of mean P20R values within each evaluation category. Additional data file 3 is a figure showing bar graphs comparing properties of GO annotations in the held-out gene set, in the newly annotated gene set, and in the training set. Additional data file 4 is a figure showing a clustergram indicating Pearson correlation coefficients of the P20R performance measure among different submissions. Additional data file 5 is a figure showing heatmaps of precision at several recall values evaluated using held-out annotations on all GO terms within each of the 12 evaluation categories for each submission. Additional data file 6 is a figure showing a heatmap of median precision at several recall values evaluated using held-out annotations within each of the 12 evaluation categories per submission. Additional data file 7 is a table listing performance measures for the initial round of GO term predictions within each evaluation category evaluated using held-out genes. Additional data file 8 is a table listing performance measures for the initial round of GO term predictions within each evaluation category evaluated using the newly annotated genes (prospective evaluation). Additional data file 9 is a table listing performance measures for the second round of GO term predictions within each evaluation category evaluated using held-out genes. Additional data file 10 is a table listing performance measures for the second round of GO term predictions within each evaluation category evaluated using the newly annotated genes (prospective evaluation). Additional data file 11 is a table listing the results of the analysis of variance in prediction performance. Additional data file 12 is a table listing performance and variance on five subsets of the training data. Additional data file 13 is a table listing performance measures of the unified predictions for each GO term. Additional data file 14 is a table listing high-scoring predictions evaluated against existing literature. Additional data file 15 is a table listing mitochondrial part predictions with data from a previous study [38]. Additional data file 16 is a table listing data underlying Figure 6. Additional data file 17 is a table listing data underlying Figure 7. Additional data file 18 is a table listing performance measures for various individual evidence sources within each evaluation category evaluated using held-out genes. Additional data file 19 is a Flash animation showing a fraction of GO terms with higher precision and recall than a given precision/recall point for the unified predictions. Additional data file 20 contains a 300 word description of the function prediction method used in each submission. Additional data file 21 describes in detail the submission methods and the straw man classifier (57 pages in total).

## Supplementary Material

Additional data file 1 Bar graphs of pairwise comparisons of AUC within each evaluation category. Click here for file

Additional data file 2 Bar graphs of mean P20R values within each evaluation category Click here for file

Additional data file 3 Bar graphs comparing properties of GO annotations in the held-out gene set, in the newly annotated gene set and in the training set. Click here for file

Additional data file 4 Clustergram indicating Pearson correlation coefficients of the P20R performance measure among different submissions. Click here for file

Additional data file 5 Heatmaps of precision at several recall values evaluated using held-out annotations on all GO terms within each of the 12 evaluation categories for each submission. Click here for file

Additional data file 6 Heatmap of median precision at several recall values evaluated using held-out annotations within each of the 12 evaluation categories per submission Click here for file

Additional data file 7 Performance measures for the initial round of GO term predictions within each evaluation category evaluated using held-out genes. Click here for file

Additional data file 8 Performance measures for the initial round of GO term predictions within each evaluation category evaluated using the newly annotated genes (prospective evaluation). Click here for file

Additional data file 9 Performance measures for the second round of GO term predictions within each evaluation category evaluated using held-out genes. Click here for file

Additional data file 10 Performance measures for the second round of GO term predictions within each evaluation category evaluated using the newly annotated genes (prospective evaluation). Click here for file

Additional data file 11 Results of the analysis of variance in prediction performance. Click here for file

Additional data file 12 Performance and variance on five subsets of the training data. Click here for file

Additional data file 13 Performance measures of the unified predictions for each GO term. Click here for file

Additional data file 14 High-scoring predictions evaluated against existing literature. Click here for file

Additional data file 15 Mitochondrial part predictions with data from a previous study [38]. Click here for file

Additional data file 16 Data underlying Figure 6. Click here for file

Additional data file 17 Data underlying Figure 7. Click here for file

Additional data file 18 Performance measures for various individual evidence sources within each evaluation category evaluated using held-out genes. Click here for file

Additional data file 19 Fraction of GO terms with higher precision and recall than a given precision/recall point for the unified predictions. Click here for file

Additional data file 20 Description of the function prediction method used in each submission. Click here for file

Additional data file 21 Detailed description of the submission methods and the straw man classifier. Click here for file
