# Supplementary material for: A critical assessment of Mus musculus gene function prediction using integrated genomic evidence
Source: Genome Biol. 2008 Jun 27;9(Suppl 1):S2. doi: 10.1186/gb-2008-9-s1-s2 (PMC2447536; doi:10.1186/gb-2008-9-s1-s2)
Supplement: Additional data file 1 — Bar graphs of pairwise comparisons of AUC within each evaluation category. [file gb-2008-9-s1-s2-S1.pdf]

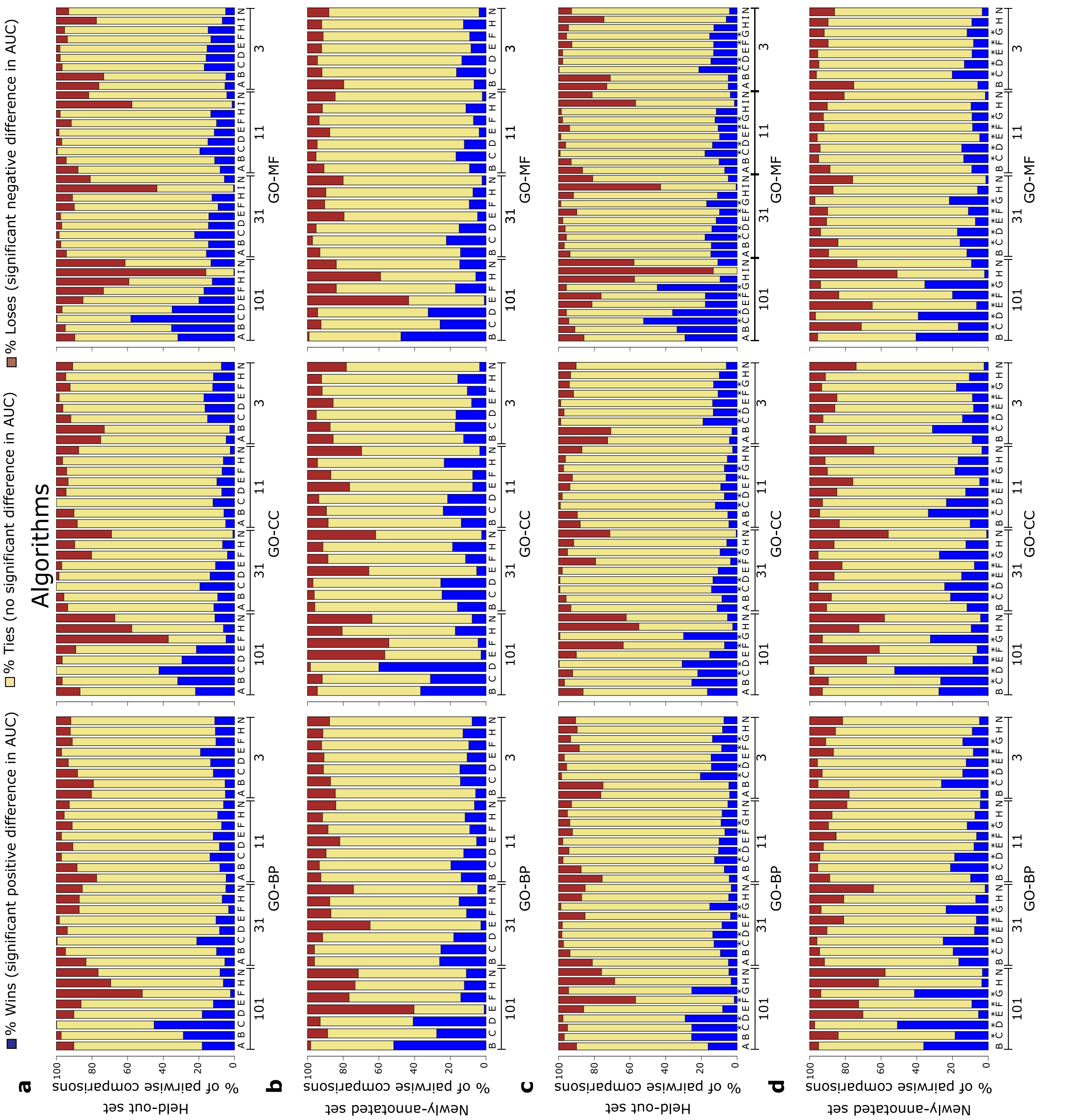

Figure S1: Pairwise comparisons in AUC within each evaluation category of GO terms. (**a**) For each pair of initial submissions X and Y, we test for difference in AUC value for every GO term within a given evaluation category, evaluated using held-out genes. Color bars indicate fraction of pairwise comparisons for which X's AUC is significantly higher (blue), not significantly different (beige) and significantly lower (maroon) than Y's AUC. (**b**) As (a), except evaluated using the newly-annotated genes. (**c**) (**d**) As (a),(b) except that asterisks indicate second-round submissions.
