# Supplementary material for: A critical assessment of Mus musculus gene function prediction using integrated genomic evidence
Source: Genome Biol. 2008 Jun 27;9(Suppl 1):S2. doi: 10.1186/gb-2008-9-s1-s2 (PMC2447536; doi:10.1186/gb-2008-9-s1-s2)
Supplement: Additional data file 2 — Bar graphs of mean P20R values within each evaluation category [file gb-2008-9-s1-s2-S2.pdf]

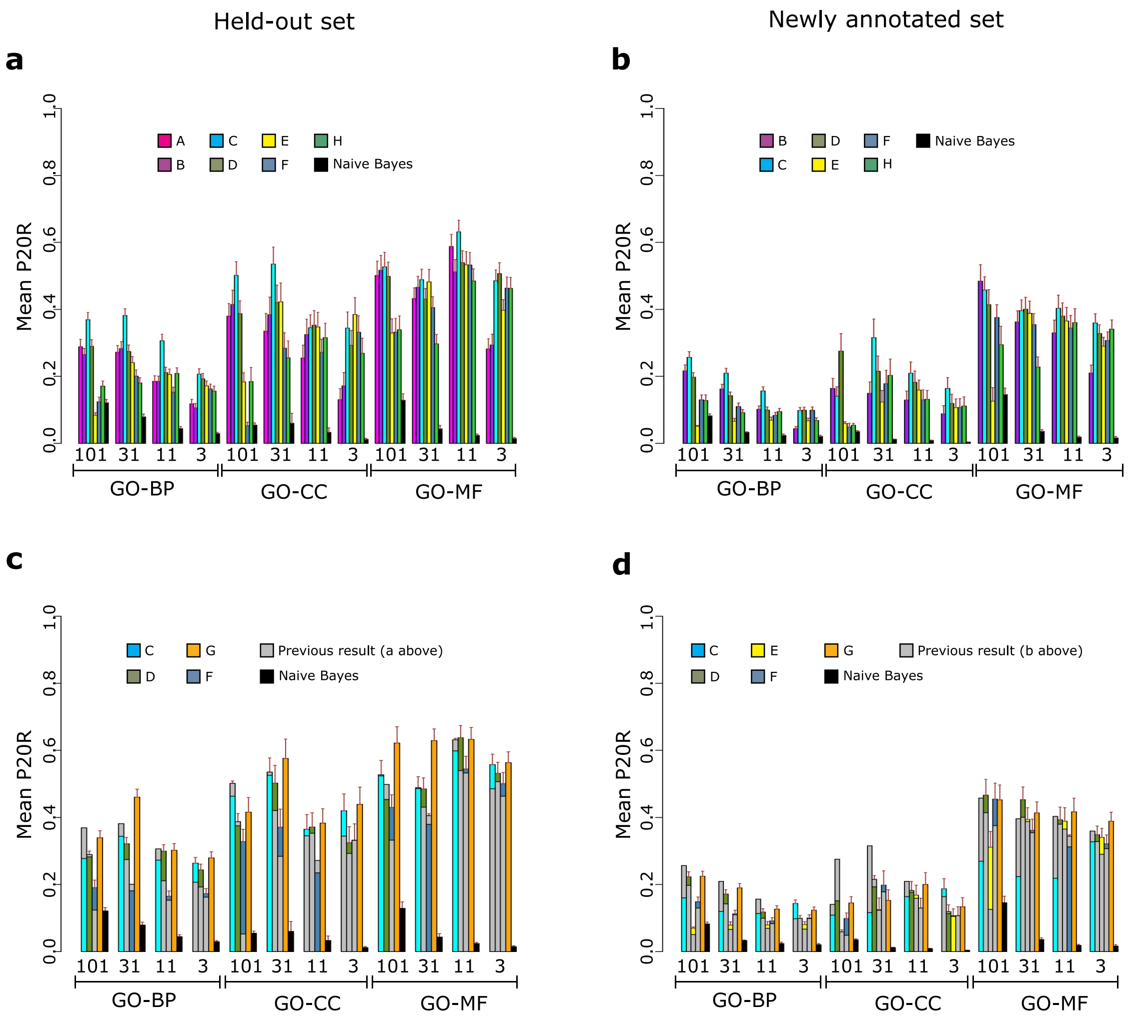

Figure S2: Mean precision values at 20% recall (P20R) within each evaluation category. (**a**) Evaluation of initial submissions using held-out genes. Biological Process (GO-BP), Cellular Component (GO-CC), and Molecular Function (GO-MF) branches are indicated on the x-axis, grouped by specificity (indicated by the minimum number of genes in the training set associated with each GO term in a given category). (**b**) Mean P20R within each evaluation category, evaluated prospectively using newly-annotated genes. (**c**) (**d**) As described in (a),(b), with colored region indicating performance of second-round submissions and grey region indicating performance in the first set of submissions, for comparison.
