# Supplementary material for: A critical assessment of Mus musculus gene function prediction using integrated genomic evidence
Source: Genome Biol. 2008 Jun 27;9(Suppl 1):S2. doi: 10.1186/gb-2008-9-s1-s2 (PMC2447536; doi:10.1186/gb-2008-9-s1-s2)
Supplement: Additional data file 3 — Bar graphs comparing properties of GO annotations in the held-out gene set, in the newly annotated gene set and in the training set. [file gb-2008-9-s1-s2-S3.pdf]

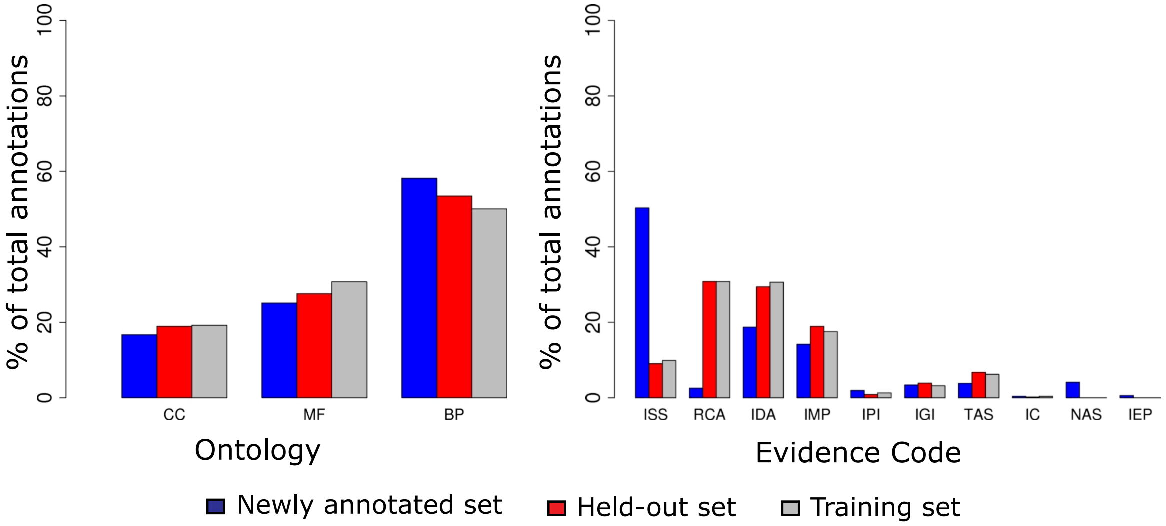

Figure S3: Properties of GO annotations used in evaluation based on the held-out gene set as compared with annotations used in the prospective evaluation. Distribution of GO term annotations among the three GO branches (left panel) and distribution of types of supporting evidence (right panel). Each panel shows GO annotations from the newly annotated set (blue), the held-out set (red), and the training set (grey).
