# Supplementary material for: A critical assessment of Mus musculus gene function prediction using integrated genomic evidence
Source: Genome Biol. 2008 Jun 27;9(Suppl 1):S2. doi: 10.1186/gb-2008-9-s1-s2 (PMC2447536; doi:10.1186/gb-2008-9-s1-s2)
Supplement: Additional data file 4 — Clustergram indicating Pearson correlation coefficients of the P20R performance measure among different submissions. [file gb-2008-9-s1-s2-S4.pdf]

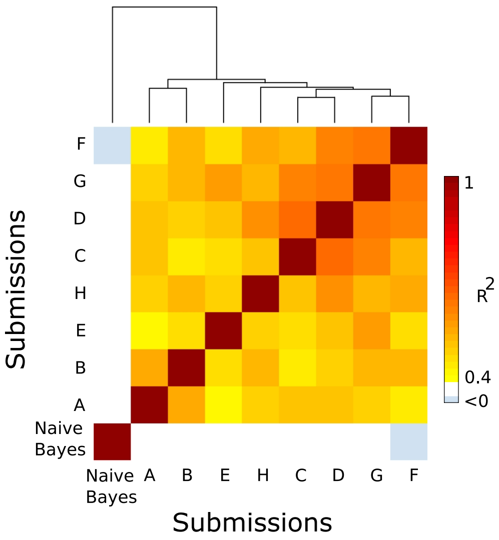

Figure S4: Correlation of the P20R performance measure among different submissions, grouped by complete linkage hierarchical clustering. The heatmap indicates Pearson correlation coefficient between pairs submissions.
