# Supplementary material for: A critical assessment of Mus musculus gene function prediction using integrated genomic evidence
Source: Genome Biol. 2008 Jun 27;9(Suppl 1):S2. doi: 10.1186/gb-2008-9-s1-s2 (PMC2447536; doi:10.1186/gb-2008-9-s1-s2)
Supplement: Additional data file 5 — Heatmaps of precision at several recall values evaluated using held-out annotations on all GO terms within each of the 12 evaluation categories for each submission. [file gb-2008-9-s1-s2-S5.pdf]

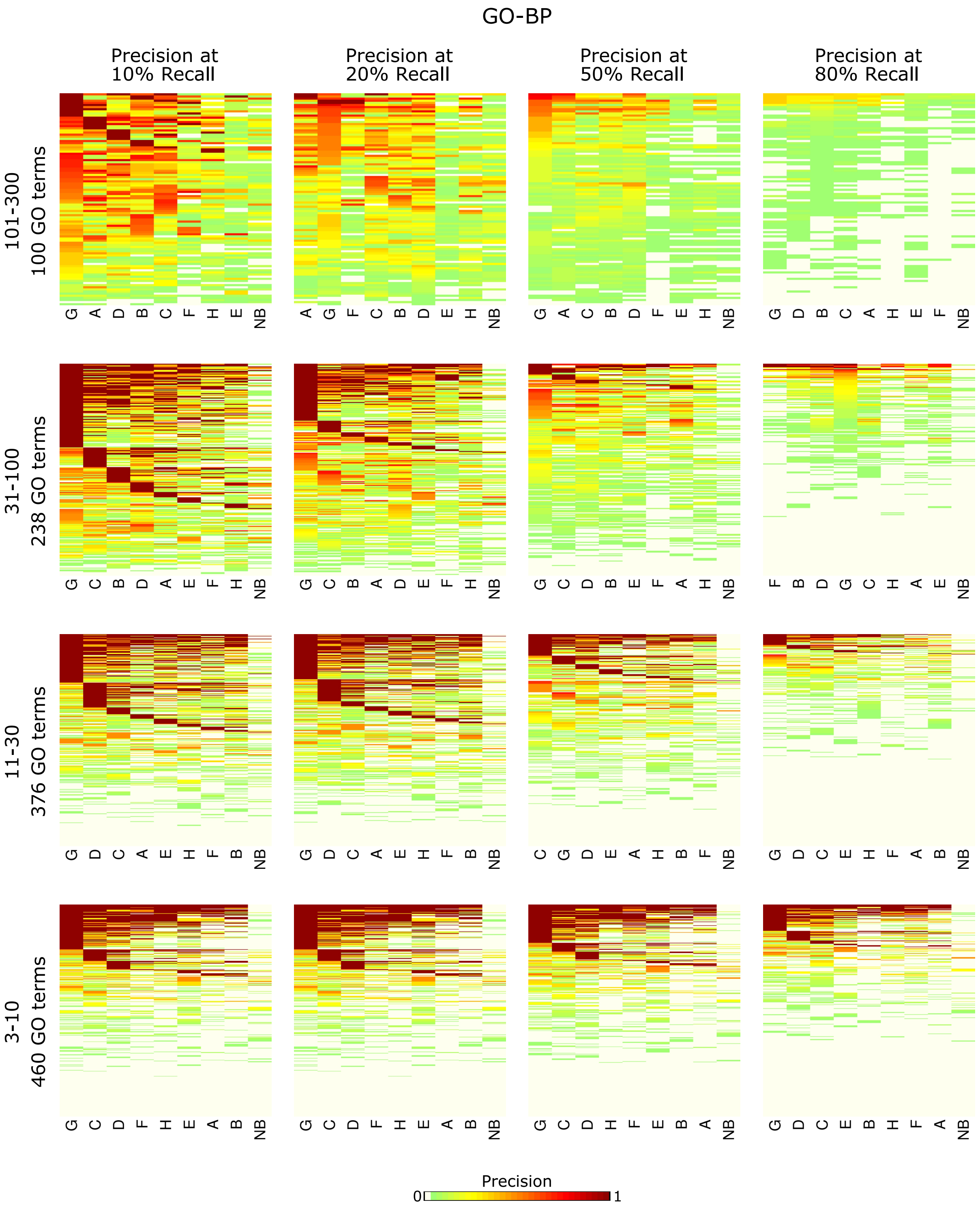
GO-BP
Precision at 10% Recall
Precision at 20% Recall
Precision at 50% Recall
Precision at 80% Recall
101-300 100 GO terms
31-100 238 GO terms
11-30 376 GO terms
3-10 460 GO terms
G A D B C F H E NB
A G F C B D E H NB
G A C B D F E H NB
G D B C A H E F NB
G C B D A E F H NB
G C B A D E F H NB
G C D B E F A H NB
F B D G C H A E NB
G D C A E H F B NB
G D C A E H F B NB
C G D E A H B F NB
G D C E H F A B NB
G C D F H E A B NB
G C D F H E A B NB
G C D H F E B A NB
G D C E B H F A NB
Precision
0
1

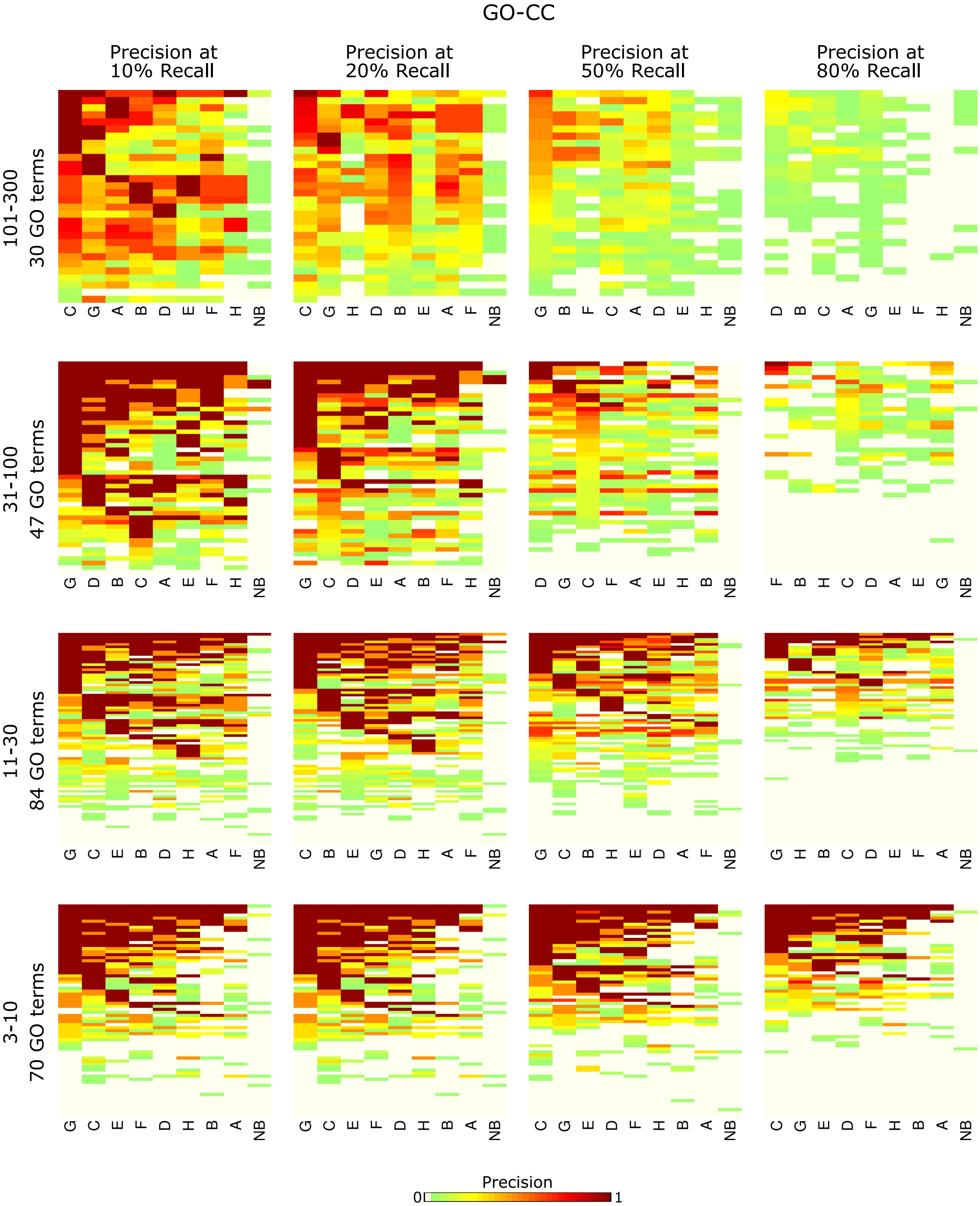
GO-CC
Precision at 10% Recall
Precision at 20% Recall
Precision at 50% Recall
Precision at 80% Recall
101-300 30 GO terms
31-100 47 GO terms
11-30 84 GO terms
3-10 70 GO terms
C G A B D E F H NB
C G H D B E A F NB
G B F C A D E H NB
D B C A G E F H NB
G D B C A E F H NB
G C D E A B F H NB
D G C F A E H B NB
F B H C D A E G NB
G C E B D H A F NB
C B E G D H A F NB
G C B H E D A F NB
G H B C D E F A NB
G C E F D H B A NB
G C E F D H B A NB
C G E D F H B A NB
C G E D F H B A NB
Precision
0
1

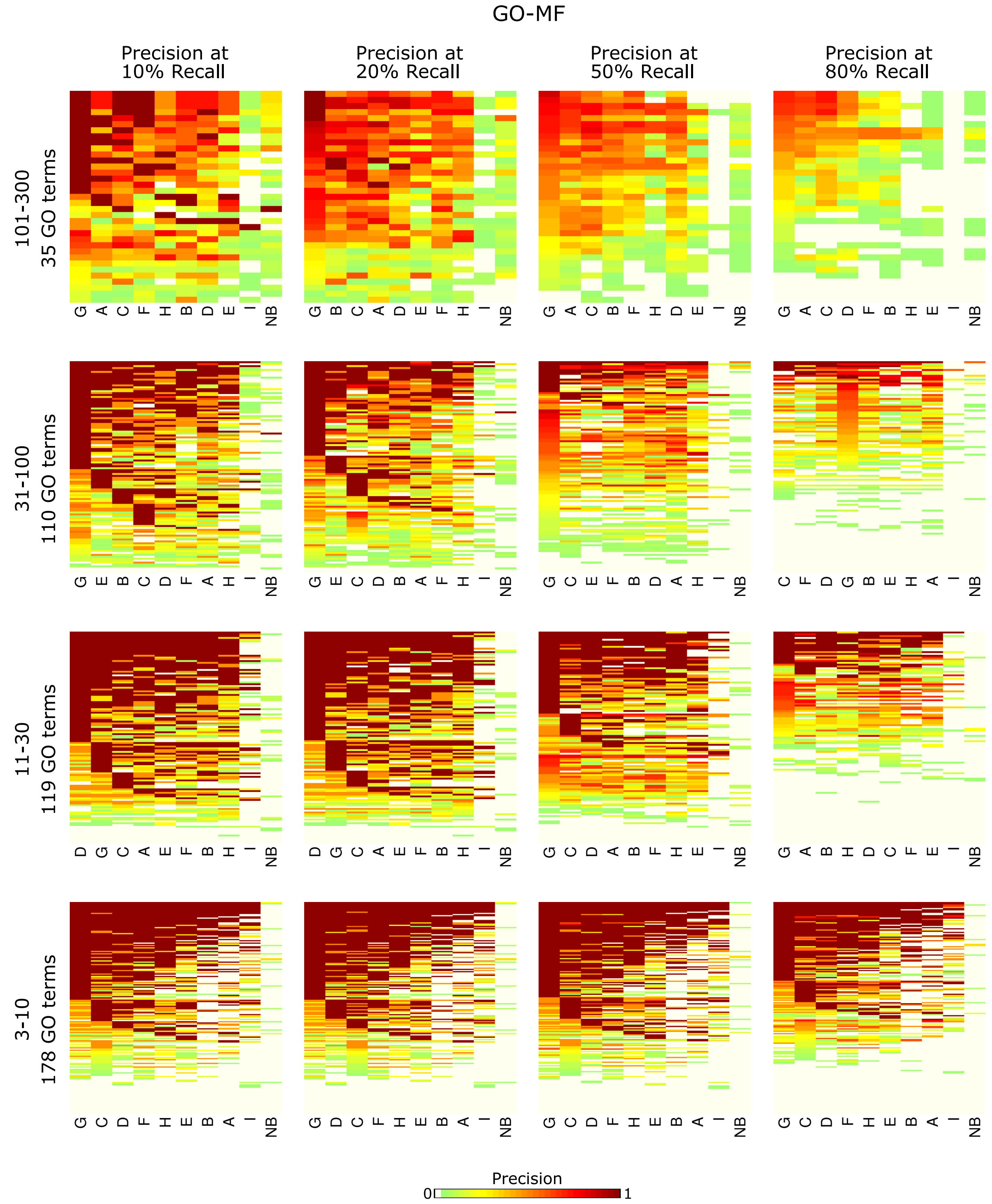

Figure S5: Precision at several recall values (10%, 20%, 50% and 80%) evaluated using held-out annotations on all GO terms (corresponding to rows) within each of the 12 evaluation categories for each submission (corresponding to columns). The heatmap for each evaluation category reflects the precision value for each submission / GO term combination at a fixed recall value (indicated at top). Within each heatmap, submissions (columns) were ordered according to the number of GO terms for which that submission achieved a precision (at the fixed recall value indicated at top) above the average of M, where M is the set of maximum precision values achieved by each submission within that evaluation category at the indicated recall value.
