# Supplementary material for: A critical assessment of Mus musculus gene function prediction using integrated genomic evidence
Source: Genome Biol. 2008 Jun 27;9(Suppl 1):S2. doi: 10.1186/gb-2008-9-s1-s2 (PMC2447536; doi:10.1186/gb-2008-9-s1-s2)
Supplement: Additional data file 6 — Heatmap of median precision at several recall values evaluated using held-out annotations within each of the 12 evaluation categories per submission [file gb-2008-9-s1-s2-S6.pdf]

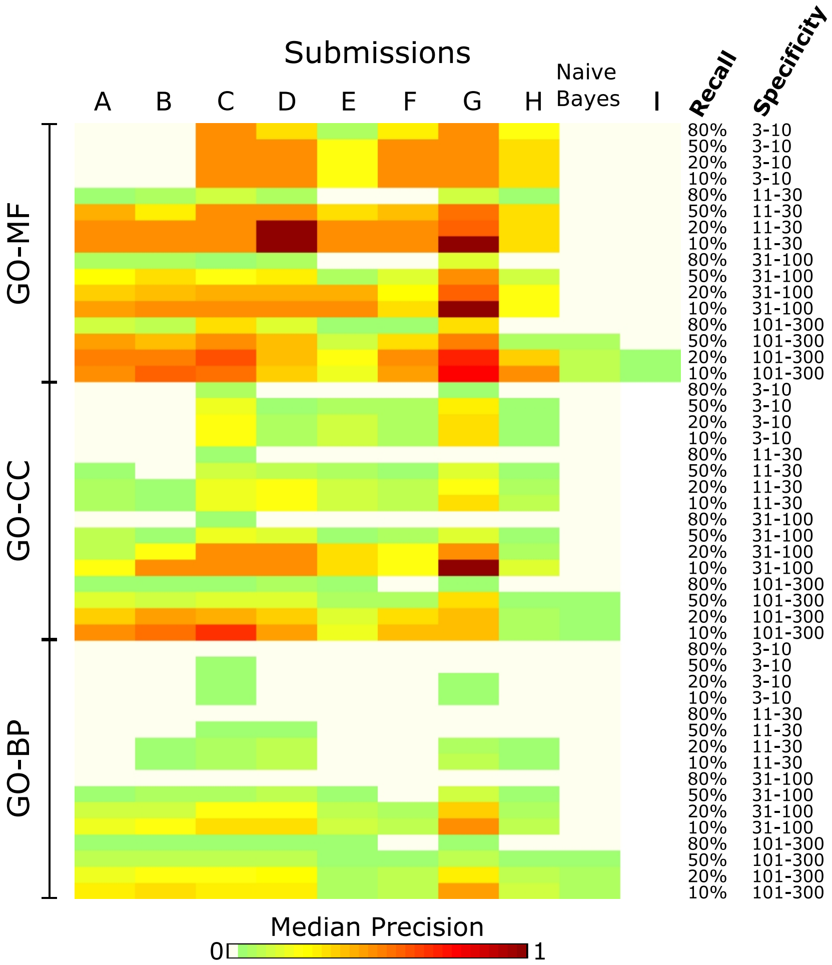

Figure S6: Median precision at several recall values (10%, 20%, 50% and 80%) evaluated using held-out annotations within each of the 12 evaluation categories (rows) for each submission (columns).
