# Supplementary material for: A critical assessment of Mus musculus gene function prediction using integrated genomic evidence
Source: Genome Biol. 2008 Jun 27;9(Suppl 1):S2. doi: 10.1186/gb-2008-9-s1-s2 (PMC2447536; doi:10.1186/gb-2008-9-s1-s2)
Supplement: Additional data file 20 — Description of the function prediction method used in each submission. [file gb-2008-9-s1-s2-S20.pdf]

Box 1. Brief Description of Methods used by Team "A"

Guillaume Obozinski, Charles Grant, Jian Qiu, Gert Lanckriet, Michael I. Jordan and William Stafford Noble

Calibrated ensembles of SVMs

This method consists of three phases: kernel construction, SVM training and calibration.

We begin by computing two kernels -- linear and Gaussian -- for each data type. In addition, we compute several data-specific kernels: for protein-protein interaction data, we use diffusion kernels; for gene expression data, we use a linear kernel calculated over a base linear kernel matrix (i.e., we square the kernel matrix).

Next, for each GO term, we compute a series of data set-specific SVMs. A gene is considered a positive example for a GO term T if it is annotated with any descendant of T in the ontology. The gene is considered a negative example of GO term T if it is not a positive example and if it is not annotated with any ancestor of T. For each combination of a GO term annotation and an individual kernel, several SVMs are trained using a cross-validation scheme. We skip GO terms for which fewer than five training examples are available.

In the final stage, we process the pooled validation data via logistic regression to calibrate all kernel-specific SVM outputs into a global score. In this stage, we must handle missing data, because most genes have one or more missing data types. However, only a few different patterns of missing data types (between 10 and 12 depending on the ontology) cover most of the genes, and the remaining patterns are very close in Hamming distance to one of them. We therefore learn several logistic regressions per GO term, each corresponding to one of these most frequent patterns of missing data types. For a given gene, we use the logistic regression whose pattern of missing data most closely matches the observed pattern, imputing the remaining missing entries (via the mean) when necessary.

Box 2. Brief Description of Methods used by Team "B"

Hyunju Lee, Minghua Deng, Ting Chen and Fengzhu Sun

An Integrated Kernel-Logistic Regression Method for Protein Function Prediction. The kernel logistic regression (KLR) method combines a kernel function with the logistic regression. For a given network, assume that the kernel $K(i, j)$ defines the similarity between two proteins i and j. Given a function of interest, the logistic regression for the probability that protein i has the function is modeled as

$$\log \frac{\Pr(X_i = 1)}{1 - \Pr(X_i = 1)} = \gamma + \delta M_0(i) + \eta M_1(i), \tag{1}$$

where $X_i = 1$ indicates that the protein i has the function, and γ, δ, and η are the parameters to be determined from the known proteins and their interactions. $M_0(i)$ is the sum of kernel similarity measures between protein i and other known proteins that do not have the function of interest, and $M_1(i)$ is similarly defined as that for protein i and other known proteins that have the function of interest.

$$M_0(i) = \sum_{j \neq i,\ x_j \text{ known}} K(i, j) I\{x_j = 0\},$$
$$M_1(i) = \sum_{j \neq i,\ x_j \text{ known}} K(i, j) I\{x_j = 1\}.$$

This model is extended to incorporate multiple data sets. Details are described in the supplementary materials.

Different kernels were used for different types of data sets. For the protein interaction data, we used an adjacency kernel where the similarity measure is 1 if two proteins interact, and 0 otherwise. For Pfam and Interpro protein-domain data, we defined the domain information for protein i as a vector $\mathbf{v_i}$. If the protein has a given domain, the value of the domain in the vector is 1, and 0 otherwise. Then, we defined the following polynomial kernel based on protein-domain data:

$$K_n(i, j) = (1 + \mathbf{v_i}\mathbf{v_j})^n \tag{2}$$

We treated MGI phenotype ontology, Biomart and Inparanoid phylogenetic profiles, and OMIM disease data sets similarly as the protein-domain data.

For the gene expression data, we calculated the Pearson correlation coefficient (PCC) between the expression profiles of two proteins i and j, and the kernel function is defined as

$$K_n(i,j) = (1 + \mathrm{PCC}(i,j))^n \tag{3}$$

We integrate all these data sets for each protein functional category using a heuristic method for feature selection. We first estimated the importance of each data source based on the AUC score using five-fold cross-validation. We then integrated up to three data sources with the highest AUC scores and that the AUC scores are above 0.65 using the following formula.

$$\log \frac{\Pr(X_i = 1)}{1 - \Pr(X_i = 1)} = \gamma + \sum_{k=1}^{3} \{\delta_k M_0^k(i) + \eta_k M_1^k(i)\}. \tag{4}$$

Box 3. Brief Description of Methods used by Team "C"

Sara Mostafavi, David Warde-Farley, Chris Grouios, Debajyoti Ray and Quaid Morris

GeneMANIA

GeneMANIA has three stages: 1) generating interaction networks (or *affinity networks*) from each dataset, 2) combining the affinity networks to create a process-specific composite network, and 3) doing label propagation in the composite network.

1) Generating affinity networks: Each dataset corresponds to a continuous or binary-valued matrix whose rows are gene profiles across features represented by columns. First, binary-valued matrices are transformed to up-weight rare features by replacing "1"s with log(frequency of "1" in the column). Then all data matrices are converted into a network in which each node is a gene and whose edge weights are the Pearson correlation coefficients of the corresponding gene profiles. To sparsify the resulting networks, we zero any pair-wise similarity that is not among the top 50 highest similarities for any genes.

2) Process-specific network combination: For each Gene Ontology category, we build a process-specific composite network whose edge weights are a weighted linear sum of the corresponding edge weights in each individual network. The weights in the linear sum are positive values assigned to each network that estimate its relevancy for predicting membership in the given category. We compute these network weights using Bayesian linear regression.

3) Label propagation to predict gene function: We predict gene function using an efficient label propagation algorithm based on a Gaussian random field interpretation of the affinity network (Zhu et al, 2003 and Zhou et al 2004). Given a list of positive and negative genes and an affinity network, this algorithm assigns a discriminant value to each gene in the network which can be thresholded to classify genes. Our variation of the label-propagation algorithm introduces a 'label bias' to account for *a prior* expectation of number of annotated genes in a given functional class; this results in a significant improvement over previous network-based function prediction algorithms.

Box 4. Brief Description of Methods used by Team "D"

Yuanfang Guan, Chad L Myers, Zafer Barutcuoglu, and Olga G Troyanskaya

Multi-label hierarchical classification and Bayesian integration of diverse data sources

We developed a classification algorithm that leverages both the heterogeneity across data sources and hierarchical relationships among GO terms. The basis of our approach is the support vector machine (SVM) classifier, which has been used successfully for supervised learning on high-dimensional data in a number of different application domains.

Integrating data heterogeneity: Biological data are heterogeneous in both their reliability or quality as well as the biological phenomena they are able to capture. To account for this heterogeneity, we trained individual GO term classifiers for each dataset and used a Bayesian model to form an ensemble classification. The parameters for ensemble classifier were learned through bootstrapping on the individual datasets, and either results from the Bayesian framework or a single classifier were chosen based on held-out data. We gained a 12 percent improvement compared to a single classifier on a combined feature set (across all datasets) by such an approach.

Leveraging the GO hierarchy: Furthermore, we have previously observed that SVM predictions can be improved by leveraging the hierarchical relationships among GO terms. Thus, we systematically split each GO term branch (BP, CC and MF) into multiple sub-graphs. Based on their classification accuracy assessed on held-out data, the predictions of individual GO terms are combined using Bayesian integration, incorporating information about hierarchical relationships among the GO terms from each local graph. Essentially, this method allows classifiers in different parts of the GO hierarchy to influence the prediction of related classes (i.e. ancestor or descendant nodes) in a way that reflects their relative accuracy. In our complete method, both the dataset ensemble classifier and hierarchical correction were applied, which results in an overall improvement of 18 percent over a single classifier on a combined feature set.

Box 5. Brief Description of Methods used by Team "E"

Chase Krumpelman, Wan Kyu Kim and Edward Marcotte

Combination of classifier ensemble and gene network

Our annotation prediction approach combined two different strategies: a network-based approach and a feature-based classification approach. The network approach learned functional associations between genes based on their likelihood of sharing GO annotation, then propagated GO annotation of known genes to unannotated genes. The feature-based approach employed an ensemble of classifiers to predict GO annotations for genes directly from the data.

The feature-based approach captured strong associations between particular features and GO terms, e.g. a protein domain indicating a specific enzymatic activity. We trained three classifiers - naïve Bayes, decision tree, and boosted tree - for each GO annotation then combined results from the three by naïve Bayes to predict which genes receive that annotation. The network approach was complementary and effectively learned functions shared between genes, as captured by similarities in the genes' mRNA co-expression profiles, interactions, and other evidence for functional relationships. Each approach provided the probability of a given gene having a given GO annotation. The final prediction score was the maximum of the two independent predictions for a particular gene and annotation.

In all three GO hierarchies, our results displayed a trend of higher accuracy on infrequent terms and lower accuracy on frequent terms. Between the hierarchies, the relative accuracy was higher for BP and CC terms than for MF terms.

Due to the running time of our network construction algorithm, our initial prediction for the newly annotated genes was based only on the feature-based approach, yielding an accuracy no better than the naïve Bayes “straw man” (Figure 1b, group E). We saw considerable improvement when the network results were used in combination with the feature-based results (Figure 2b, group E*). Thus, combining heterogeneous methods shows promise for gene function prediction, much as has been shown in other fields, such as 3-D structure prediction and gene finding.

Box 6. Brief Description of Methods used by Team "F"

Trupti Joshi, Chao Zhang, Guan Ning Lin, and Dong Xu

GeneFAS

GeneFAS (Gene Function Annotation System) [1, 2] utilizes various types of biological data including microarray, SAGE, inparanoid, phylogenetic profiling, protein-protein interactions and sequence-domain information for gene function prediction. Based on index level comparison of Gene Ontology annotations for defining functional similarities, GeneFAS quantifies the relationship between functional similarity and biological data and codes the relationship into a 'functional linkage graph', where each node represents one gene and the weight of each edge is characterized by the Bayesian probability of function similarity between two genes. GeneFAS then uses Boltzmann machine to characterize the global stochastic behavior of the functional linkage graph and perform global function prediction. In the Boltzmann machine, we consider the system going through a dynamic process from non-equilibrium to equilibrium, which corresponds to the optimization process for function prediction. To achieve the global optimization, we apply a simulated annealing technique where the initial state of all unannotated genes (nodes) is set to 0 or 1 randomly. The state of any annotated gene is always 1. If an unannotated gene is assigned with the state 1, its function will be predicted based on its immediate neighbors with known functions, using a guilt-by-association method. Next, starting with a high temperature, GeneFAS picks a node i and computes the probability Pi, then updates its state to 1 if the probability Pi is above a certain threshold. Each update of function prediction is based on its immediate neighbors with state 1 (i.e. known functions or predicted functions in previous steps) using the guilt-by-association method. The iterations are done till all the nodes in the graph reach the equilibrium. GeneFAS has been developed in both a GUI and a command-line version. It is available at http://digbio.missouri.edu/software/genefas/.

1. Joshi, T., Chen, Y., Becker, J.M., Alexandrov, N. & Xu, D. Genome-scale gene function prediction using multiple sources of high-throughput data in yeast Saccharomyces cerevisiae. *OMICS* **8**, 322-333 (2004).

2. Chen, Y & Xu, D. Global Protein Function Annotation through Mining Genome-Scale Data in Yeast Saccharomyces cerevisiae. *Nucleic Acid Research*. 32, 6414-6424 (2004).

Box 7. Brief Description of Methods used by Team "G"

Weidong Tian*, Murat Tasan*, Francis D. Gibbons and Frederick P. Roth
(*contributed equally)

Funckenstein

We combined scores from 'guilt by profiling' and 'guilt by association' methods.

Guilt-by-profiling predictions exploited gene-specific 'profiles' of binary properties (e.g. protein sequence pattern matches, association with a particular phenotype, or membership in an expression or phylogenetic cluster). For each GO term, we constructed a random forest ensemble classifier, with genes as training examples and profile variables as candidate predictors. Individual decision trees in the forest were constructed using bootstrap sampling from examples, with sampling of candidate predictors at each internal node. Trees then vote on annotation of a gene with the GO term of interest, with the proportion of positive votes as a probability measure.

Guilt-by-association predictions relied on a vector of 26 binary relationships characterized for each gene pair (e.g., protein interaction, or expression correlation above a threshold). Training gene-pairs were considered functionally linked if they shared any specific GO term from the category of interest. For each of the 12 GO term categories, a decision tree was trained to predict 'functional linkage'. The resulting 12 functional linkage graphs—with edge weights representing the probability that the corresponding genes are functionally linked—were used to predict GO terms in the corresponding 12 term categories. For each gene A and GO term X, the top three edge weights among all gene pairs (A, B), where B is the set of genes currently annotated with GO term X, were averaged and returned as a prediction score.

Random forest and functional linkage scores were combined using a logistic regression model with a single relative weighting parameter. This parameter was chosen separately for each of the 12 GO categories to maximize average area under the precision-recall curve.

See Supplementary Information for further details.

Box 8. Brief Description of Methods used by Team "H"

Yanjun Qi, Judith Klein-Seetharaman and Ziv Bar-Joseph

Protein Function Prediction Using ‘Query Retrieval’ Methods

We used a ‘Query Retrieval’ method to predict membership of a given protein in a specific gene ontology (GO) category. The features used by our method are vectors of pairwise similarity values derived from high throughput biological data sources. Given a specific GO category A we divide protein-protein pairs into two classes: positive pairs (both proteins belong to A) and negative pairs (one of the proteins belongs to A while the other does not). Negative examples are randomly selected from all proteins not assigned to category A. The positive and negative pairs are used to train a random forest (RF) classifier [2]. Given a test gene, we derive pairwise features between that gene and all genes assigned to A and use the RF model to classify the new pairs. If most pairs containing the test gene and a positive gene are classified as positive we assign that gene to A. The randomization and ensemble strategies within the RF classifier make it robust to noisy and/or missing features, which are often found in large biological datasets [1].

The underlying assumption of our method is that proteins assigned to a certain functional category are similar to each other with respect to the collected data (features). For categories containing less than thirty genes our assumption is one of the very few reasonable frameworks for a learning strategy. Indeed, our method performs very well when applied to these small functional categories (we place in the top 3 for almost all such categories). These categories are arguably the hardest to predict and in many cases contain a more detailed description for proteins, making them more useful for researchers. Since most GO categories are of small size we believe that our 'Query Retrieval' strategy is useful for many functional prediction tasks.

References

[1] L. Breiman, Random forests. Machine Learning, 45:5-32, 2001.
[2] Y. Qi, Z. Bar-Joseph, J. Klein-Seetharaman,"Evaluation of different biological data and computational classification methods for use in protein interaction prediction", Proteins: Structure, Function, and Bioinformatics. 63(3):490-500, 2006.

Box 9. Brief Description of Methods used by Team "I"

M.Leone and A.Pagnani

Function prediction with message passing algorithms

In this work we integrated three sources of data: the Protein-Protein Interaction (PPI) graph, a graph inferred by Gene Arrays experiment (GA) (Zhang and Su dataset), and the graph inferred by Protein Domain data (PD). Given the large number of GO terms, we decided to prune backward each annotation, keeping only the most specific term, *i.e.* the leaves in the GO graph.

Let G be our graph, $V=\{1,\dots,N\}$ the set of proteins, $F=\{1,\dots,nF\}$ the set of annotations. Each protein i belonging to V can then be characterized via a discrete variable X_i that can take values $f \in F$. One would like to compute the probability $P_i(f)=Pr(X_i=f)$ for each protein to have a given function f given the functions assigned to the proteins in the rest of the graph.

Given sets V, A and $V \backslash A$, the interactions G, the graph of unclassified proteins $U \subset G$ and the set of observed function F we define a score:

$$E[X_1,\dots,X_N] = \sum_{i<j} J_{i,j}\delta(X_i;X_j) - \sum_i h_i(X_i)$$

$J_{i,j}$ being the adjacency matrix of U ($J_{i,j}=1$ if i and $j \in V \backslash A$ and they interact with each other). $\delta(X_i;X_j)=1$ when $X_i=X_j$ and zero otherwise, and $h_i(X_i)$ is an external field counting the number of classified neighbors of protein i in the original graph G that have at least function X_i. From the Gibbs potential one can compute the free-energy of the system:

$$F = \frac{1}{\beta N}\log\{\sum_{\{x_i\}}\exp(-\beta E[x_1,\dots,x_n])\}$$

Configurations with larger statistical weights are those maximizing the free-energy via a message-passing algorithm (see Materials), under the assumption that correlations are low enough in the graph, *i.e.* $P_{ij}(X_i,X_j) \propto P_i(X_i)P_j(X_j)$ for a random choice of i,j. We can calculate $P_j(X_j)$ as product of conditional probability contributions incoming to j from all neighboring proteins conditioned to the fact that j has function X_j.
