# Supplementary material for: A critical assessment of Mus musculus gene function prediction using integrated genomic evidence
Source: Genome Biol. 2008 Jun 27;9(Suppl 1):S2. doi: 10.1186/gb-2008-9-s1-s2 (PMC2447536; doi:10.1186/gb-2008-9-s1-s2)
Supplement: Additional data file 21 — Detailed description of the submission methods and the straw man classifier. [file gb-2008-9-s1-s2-S21.pdf]

Supplementary Methods for Submission A

Predicting mouse gene function with calibrated ensembles of support vector machines: — *Methods* —

Guillaume Obozinski
Department of Statistics
University of California, Berkeley

Charles Grant
Department of Genome Sciences
University of Washington

Jian Qiu
Department of Genome Sciences
University of Washington

Gert Lanckriet
Department of Electrical and Computer Engineering
University of California, San Diego

Michael I. Jordan
Department of Statistics
Department of Computer Science
University of California, Berkeley

William Stafford Noble
Department of Genome Sciences
Department of Computer Science and Engineering
University of Washington

October 20, 2006

The data and labels are provided as a collection of matrices, with rows corresponding to genes and columns corresponding to features. Our strategy for making function predictions from this data consists of four stages: (1) computing kernel matrices, (2) training a large collection of GO-term-specific SVMs, (3) mapping vectors of SVM discriminants to probabilities, and (4) handling missing data by choosing at test time a relevant combination of kernels. These four stages are detailed next.

	Name	Linear	Normalized Linear	LoL	Normalized LoL	RBF	Diffusion
1	MGI	✓	✓				
2	OMIM	✓	✓			✓	
3	Inpar	✓	✓				
4	Biomart	✓	✓				
5	Inter	✓	✓			✓	
6	PfamA	✓	✓			✓	
7	PPI	✓	✓				✓✓✓
8	Su	✓	✓			✓	
9	Zhang	✓	✓	✓	✓	✓	
10	SAGE	✓	✓			✓	

Table 1: **Various types of kernels are computed for each data set.** Details of the kernel computations are provided in the text. "LoL" is "linear on linear."

1 Kernels

Our first challenge is to choose an appropriate representation of each data type. In the context of SVM learning, this choice is embodied in the choice of kernel function. Our strategy is to construct several kinds of kernels for each data matrix. The first kind of kernel is linear: $K(x,y)=x \cdot y$. Prior to computing this kernel, the data is standardized; i.e, each variable is centered to have mean 0 and scaled to have variance 1. We compute two linear kernels, with and without normalization, where the normalized version $\tilde{K}$ of kernel K is $\tilde{K}(x,y) = K(x,y)/\sqrt{K(x,x)K(y,y)}$. We also compute one or more non-linear kernels for each data set, as shown in Table 1. The radial basis function (RBF) kernel is defined as $K(x,y) = e^{-\|x-y\|^2}$. The other dataset-specific kernels are described below.

For the protein-protein interaction data, we compute a diffusion kernel [Kondor and Lafferty, 2002] on the binary adjacency matrix, in which vertices are the proteins and edges are interactions. Diffusion kernels correspond to embeddings of the vertices in a Hilbert space whose inner product between two vertices equals the probability of getting from one vertex to the other in time τ if we perform a random walk on the graph. The method therefore efficiently accounts for all possible paths connecting two nodes, and for the lengths of those paths. Nodes that are connected by shorter paths contribute more to the similarity. The diffusion computation involves a diffusion parameter τ that controls the relative weighting of long versus short paths. We compute three diffusion kernels with $\tau \in \{0.1, 1, 10\}$.

For the Sage expression data we use only the sum of all tag counts per gene. For the phylogenetic profiles, we use only the binary data.

For the Zhang expression data, Zhang et al. conclude from their study that patterns of co-expression accross tissues are more predictive of function than tissue-specific expression levels. Given that we have a lot of unlabelled data, this suggests that a representation of a gene in terms of co-expressed genes might be relevant. In a linear kernel matrix built from expression data, the columns corresponding to a gene can be interpreted as a representation of the gene in terms of its co-expressed genes. If we consider the latter matrix as a new representation of the data, we can again build a kernel from it. We choose to build a linear kernel from that matrix which is equivalent to squaring the original kernel matrix. Note that

this is different from the quadratic kernel, which is defined as the Hadamard product of the kernel matrix with itself and not the regular matrix product.

2 Term-specific support vector machines

In the second stage, we train a collection of SVM classifiers for each GO term. Thus, the output of this stage is a matrix with three dimensions: gene, GO term and kernel-specific SVM. Note that typically not all data types are available for a given gene, so that some of the entries in this matrix are missing. We handle these missing values in the subsequent stages.

In order to train each (binary) SVM classifier with respect to a given GO term T, we partition the set of mouse genes into three sets. First, all genes that are annotated with T are labelled as "positive". Genes that don't have any annotation in a GO hierarchy are assigned to the root of the hierarchy. Next, we traverse from T along all paths to the root of the Gene Ontology graph. At each GO term along this path, we look for proteins that are assigned to that term and not to any of that term's children. We consider that such proteins might be properly assigned to T, and so we label those proteins as "uncertain" and do not use them during training. Finally, all proteins that are not on the path from T to the root are labelled as "negative." For efficiency, for the purpose of learning the SVMs, we then randomly select a subset of the negative examples, so that the ratio of negatives to positives is at most 3-to-1, down to a minimum of 50 negative examples.

We train one SVM per kernel and per GO term using five-fold cross-validation. The SVM we train uses two soft-margin parameters C^+ and C^-, corresponding to penalizing respectively false positives and false negatives, which are chosen proportionally to the inverse of the number of positives and negatives respectively. More precisely $C^+ = \frac{0.01}{\sharp\text{positives}}$ and $C^- = \frac{0.01}{\sharp\text{negatives}}$; these values were chosen empirically. The discriminant function used on any new gene is the average of the five discriminants learnt.

3 Mapping to probabilities

For each GO term and each kernel constructed, the output of a trained SVM on a new gene is a discriminant value, which is proportional to the distance of that gene to the separating hyperplane. This number is not comparable from one SVM to another and moreover, a discriminant value cannot be interpreted directly as an absolute confidence measure. Therefore, one needs to *calibrate* this value, i.e., map it to a probability that reflects the degree of confidence that we have when assigning that gene to the GO term corresponding to the SVM built. A simple calibration method just fits a logistic regression to the non-calibrated values using held-out data [Platt, 1999]. One possibility for us would be to calibrate the discriminant function of each SVM separately and then combine the calibrated values. However, we choose to combine directly all the discriminant functions of SVMs learnt from a set of kernels with a plain logistic regression with vector input. The algorithm we use to fit the regression is the classical iterative re-weighted least squares (IRLS) method.

4 Handling missing data

There are essentially two ways in which the lack or absence of data impacts our approach. First for a certain gene, labelled or not, only a subset of the data types are available, which is a priori different for every gene. Second, for some GO terms we identify only a very small number of positive examples, which makes it impossible or highly unlikely to learn an SVM correctly. If we have less than five positive examples for a certain GO term, we give up trying to learn that term. As a consequence of both, each gene will only have discriminant values for a subset of the kernels that we could a priori use to learn a GO term.

This poses a challenge at the level of the calibration. To learn a logistic regression for a large set of kernels, we will be forced to use the small set of labelled examples that are present in all these kernels. Conversely, if we want to learn from a large set of examples we will have to give up a certain number of kernels. A similar problem appears on the side of the genes that we want to apply this logistic regression to. To a certain extend the problem can be partially addressed by *imputation*: if a couple of values are missing, we allow ourselves to fill them in with the average value of the corresponding discriminant function. Obviously it is not reasonable, either during training or prediction, to use imputation on a too large fraction of the missing data types.

We therefore proceed as follows. We call the "feature pattern" of a gene the list of data types available for that gene. For a certain GO term:

1. Eliminate from the set of usable data types the ones corresponding to SVMs that couldn't be learnt for lack of positive examples.

2. List all feature patterns present in the genes whose function is to be predicted.

3. Select the 10 most frequent ones.

4. Eliminate those feature patterns that are too rarely present in the training set (where "training set" is defined as the union of all the held-out sets obtained from learning the SVMs by five-fold cross-validation).

5. Add a backup pattern which comprises the inparanoid, Pfam and phylogenetic based kernels (common to a large fraction of the testing data) or based on the Su data if we aim at making predictions on the entire dataset.

6. Fix these patterns as models to learn, and fit each of the corresponding logistic regressions with all training points that have at least the data types needed for that logistic regression

7. If a test point pattern exactly matches the pattern of a model, then calibrate that test point with that model.

8. Assign the remaining testing points by identifying the model that matches most closely their pattern and use imputation to fill in the missing values. Abandon the few remaining test points for which no appropriate model is available.

5 Authors' contributions

GO and WSN designed the methodology and machine learning approach. GO wrote most of the code and ran some of the experiments. CG wrote some code and ran most of the experiments. JQ provided biological expertise, and GL provided machine learning expertise. MIJ and WSN supervised the research.

References

R. I. Kondor and J. Lafferty. Diffusion kernels on graphs and other discrete input spaces. In C. Sammut and A. Hoffmann, editors, *Proceedings of the International Conference on Machine Learning*. Morgan Kaufmann, 2002.

J. C. Platt. Probabilities for support vector machines. In A. Smola, P. Bartlett, B. Schölkopf, and D. Schuurmans, editors, *Advances in Large Margin Classifiers*, pages 61–74. MIT Press, 1999.

Author(s):

Hyunju Lee[1]

Minghua Deng[2]

Ting Chen[3]

Fengzhu Sun[3]

Institution(s):

[1] Department of Information and Communications, Gwangju Institute of Science and Technology, Gwangju, 500-712 Republic of Korea. [2] School of Mathematics, Beijing University, P. R. China. [3] Molecular and Computational Program, Department of Biological Sciences, University of Southern California, 1050 Childs way, Los Angeles, CA 90089-2910, USA.

Primary contact email:

tingchen@usc.edu, fsun@usc.edu

Methods:

Kernel logistic regression

The kernel logistic regression (KLR) method combines a kernel function with the logistic regression method [1]. For a given network, assume that the kernel $K(i, j)$ defines the similarity between two proteins i and j. Given a function of interest, the logistic regression for the probability that protein i has the function is defined as

$$\log \frac{\Pr(X_i = 1)}{1 - \Pr(X_i = 1)} = \gamma + \delta M_0(i) + \eta M_1(i), \tag{1}$$

where $X_i = 1$ indicates that the protein i has the function, and γ, δ, and η are the parameters to be determined from the known proteins and their interactions. $M_0(i)$ is the sum of kernel similarity measures between protein i and other known proteins that do not have the function

of interest, and $M_1(i)$ is similarly defined as that for protein i and other known proteins that have the function of interest.

$$M_0(i) = \sum_{j \neq i,\ x_j \text{ known}} K(i,j) I\{x_j = 0\},$$
$$M_1(i) = \sum_{j \neq i,\ x_j \text{ known}} K(i,j) I\{x_j = 1\}.$$

This model is extended to incorporate multiple data sets. Let $K^{(d)}(i,j)$ be the kernel matrix for the d-th data source. Then, the KLR model can then be further extended to

$$\log \frac{\Pr(X_i = 1)}{1 - \Pr(X_i = 1)} = \gamma + \sum_{d=1}^{D} \{\delta^{(d)} M_0^{(d)}(i) + \eta^{(d)} M_1^{(d)}(i)\}, \tag{2}$$

where

$$M_0^{(d)}(i) = \sum_{j \neq i,\ x_j \text{ known}} K^{(d)}(i,j) I\{X_j = 0\},$$
$$M_1^{(d)}(i) = \sum_{j \neq i,\ x_j \text{ known}} K^{(d)}(i,j) I\{X_j = 1\}.$$

$M_l^{(d)}(i)$ is the weighted number of neighbors of protein i having the function ($l = 1$) and not ($l = 0$) with weight $K^{(d)}(i,j)$ for protein j in the d-th network.

Kernels for the data sets

We used different kernels for different data sets. For the protein interaction data, we used an adjacency kernel where the similarity measure is 1 if two proteins interact, and 0 otherwise.

$$K(i,j) = \begin{cases} 1 & \text{if protein } i \text{ interacts with protein } j; \\ 0 & \text{otherwise.} \end{cases}$$

For Pfam and Interpro protein-domain data, we defined the domain information for protein i as a vector $\mathbf{v_i}$. If the protein has a given domain, the value of the domain in the vector is 1, and 0 otherwise. We defined the following polynomial kernel based on protein-domain data:

$$K_n(i,j) = (1 + \mathbf{v_i}\mathbf{v_j})^n \tag{3}$$

We tried this polynomial kernel with degree $n = 1$ and $n = 2$. We also tried the modified polynomial kernels with degree n=1 and n=2, which take into account the number of 1's in proteins i and j.

$$K_n(i,j) = (1 + \frac{\mathbf{v_i v_j}}{|\mathbf{v_i}| + |\mathbf{v_j}| - |\mathbf{v_i v_j}|})^n \quad (4)$$

We treated MGI phenotype ontology, Biomart and Inparanoid phylogenetic profiles, and OMIM disease data sets similarly as the protein-domain data. A protein in each data set is represented by a vector containing binary values for phenotypes, orthologues defined in different species or diseases. Then, we tried the polynomial kernels or the modified polynomial kernels.

For Zhang, Su, and Sage gene expression data, we calculated the Pearson correlation coefficient (PCC) between the expression profiles of two proteins i and j. Then, four kernels are implemented; thresholding the PCC with parameters 0.4 and 0.6, and the following kernel matrices (Eq. 5) with n=1 and n=2.

$$K_n(i,j) = (1 + \mathrm{PCC}(i,j))^n \quad (5)$$

Feature selection

Because the contributions of different data sources to protein functions can be different, we estimated the contributions of each data source based on a five-fold cross-validation (we used a two-fold cross-validation for GO terms with the number of genes ranging from 3 to 50 in the training set). Some data sources may not contain enough information to predict particular functions. For each function, instead of integrating all of the ten data sources, we chose a subset of the ten data sources and kernels for data integration. First, we calculated AUC scores with four different kernels (except for the protein interaction data set) and the best kernel was chosen for a given data source. Second, we integrated up to three data sources which have the highest AUC scores and that the scores are larger than 0.65 ROC scores.

Contributions:

HL developed and implemented methods of predicting protein functions. MD helped analyze the results. FS and TC directed this research.

References

[1] Lee, H., Tu, Z., Deng, M., Sun, F., and Chen, T. (2006) Diffusion Kernel-Based Logistic Regression Models for Protein Function Prediction. *OMICS: A Journal of Integrative Biology*, **10(1):** 40-55.

Supplementary Methods for Submission C

MouseFunc Methods

Sara Mostafavi[1], *David Warde-Farley*[1],
Chris Grouios[3], *Debajyoti Ray*[4], *Quaid Morris*[1,2,3]

METHODS

1. Algorithm

We predicted gene function using the guilt-by-association principle on an affinity network representation of MouseFunc data using a version of our unpublished GeneMANIA algorithm. We construct a separate affinity network for each dataset whose nodes are the genes and whose edges are weighted by gene similarity implied by the dataset. We compute weights for each affinity network separately for each function prediction task and use those weights to build a task-specific composite affinity network. To make predictions using the composite network, we use a variation of the Gaussian field label-propagation algorithm (Zhu et al 2003, and Zhou et al 2004).

First we describe how we make predictions given the single composite affinity network and then we will describe how we build the composite network by computing weights for the individual affinity networks.

1.1 GeneMANIA Label Propagation

To predict gene function we perform independent binary classification on each of the GO categories using a variation of the Gaussian random field label-propagation algorithm (Zhu et al 2003, Zhou et al 2004). Given a list of positive and negative genes and an affinity network described by a matrix, this algorithm assigns a discriminant value to each gene in the network. This value can then be thresholded to classify the genes as positives or negatives.

We represent an affinity network over n genes by an n by n symmetric matrix W in that each edge (i,j) in the affinity network corresponds to a non-zero, positive element $w_{i,j}$ of W. The value of $w_{i,j}$ represents the affinity (or similarity) of genes i and j implied by a particular dataset.

Given a binary classification task for which we have l labeled and u unlabeled genes ($l+u=n$), the Gaussian field algorithm computes a discriminant value $f_i \in [-1,+1]$ for each node i as follows:

$$f^\star = \mathtt{argmin}_f \sum_i (f_i - y_i)^2 + \sum_i \sum_j w_{ij}(f_i - f_j)^2$$

where y_i is set based on the label of node i. The obtained discriminant values satisfy the following equation:

$$f_i^\star = \frac{y_i + \sum_j w_{ij} f_j^\star}{1 + \sum_j w_{ij}}$$

If gene i has a positive label, y_i is set to one; if it has a negative label, y_i is set to -1; and if gene i is unlabeled y_i is set to k:

$$k = \frac{n^+ - n^-}{l}$$

[1]Computer Science Department and [2] Banting and Best Department of Medical Research, [3]Donnellly Centre for Celllular and Biomolecular Research, University of Toronto, Toronto, ON, Canada. [4]Gatsby Computational Neuroscience Unit, London, UK.

where n^+ is the number of positive examples and n^- is the number of negative examples. We have found that using the label bias k gives a significant improvement in the predictive accuracy of label propagation over setting $k=0$ as done in (Zhou et al 2004).

1.2 Combining Multiple Affinity Networks

GeneMANIA constructs a combined network W^{comb} by taking a weighted linear combination of all the individual affinity networks W^h, thus $W^{\mathtt{comb}} = \sum_h \alpha_h W^h$, where $\alpha_h \geq 0$. To determine the weight of each network we investigated several approaches. For our predictions in the first round of MouseFunc we used method (a). In the second round, we used method (b).

(a) Dynamic Weights

Cristiani et al (2002) introduced Kernel Target Alignment to measure the fit of a kernel matrix to a target matrix as Frobenius inner product between the two. If the target matrix Y is an outer product $\mathbf{y}^T\mathbf{y}$ of a label vector **y**, Kernel Target Alignment can be used as a measure of the fit of a kernel for a particular set of labels. All of our affinity networks W^h can be thought of as kernel matrices, as can any positively weighted sum of our affinity networks. As such we decided to optimize a variant of Kernel Target Alignment to set our network weights α_h. For our application, minimizing the Frobenius distance is equivalent to maximizing the Frobenius inner product.

The Frobenius distance between our composite affinity W^{comb} and the target matrix Y is:

$$d_F(W^{\mathtt{comb}}, Y) = \sqrt{\sum_i \sum_j (W_{ij}^{\mathtt{comb}} - Y_{ij})^2}\ .$$

Note that if W^{comb} and Y were transformed to vectors by stacking their columns atop one another, then their Euclidean distance would be the same as the Frobenius distance between the matrices. As such, we can optimize the Kernel Target Alignment with respect to our weights α_h using ordinary least squares (OLS) regression on the vectorized affinity networks W^h.

Table 1 The datasets that were used in MouseFunc 1st and 2nd submissions along with normalization procedure

Data	Normalization	1st submission	2nd submission
Su	Log	√	√
Sage	Log	√	√
Zhang		√	√
Protein-Protein interaction	Fisher Transformation	√	√
Interpro	Fisher Transformation	√	√
Pfam A	Fisher Transformation	√	√
Inparanoid	Fisher Transformation	√	√
Biomart	Fisher Transformation	√	√
Phenotype	Fisher Transformation	√	√
Disease	Fisher Transformation	√	√
BP annotation kernel	Fisher Transformation		√
CC annotation kernel	Fisher Transformation		√
MF annotation kernel	Fisher Transformation		√

In particular, if we were to construct a matrix Ω such that each column Ω_h of Ω is the vectorized version of the affinity network W^h. We could then solve the following ordinary least squares (OLS) problem:

$$A^\star = \mathtt{argmin}_A (\Omega A - \boldsymbol{t})^T(\Omega A - \boldsymbol{t})$$

We also include a bias term, α_0, by adding a column of "1"s, Ω_0, to Ω and discard this bias term when constructing the composite affinity network. The weight vector, $A^\star = (\alpha_0, \alpha_1, \ldots, \alpha_H)$, minimizes the Frobenius distance between the composite affinity network and the vectorized outer product, **t,** of the label vector **y**.

However, we reasoned that in gene function prediction problems there is little value to ensuring that two negatively labeled genes have high affinity in the composite network. As such, when we construct Ω_h we only include the elements of W^h, $w_{i,j}$, for which one or both of *i* and *j* are positively labeled. Note that we also exclude all the elements in W^h that include an unlabeled node when constructing Ω_h. The target vector **t** is similarly constructed. Intuitively, we are attempting to choose the network weights so that pairs of positively labeled genes have high similarity and pairs containing a positive and negative node have low similarity. The target value t_i for the affinity of an edge connecting two positives is $\frac{n^+n^+}{l^2}$ and the target affinity for a positive/negative pair is $-\frac{n^+n^-}{l^2}$. The solution to the OLS problem is $A^\star = (\Omega^T\Omega)^{-1}\Omega^T\boldsymbol{y}$.

To guarantee that our composite affinity network remains positive semidefinite, we adopt an iterative algorithm to ensure that our network weights remain positive. Namely, if $\alpha_i < 0$ for $i > 0$ then we discard column Ω_i and recalculate $A^\star$. If no affinity network is assigned a positive weight, we select the network *h* whose column Ω_h has the largest correlation coefficient with the target vector **t**.

(b) Dynamic Weights with prior

We observed that when predicting the most specific GO categories (categories with 3-10 positive examples) the obtained weight vector, as described above, often has a very few non-zero entries which leads to poor generalization presumably due to overfitting. To address this problem we adopt a Bayesian approach and put a prior on the OLS parameters A and σ^2. We decided to use the conjugate prior distribution for $p(A, \sigma^2)$ which is the normal-inverse-gamma and which factors as $p(A, \sigma^2) \sim p(A|\sigma^2)p(\sigma^2)$. Given the prior mean A_{prior} and the prior covariance Σ_{prior}, we set the network weights to the mean of the posterior which is:

$$\tilde{A} = (\Omega^T\Omega + \Sigma_{prior})^{-1}(\Omega^T\Omega A^\star + \Sigma_{prior}A_{prior})$$

where A^* is the OLS estimate of the weight vector described above, i.e. $A^\star = (\Omega^T\Omega)^{-1}\Omega^T\boldsymbol{y}$.We set the prior mean A_{prior} to be the mean of the network OLS weights computed over all of the categories in the same GO hierarchy, use a diagonal prior covariance matrix, Σ_{prior}, and set the diagonal entries as the variance of values used to calculate A_{prior}.

2. Methods

2.1 Affinity Networks

For all our predictions we used the 13 datasets listed in **Table 1**. To make affinity networks we first construct similarity matrices by using Pearson Correlation Coefficient (PCC) to measure pair-wise similarities. To ensure achievable time complexity, we sparsify the similarity matrices so that each gene has at most fifty non-zero similarity values, corresponding to its fifty most correlated neighbors. We restore symmetry in the sparsified matrices by replacing each value at the (i, j)position in the sparsified matrix with the maximum of (i, j) and (j, i) elements.

Fisher transformation: Prior to measuring pairwise similarities we transform all the binary valued data using what we call a Fisher transformation which replaces the 0's with $log(1-\alpha)$ and the 1's with, $-log(\alpha)$, where α is the frequency of 1 in the column. We then calculate PCCs on the transformed values. This

transformation ensures that genes that share 'rare' features are assigned higher similarity than those which share more 'common' features.

Log transformation: For some of the expression datasets, we log transformed the expression levels before calculating the PCC as shown in Table 1. For the Su dataset, we also subtracted 6 from the log-transformed levels and set any resulting negative values to 0.

Annotation kernels: In the second round, to aid in the prediction of novel annotations added to previously annotated genes, we constructed affinity networks (or kernels) to measure the similarity between gene annotations. To construct these kernels, we represented a gene's annotations as a binary vector whose elements corresponded to GO categories, so a "1" in the gth element would indicate that the gene was annotated in category *g*. We constructed three separate affinity networks for each of the three separate branches of the GO hierarchy. However, we would vary the annotation kernels slightly based on the GO category that we were predicting. In particular, when predicting GO category *g*, we would not consider annotations in either *g* itself or any of its descendants.

2.3 Negative Genes

In our initial submission, we labeled as negatives all genes that had some annotation but not one in the GO category *g* under consideration. For our second submission we changed the definition of the negative examples to exclude the genes that were annotated in categories ancestral to *g*. We did this because we reasoned that if novel annotations include annotating genes from a less specific to a more specific category, then genes annotated to an ancestor of *g* should not be considered as negatives. This new definition of negative examples contributed to an improvement in our accuracy in predicting novel annotations but resulted in slight decline in the accuracy of our test set predictions.

2.4 Experimental Framework

All GeneMANIA experiments were performed using our own MATLAB code. Prior to submission of our results we evaluated our prediction performance by 1 - Area Under the Curve (AUC) of Receiver Operating Characteristic (ROC) using 3 fold cross-validation. In total we predicted a score for all 21,603 genes for 1,726 GO Biological Processes categories, 326 GO Cellular Component categories, and 736 GO Molecular Function categories.

3. Authors' Contributions

QM conceived of the project. DR and QM designed the GeneMANIA algorithm. SM and DWF implemented the GeneMANIA algorithm used in the contest. SM collected the data, designed the affinity networks, and performed the experiments. CG contributed code to create affinity networks. SM and QM wrote the description of GeneMANIA. QM supervised the project and provided feedback.

References:

Cristianini, N., Shawe-Taylor, J., Elisseeff, A., Kandola, J. (2002) On kernel-target alignment. In Dietterich, T.G., Becker, S., and Ghahramani, Z., editors, *Advances in Neural Information Processing Systems 14*. MIT Press.

Zhou, O., Bousquet, J., Weston, J., Scholkopf, B. (2004) In *Advances in Neural Information Processing Systems (NIPS)*.**16**, 321-328.

Zhu, X., Ghahramani, Z., Lafferty, J. (2003) Semi-supervised learning using Gaussian fields and harmonic functions. In *Proc. of the 20th International Conference on Machine Learning (ICML)*, 912-919.

Method Description

The basis of our approach is the support vector machine (SVM) classifier, which has been used successfully for supervised learning on high-dimensional data in a number of different application domains (Vapnik 1995). We demonstrated in earlier work that SVM predictions in the context of the Gene Ontology (GO) can be improved by leveraging hierarchical relationships between GO terms (Barutcuoglu, Schapire et al. 2006). Our previous work, however, assumed a single SVM classifier per GO term on a single set of data features. Since we have several input datasets, heterogeneous in both the biological phenomena they capture as well as relevance for predicting function in the context of each GO node, we extended this approach to allow for robust integration of multiple datasets, while also leveraging the hierarchical relationships defined by the Gene Ontology.

Our approach can be summarized as follows. We train support vector machine classifiers on each input dataset individually for each GO term, using bootstrapping to characterize the reliability of each independent classifier and obtain an aggregate prediction. The output of the individual dataset SVM's is combined using a Bayesian framework, which also incorporates the hierarchy of the GO. For each term, the final predictions are selected from either stage of this process (e.g. after only Bayesian integration, or after Bayesian hierarchical correction) based on performance estimates from cross-validation. The details of this process are described below.

The classification process began with training individual SVM classifiers for each of the input datasets. For all datasets but the protein-protein interaction data, we trained an SVM with a linear kernel using the SVM^{light} implementation (Joachims et al, 1999). For the PPI data, we used a diffusion kernel as described in (Kondor and Lafferty 2002). We used bootstrap aggregation for all SVM's over 25 bootstrap samples, reporting the median output of all held-out SVM instances for each gene. Held-out values were also used to characterize SVM performance for each dataset and GO term, which was used in the Bayesian integration. Bayesian networks reflecting the hierarchical structure of the GO and performance characteristics of each classifier were constructed by assuming a hierarchy of hidden nodes corresponding to each GO term. The constraints on class membership implied by the hierarchy were encoded in the conditional probability tables. Each hidden node was also associated with a set of corresponding observed nodes, representing the output of the individual dataset SVM's for that particular GO term (SVM's for different datasets are treated as noisy, independent indicators of class membership). Cross-validation experiments revealed that the Bayesian integration of several independent datasets performed poorly in comparison to a single bagged SVM on a combined dataset for small GO terms (i.e. less than 20 genes), so we replaced the naive integration with a single observed node for the SVM on the complete dataset for those terms. The distributions of all SVM outputs for positive and negative examples were estimated from bootstrapping as described above. To make inference on our Bayesian networks feasible, we split each GO branch into several smaller subsets, each preserving the local neighborhood around each GO term. Using this approach, each GO term occurred in several Bayesian networks in different contexts. We selected the best-performing context for each GO term based on evaluation on held-out data. Specifically, marginal probabilities were computed for each gene-GO term pair, and the resulting AUC on held-out data was compared among all GO term contexts as well as to the

Bayesian integration without hierarchical correction. The output from the Bayes net or the SVM yielding the highest AUC on held-out data was chosen for each GO term. Further details and a complete performance characterization of our approach will be provided in a future publication.

Author's Contributions

YG was responsible for implementing and applying the approach. CLM assisted in applying the method and helped in interpreting the results. OGT supervised the effort and provided feedback throughout the process.

References

Barutcuoglu, Z., R. E. Schapire, et al. (2006). "Hierarchical multi-label prediction of gene function." Bioinformatics **22**(7): 830-6.

Efron, B. and R. Tibshirani (1993). An Introduction to the bootstrap. New York, Chapman & Hall.

Kondor, R. and J. Lafferty (2002). Diffusion Kernels on Graphs and Other Discrete Input Spaces. ICML.

Vapnik, V. N. (1995). The nature of statistical learning theory. New York, Springer.

Joachims,T. et al. (1999). Making large-Scale SVM Learning Practical. In Scho¨lkopf,B., Burges,C. and Smola,A. (eds), Advances in Kernel Methods—Support Vector Learning. MIT Press, Cambridge, MA.

Supplementary Methods for Submission E

Methods for MouseFunc Gene Function Prediction

Chase Krumpelman, Wan Kyu Kim, Edward Marcotte

March 19, 2007

We combined two different strategies: a network based approach that identifies functional associations between genes and propagates GO terms via the linkages and a direct approach that employs an ensemble of classifiers to predict each gene's GO annotations from the available data. These two approaches are complementary: the network excels at finding shared terms based on correlations between gene features, while the feature based classifier finds strong associations between particular features and annotations, such as particular protein domains and enzymatic activity.

1 Network-based Prediction

The network-based prediction was performed in two steps. First, the probability of sharing a GO term was predicted for all possible gene pairs in the training set. Second, the GO terms of a target gene were inferred by propagating the GO terms of the gene's network neighbors.

1.1 Probability of sharing GO term(s) between two genes

Eight pairwise features were used: protein interactions, MGI phenotypes, OMIM diseases, shared domains (Pfam, InterPro) and mRNA co-expression (Zhang, Su and SAGE). Each feature was scored using one of three scoring schemes: hypergeometric, frequency, or Pearson correlation coefficient.

1. Hypergeometric probability - used for protein interactions

$$-log\ P = -log\Big(\sum_{i=1}^{min(n,m)} p(i|\, n, m, N)\Big),\ where$$

$$P(i|\, n, m, N) = \frac{\binom{n}{i}\binom{N-n}{m-i}}{\binom{N}{m}},$$

   and where n, m = the number of interactions in which each protein A, B is involved, respectively, and N = the total number of interactions. P indicates how likely the protein pair (A, B) is observed to interact one or more times by random chance. $-logP$ is taken as the score value.

2. Frequency - used for MGI, OMIM, Pfam, InterPro
   Each gene has a feature vector (v_i) of length n, where each vector element represents the presence(1) or absence(0) of a phenotype, a disease or a domain. $-logP$ is taken as the score, where P is the probability of sharing vector elements by chance.

$$-logP = \sum_{i=1}^{n} -log\Big(\frac{f_i}{N}\Big)^2$$

   where f_i is the frequency of the element i among the N total genes.

3. Correlation coefficient - used for gene expression (Zhang, Su and SAGE)
   We calculated the Pearson correlation between mRNA expression vectors. Gene pairs with $correlation < 0.5$ were filtered out.

The training set consisted of all pairs of genes in which both genes have GO annotation and show at least one functional linkage, e.g. share a Pfam domain, $correlation > 0.5$ etc.

In each BP, MF and CC, the gene pairs that shared at least one GO term were given a label of "1" and those not sharing any term were given a "0". The posterior probability of sharing at least one GO term was predicted using a naïve Bayesian classifier (implemented in the *Orange* data mining toolkit [2]) after the scores for each feature were discretized into four bins of equal intervals.

1.2 Network Prediction

A network was constructed between genes with edge weights equal to the predicted probabilities from the previous section. GO terms were predicted for a target gene by propagating the GO terms of its neighboring genes in the network. Given k neighbors $(n_1, n_2 ... n_k)$ of a target gene, the prediction score, S for each GO term (G_i), was calculated as :

$$S_i = 1 - \prod_k (1 - P_k)$$

where P_k is the edge weight between the target gene and its k-th neighbor [4].

2 Feature-Based Classifier Construction

The most direct approach to learning GO term assignments is to use a classifier to learn a mapping from feature vectors to GO labels. Our intuition was that this approach - feature-based classification - would be adept at finding strong connections between particular GO annotations and specific features in the data, for example, a particular protein domain being a strong indicator of an enzymatic molecular function.

2.1 Features

For computational efficiency, we constructed binary feature vectors for each gene. Several of the MouseFunc datasets (InterPro, Pfam, OMIM) were already in this form and were used directly. For the real-valued microarray datasets (SAGE, Su, Zhang), we constructed binary vectors as follows:

1. Perform singular value decomposition (SVD) on the dataset
2. Find the top 30 eigengenes
3. For each eigengene, find the Pearson correlation with every gene represented in the dataset
4. Assign the gene a “1” for this feature i if the correlation with eigengene i is greater than 1.5 standard deviations from the mean correlation, “0” otherwise

**dataset**	**dimension**	**coverage**
InterPro	5405	78.5%
Pfam	3133	72.1%
OMIM	2488	9.0%
sage	30	77.4%
Su	30	84.3%
Zhang	30	62.8%

Table 1: Datasets used for feature based classification

**classifier**	**notes**
Naïve Bayes	-Applied to all features
Decision Tree	-Applied to Mutual Information selected subset of 30 features
Boosted Tree	-Applied to Mutual Information selected subset of 30 features<br>-Using depth 2 trees as Weak Learner<br>-Boosting repeated 50 times

Table 2: Classifiers used for each dataset

Each microarray then provides a binary vector of length 30 where 1's indicate high correlation with a given eigengene.[1]

After this transformation of the microarray datasets, each of the six datasets was therefore represented as a binary vector as shown in Table 1.

2.2 Classifiers

It is notable in Table 1 that no dataset covers all of the genes. Very few genes (5%) have data present in all of the datasets; many genes have data present in only one or two datasets. We cannot expect a new gene presented for classification to have data present in for all the datasets; thus, we learned a classifier *for each dataset* and then constructed an overall classification from the results on the datasets that were present. In the next section, we give our approach for producing a score from a combination of the individual dataset classification results. In this section, we discuss the classification techniques we used for each dataset.

[1]This approach was inspired by Alter, et al. [3] which showed the potential biological relevance of correlation with eigengenes.

We applied three different classifiers to each dataset: Naïve Bayes [2], Decision Tree [3] and Boosted Decision Tree [4] (see Table 2).

2.2.1 Feature Selection

While Naïve Bayes, which constructs probabilities by counting, has computational complexity O(d) in the number of dimensions, trees check every dimension for a split at each node, and are O(d^2). Thus, the tree-based approaches require the input feature set to be pruned. We pruned each dataset by selecting the 30 features with the highest mutual information with the true labels.

2.3 Combining Classifiers

Each classifier's contribution to the final prediction for a dataset was weighted by its log-likelihood ratio of being correct on the training data. The combiner, which also factored in the prior log-likelihood ratio, was essentially a Naïve Bayes classifier on the features of the three base classifiers.

3 Feature-Based Classification

To apply the classification system described above, we loaded each gene and applied each present dataset's classifier/combiner. We then took the highest score among each dataset as the overall feature-based prediction. The complete feature based system is shown in Figure 3.

4 Combination of Network and Feature-Based Results

We combined the GO term predictions from the network with the feature-based predictions by taking the maximum score of the two methods for each gene and each term.

[2] Implemented by us in Matlab.
[3] Using **treefit** and **treeval** from the Matlab Stats Toolbox. [1]
[4] Using the **GML AdaBoost Matlab Toolbox** [5]

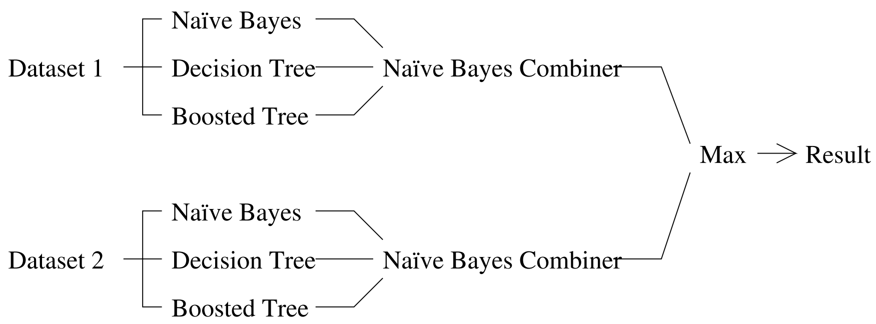

Figure 1: Diagram of Feature Based Classification System. For a given gene, a classification for each present dataset was made using a weighted combination of classifiers. The ultimate prediction for a gene was the maximum score taken across all of the present datasets.

Notes

Due to the running time of our network construction algorithm, our initial prediction for the newly-annotated gene set was based only on the feature-based classification approach (Fig 1b, group E). The prediction by the combined score was performed later (Fig 2b, group E*). The phylogenetic profile data were not used in our results.

References

[1] http://www.mathworks.com/products/statistics/.

[2] Orange data mining toolkit for python. http://www.ailab.si/orange.

[3] O. Alter, P.O. Brown, and D. Botstein. Singular value decomposition for genome-wide expression data processing and modeling. *Proceedings of the National Academy of Sciences*, 97:10101–10106, August 2000.

[4] Insuk Lee, Shailesh V Date, Alex T Adai, and Edward M Marcotte. A probabilistic functional network of yeast genes. *Science*, 306(5701):1555–1558, Nov 2004.

[5] Alexander Vezhnevets. Graphics and Media Lab, Department of Computer Science, Moscow State University.

http://research.graphicon.ru/computer-vision/machine-learning/gml-adaboost-matlab-toolbox.html.

Supplementary Methods for Submission F

Method Description: GeneFAS

Trupti Joshi, Chao Zhang, Guan Ning Lin, and Dong Xu

Digital Biology Laboratory
Computer Science Department
Christopher S. Bond Life Sciences Center
University of Missouri-Columbia

We used our method GeneFAS (Gene Function Annotation System) [1] for gene function prediction by utilizing all types of mouse high-throughput data provided by the organizers. We quantified the relationship between functional similarity and high-throughput data, and coded the relationship into 'functional linkage graph', where each node represents one protein and the weight of each edge is characterized by the Bayesian probability of function similarity between two proteins. We developed a Bayesian statistical method together with Boltzmann machine and simulated annealing for protein functional annotation through integrating various high-throughput biological data, including microarray, sage data, inparanoid, phylogenetic, and domains information. GeneFAS has been developed in both a GUI [2] and a command line version. The method description is as follows:

1: Processing of raw data

We consider all the provided raw data and process them to create binary interactions between two genes. In case of microarray, SAGE, inparanoid and phylogenetic we calculate correlation coefficient between the two genes. For domains (Pfam, Interpro) data we created binary interactions between two genes by comparing their domain profiles and representing the relationship between them with a ratio of the intersection over the union of the number of domains shared between them. For protein-protein interactions data we treat them as an association between the two proteins without computing correlation coefficient.

2: Function Prediction

Upon processing the raw data we calculated the Bayesian probability between a pair of genes based on the provided data and then predicted the functions using Boltzmann machine.

2.1 Measurement of protein function similarity

The Gene Ontology annotations have a hierarchical structure with multiple inheritances. We generated a numerical GO INDEX, which represents the hierarchical structure of the classification for all the 3 ontologies individually [3]. The more detailed level of the GO INDEX, the more specific is the function assigned to a protein. The maximum level of GO INDEX is 14. The following shows an example of GO INDEX hierarchy, with the numbers on the left giving the GO INDICES and the numbers in the brackets indicating the GO IDs:

GO Index   Function (GO ID)
2 cellular process (GO:0009987)

2-1 cell communication (GO:0007154)
2-1-8 signal transduction (GO:0007165)
2-1-8-1 cell surface receptor linked signal transduction (GO:0007166)
2-1-8-1- 4 G-protein coupled receptor protein signaling pathway (GO:0030454)
2-1-8-4-4-12 signal transduction during conjugation with cellular fusion (GO:0000750)

2.2 Calculation of Bayesian probabilities

We calculated probabilities for two genes to share the same function based on different types of high-throughput data [4]. Given two genes are correlated with Pearson correlation coefficient r as in microarray data (Mr), the posterior probability that two genes have the same function, $p(S|M_r)$, is computed using the Bayes' formulas:

$$p(S|M_r) = \frac{p(M_r \mid S)\, p(S)}{p(M_r)} \qquad (1)$$

where S represents the event that two genes have the same function at a given level of GO INDEX, $p(M_r|S)$ is the conditional (*a priori*) probability that two genes are correlated in their expression profile with correlation coefficient r, given that two genes have the same level of GO INDEX. The probability $p(S)$ is the probability of proteins whose functions are similar at the given level of GO INDEX by chance. The probabilities $p(M_r|S)$ and $p(S)$ are computed based on a set of proteins whose functions have been annotated in GO biological process. The probability $p(Mr)$ is the frequency of gene expression correlated with coefficient r over all gene pairs in mouse, which is calculated from the gene expression profiles.

To quantify the gene function relationship among the correlated gene expression pairs, we calculated the probabilities of such gene expression correlated pairs sharing the same function at each GO INDEX level. We similarly applied the Bayes formula for the other high-throughput data including SAGE, inparanoid, phylogenetics and domains. Figure 1 shows the probabilities of pairs sharing the same levels of GO indices versus Pearson correlation coefficient for the inparanoid data from mouse. It shows a higher probability of sharing the same function for broader functional categories (the high-order GO INDEX levels), or more correlated genes in their inparanoid data. Based on Figure 1, we decided to consider pairs with the correlation coefficient from the inparanoid data >0.5 for function predictions, as other pairs have little information for function prediction.

For protein protein interaction data (P), the probability that two proteins have the same function, $p(S|M)$, is computed as:

$$p(S|\mathrm{P}) = \frac{p(P \mid S)\, p(S)}{p(P)} \qquad (2)$$

where S represents the event that two proteins have the same function at a given GO INDEX level. $p(\mathrm{P}|S)$ is the probability for two proteins to have a protein binary interaction when they share the same function. The prior probability $p(S)$ is the relative frequency of proteins whose functions are similar. The probabilities of $p(\mathrm{P}|S)$ and $p(S)$ are computed based on the set of proteins whose functions have been annotated in the GO biological process. The probability $p(\mathrm{P})$ is the relative frequency of two proteins having a known protein interaction over all possible pairs in mouse, which is estimated from the known protein interaction data set.

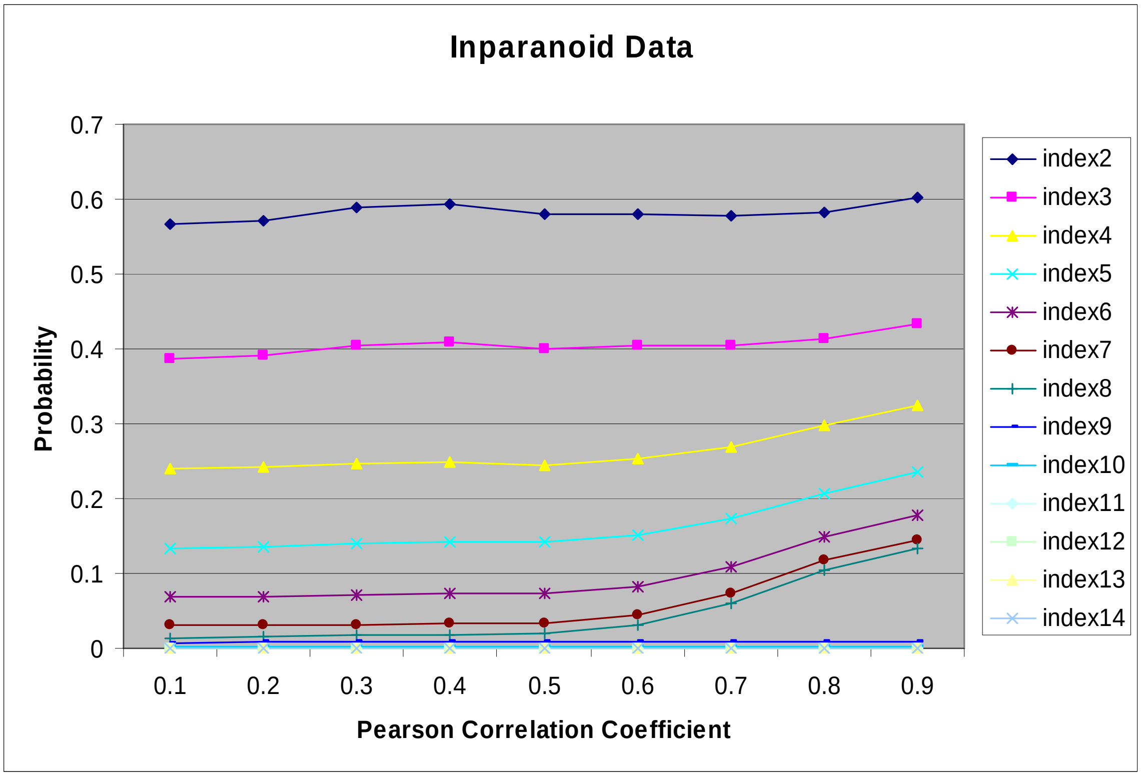

Figure 1. Probabilities of pairs sharing the same levels of GO indices versus Pearson correlation coefficient for the inparanoid data from mouse.

2.3 Global prediction.

A major limitation of the many prediction methods is that they only use the information of immediate neighbors in a graph to predict a protein's function. In some cases, the uncharacterized proteins may not have any interacting partners with known function annotation, and its function cannot be predicted using the local prediction method. In addition, the global properties of the graph are underutilized since this analysis does not include the links among proteins of unknown functions.

To address this issue we developed a new approach based on the global optimization strategy for predicting protein function. We used the Boltzmann machine to characterize the global stochastic behavior of the network [4]. A protein can be assigned to multiple functional classes, each with a certain probability. In the Boltzmann machine, we consider a physical system with a set of states, a, each of which has energy *Ha*. In thermal equilibrium, given a temperature *T*, each of the possible states α occurs with probability:

$$\mathrm{P}_{\alpha} = \frac{1}{Z} e^{-H_{\alpha}/K_B T} \qquad (3)$$

where the normalizing factor $Z = \sum_{\alpha} e^{-H_{\alpha}/K_B T}$ and K_B is the Boltzmann's constant. This is called the Boltzmann-Gibbs distribution. It is usually derived from the general

assumptions about microscopic dynamics. It is also applied to a stochastic network. In an undirected graphical model with binary-valued nodes, each node (protein) i in the network has only one state value Z (1 or 0). In our case, Z = 1 means that the corresponding node (protein/gene) has either known functions or predicted functions assigned to the node. Now, we consider the system going through a dynamic process from non-equilibrium to equilibrium, which corresponds to the optimization process for the function prediction. For the state at time t (optimization integration step t), node i has the probability for Zt,i to be 1, $P(Z_{t,i} = 1 | Z_{t-1, j \neq i})$ and the probability is given as a sigmoid function of the inputs from all the other nodes at time t -1:

$$P(Z_{t,i} = 1 | Z_{t-1, j\neq i}) = \frac{1}{1+e^{-\alpha \sum_{j\neq i} W_{ij} Z_{t-1, j\neq i}}} \qquad (4)$$

where α is a parameter reversely proportional to the annealing temperature and *Wij* is the weight of the edge connecting proteins i and j in the interaction graph. *Wij* is calculated by combining the evidence with correlation coefficient > 0.5 from microarray (M), inparanoid (I), phylogenetics (H), domains (D) and protein-protein interaction (P):

$$W_{ij} = \sum_{Fi} \delta j G(F_k | i, j) \qquad (5)$$

$$W_{ij} = \sum_{Fi} \delta j (1-(1-P(S_l | M))(1-P(S_l | I))(1-P(S_l | H))(1-P(S_l | D))(1-P(S_l | P))) \quad (6)$$

where S_k represents the event that two proteins *i* and *j* have the same function F_k (k = 1, 2, …, n), whose GO INDEX has *l* levels, *l* = 1, 2, …,12. $P(S_l|M)$, $P(S_l|I)$, $P(S_l|H)$, $P(S_l|D)$ and $P(S_l|P)$ were estimated probabilities retrieved from the probability curves calculated in section 2.2. δj is the modifying weight:

$$\delta_j = \begin{cases} 1 & \text{if } j \in \text{annotated proteins} \\ P(Z_{t-1,j} = 1) & \text{otherwise.} \end{cases}$$

To achieve the global optimization, we applied the simulated annealing technique as the following process: first, we set the initial state of all unannotated proteins (nodes) to be 0 or 1 randomly. The state of any annotated protein is always 1. If an unannotated protein is assigned with the state 1, its function will be predicted based on its immediate neighbors with known functions, using the local prediction method. Next, starting with a high temperature, pick a node i and compute *Pi* according to Equation 4, then update its state to 1 if the probability *Pi* is above a certain threshold. Each update of function prediction is based on its immediate neighbors with state 1 (i.e. known functions or predicted functions in the previous steps), using the local prediction method. The iterations are done till all the nodes in the network reach the equilibrium. With gradually cooling down, the system is likely to settle in a global optimal state of the network configuration.

3: Comparison with Prior Methods

We have compared GeneFAS against a few other methods using a set of yeast high-throughput data [4]. GeneFAS covered more unannotated proteins for functional predictions than any other methods published before (see Table 1), in particular Schwikowski et al. (2000) [5], Deng et al. (2002) [6], Letovsky and Kasif (2003) [7],

Vazquez et al.(2003) [8], Karaoz et al. (2004) [9]. One improvement of our method is that we can assign unannotated proteins into deeper levels of biological processes while the most other methods make protein function prediction using functional categories defined in YPD (http://www.incyte.com) or MIPS (http://mips.gfs.de) databases.

Prediction methods	Number of unannotated proteins with predicted function
GeneFAS	1802
Schwikowski et al. (2000)	364
Deng et al. (2002)	422
Letovsky and Kasif (2003)	320
Troyanskaya et al. (2003)	No information
Vazquez et al. (2003)	441
Karaoz et al. (2004)	>200

Table 1: The prediction methods and the number of proteins with predicted functions. Some of the increased performance of our method might be due to the different size of dataset used in different studies, but we believe it does not account for the major improvement of our method. The major contribution is that our method integrated multiple sources of data and utilized maximal information by combining and propagating information systematically across the entire network based on the global optimization

4. Contributions of each author

TJ and DX designed the prediction protocols. CZ modified GeneFAS to handle the provided data and ran the gene function predictions. GL processed the input data and calculated the Bayesian probabilities. TJ calculated the ROC curves using the training data sets and validated the prediction protocols. DX provided high-level technical guidance for the project. All the authors worked closely to deliver the predictions collectively.

References

1. Joshi, T., Chen, Y., Becker, J. Alexandrov, N. Xu, D. Genome-Scale Gene Function Prediction Using Multiple Sources of High-Throughput Data in Yeast Saccharomyces cerevisiae. OMICS: A Journal of Integrative Biology Dec 2004, Vol. 8, No. 4: 322-333.
2. Trupti Joshi, Chao Zhang, Guan Ning Lin, Zhao Song, Dong Xu. GeneFAS : A tool for prediction of protein function using multiple sources of data In “Methods in Molecular Biology: ‘Genomics Protocols’ “ Volume 2. In Press.
3. Trupti Joshi, Yu Chen, Nickolai Alexandrov, Dong Xu. Cellular Function Prediction and Biological Pathway Discovery in Arabidopsis thaliana Using Microarray Data. International Journal of Bioinformatics Research and Applications. Vol 1:335-350, 2005.
4. Chen, Y. and Xu, D. Global Protein Function Annotation through Mining Genome-Scale Data in Yeast Saccharomyces cerevisiae. Nucleic Acid Research. 32:6414-6424, 2004.
5. Schwikowski, B., Uetz, P., and Fields, S. (2000) A Network of Protein-protein Interactions in Yeast. *Nature Biotechnology.* 18:1257-1261.

6. Deng M. H., Zhang K., Mehta S., Chen T. and Sun F.Z., (2002) Prediction of protein function using protein-protein interaction data, *The first IEEE Computer Society bioinformatics conference, CSB2002,* 117-126.

7. Letovsky, S. and Kasif, S. (2003) Predicting protein function from protein/protein interaction data: a probabilistic approach, *Bioinformatics*, 19 Suppl 1:I197-I204

8. Vazquez, A., Flammini, A., Maritan, A. and Vespignani, A. (2003) Global protein function prediction from protein-protein interaction networks**.** *Nat Biotechnol*. 21:697-700.

9. Karaoz U., Murali T. M., Letovsky S., Zheng Y., Ding C., Cantor C. R. and Kasif S., (2004) Whole-genome annotation by using evidence integration in functional-linkage networks, *Proc Natl Acad Sci U S A.* 101:2888-2893.

matrix name(s)	full name
zhang	Zhang et al microarray data
su	Su et al microarray data (Affymetrix)
sage	Mouse Atlas of Gene Expression SAGE data
pfam	Sanger Institute Pfam domain data
interpro	EBI InterPro domain data
partition, *mcode*	protein-protein interactions data
phenotype	MGI phenotype ontology data
biomart	bioMart phylogenetic profile data
inparanoid	Inparanoid phylogenetic profile data
omim	OMIM (via homology) disease data

Table 1: Datasets (full names and short names used throughout the methods description) employed by the random forests classifiers.

1 Training Dataset Organization

Much of the data used by the classifiers was obtained directly from the shared Mousefunc dataset (i.e. that data available to all participants). Table 1 enumerates the datasets used by the classifiers, and provides the short names that will be used throughout the remainder of this document. Any post-processing performed on each dataset will be described in the following subsections (each devoted to one dataset).

For each dataset, the classifiers considered the data solely as a binary matrix, with the rows corresponding to genes (examples), and the columns corresponding to the dataset categories available (or computed through post-processing). An entry of 1 in any cell (i, j) indicates that gene i has the attribute j for that dataset.

The training data is provided for all of the 21603 *Mus musculus* genes, although for many of the genes particular datasets in the training data are missing.

1.1 Expression data

Three expression datasets were provided, two from microarray experiments (Su et al. 2004; Zhang et al. 2004), and a third using Serial Analysis of Gene Expression (Velculescu et al. 1995). While the details of preprocessing vary

for each dataset (see below), in broad outline the process went as follows: gene expression data was filtered using the prebuilt binary of Cluster 3.0 [6], run on Windows, and without modification. The filtered data was clustered on a Linux computer, using the source code for Cluster 3.0.

All datasets were clustered by k-means and agglutinative hierarchical methods. The hierarchical trees were generated using both the complete- and average-linkage variants, then cut at various points, to produce a predetermined number of clusters. The number of clusters was one of: 10, 20, 50, 100, 200, 500, and 1000 for microarray data, and one of 10, 100, and 1000 for SAGE data.

Finally, the clusters were combined into a binary attribute matrix (using the R programming language [7]), where each attribute (column) represents membership of a single cluster, with a "1" indicating membership, and a "0" lack thereof. For example, cutting a hierarchical tree into 200 clusters generates 200 new attributes. Therefore, using all such cuts generates 1880 attributes for each application of a clustering method to a dataset. In all, this creates $3 \cdot 3 \cdot 1880 \approx 17000$ new attributes, applying three clustering algorithms to each of three datasets. Knowledge that attributes vary widely in the frequency of their occurrence led us to cover as wide a range of cluster sizes as possible, since information about less-common gene attributes might more easily be gleaned from small clusters, and similarly for more-common genes in large clusters. There is no penalty to this, since the feature-selection properties of the machine-learning algorithms would later retain only those attributes which proved sufficiently informative.

The final matrices for each dataset are called *sage*, *su*, and *zhang*; with further details provided below.

1.1.1 Mouse Atlas of Gene Expression SAGE data

The average tag counts for the SAGE dataset were selected for analysis. Each column (i.e. library) was first normalized by its sum, to remove library-specific effects. Libraries with the same name were then averaged (e.g., the two "Kidney" libraries were combined, but kept distinct from the "Kidney — Whole" library). The number of copies ranged from one (the most common) up to six (for "Brain, whole head" and "Prostate, dorsal lobe").

Clustering methods were applied using Canberra distance, which is appropriate for count-based (Poisson-like) data. This metric, defined as the sum of the element-wise ratios of the difference of the two expression pro-

files to their sum, tends to accentuate the effect of a given difference among profiles close to zero. There is evidence [8] that a distance metric which acknowledges the poisson nature of a counting-based method such as SAGE, may result in clusters of superior biological signifance. This is to be expected since the variance of a poisson process increase with the mean, with the result that a given fixed difference is more significant for smaller values. Euclidean distance was also used.

This latter metric was applied both with and without log-transformation. Since this method is based on counting tags, there is a natural asymmetry to the data, and it was felt that mean- or median-centering was not appropriate. No filtering was performed on the basis of variation in counts across libraries for a given gene.

1.1.2 Microarray-based gene expression data

The approach for the array-based datasets was somewhat different. First, profiles corresponding to the same GID were averaged. A $\log_2$-transformation was applied to the Su dataset, which showed a much larger dynamic range in signal strength across tissues, than the Zhang data. Next, genes were removed which did not rise sufficiently above the baseline sufficiently (at least 40 observations strictly greater than zero for Zhang; at least 40 observations above 3.0 for Su). Genes were then removed if they did not show sufficient variation across the tissues (defined as the difference between their maximum and minimum values; they had to exceed a threshold of 1.0 (Zhang) or 3.0 (Su)). The remaining data were clustered using both k-means and agglomerative hierarchical clustering, as described earlier. Both Pearson and absolute-value-of-Pearson correlation methods were used, with centering.

1.2 Protein domain data

Protein domain data was provided only in binary form via the files `interpro_domainData.txt` and `pfamA_domainData.txt`. The data in these files was mapped directly to the *interpro* and *pfam* matrices, with 5405 and 3133 columns, respectively. Each column corresponds to a distinct InterPro or Pfam protein domain, with a "1" for cell (i, j) indicating sufficeint evidence that gene i possesses domain j, "0" otherwise.

1.3 Protein-protein interaction data

The protein-protein interaction data (available in the `ppi_adjacency.txt` file) was clustered into complexes using Cytoscape version 2.2 [4], with the MCODE version 1.1 plugin [5]. The default memory allocation for the Java Virtual Machine was increased significantly, to make maximum use of available memory, but default parameters were used for the MCODE algorithm itself. Eighty-eight clusters were obtained, ranging in size from three up to ninety-seven proteins. The binary attribute *mcode* matrix was produced (using a Perl program) consisting of 97 columns, corresponding to the MCODE-generated clusters. A "1" at cell (i, j) indicates membership of gene i in complex j, while a "0" indicates the lack of membership.

1.4 Phenotype data

Phenotype data was processed in binary form via the file `MPheno_OBO.ontology`. The organizational structure of the phenotype ontology (i.e. the definitions and structure found in the `MPheno_OBO.ontology` file) is ignored. The *phenotype* matrix then is a direct mapping of the data, and consists of 33 columns corresponding to each distinct "leaf" phenotype characteristic. A "1" at cell (i, j) indicates gene i has been shown to be linked in some fashion to phenotype j (usually via a knock-out experiment), "0" otherwise.

1.5 Phylogenetic profile data

The phylogenetic data is provided in both binary are real-valued forms, from two data sources: bioMart and Inparanoid. For both data sources, the real-valued data is ignored and only the data in the `phylogenetic_binary.txt` and `inparanoid_binary.txt` files are used. Each file maps directly to the *biomart* and *inparanoid* binary matrices with 18 and 24 columns, respectively. A "1" at cell (i, j) indicates sufficient evidence that gene i has some homologue in organism j, "0" otherwise.

1.6 Disease data

The OMIM disease data is provided only in binary form, and no further post-processing is performed on it. The *omim* matrix then consists of 2488 columns, corresponding to each distinct disease identified in the `omim_disease.txt`

file. A "1" at cell (i, j) indicates sufficient evidence that gene i has a homologue associated with disease j, "0" otherwise.

2 Target Dataset Organization

Provided in the MousefuncI datasets is the complete GO annotation record for 19885 of the 21603 *Mus musculus* genes. 5416 distinct GO terms are identified as having one of these genes annotated to them, although only 2815 of these terms are considered as targets for MousefuncI prediction. These target GO terms are broken up into twelve categories by a combination of branch and annotation count. The three branches are biological process (BP), molecular function (MF), and cellular component (CC); the annotation count groupings across each branch are $[3, 10]$, $[11-30]$, $[31-100]$, $[101-300]$.

It should be noted here that of the 19885 training examples, only 10542 have some GO annotation (not including the terms "molecular function unknown", "biological process unknown", and "cellular component unknown"). This subset plays an important role in the training of classifiers (described below).

3 Functional Linkage Classification

One type of classifier used in our MousefuncI entry is based on a functional linkage approach (similar to that described in ref. [**?**]). Here, *pairs* of genes are examined in order to predict the assignment of a GO term to a holdout gene.

3.1 Training Data for Functional Linkage Approach

3.1.1 Expression Data

The Pearson product-moment correlation coefficient was computed for each gene-pair using the *zhang*, *su*, and *sage* datasets (one gene-pair coefficient for each dataset). These coefficients are continuous (in the range $[0, 1]$), and for later use each gene pair is given membership in some of five non-disjoint sets $E_{0.5}$, $E_{0.6}$, $E_{0.7}$, $E_{0.8}$, and $E_{0.9}$; the sets are defined as follows: $E_x = \{(a, b) | \rho(a, b) \geq x\}$ (where a and b are genes, and $\rho(a, b)$ is the correlation coefficient between a and b). Across the three expression datasets, this results

in 15 binary attributes for gene pairs (a 1 indicating membership in the corresponding set, 0 otherwise).

3.1.2 Protein-protein Interactions Data

Since the protein-protein interaction data is provided as binary values for gene-pairs already (i.e. as a function $ppi : a \times b \to \{0, 1\}$), no further processing was done. This results in a single binary attribute for gene pairs (1 indicating experimental evidence of a protein-protein interaction, 0 otherwise). Note that this does not make use of the *partition* or *mcode* matrices, but instead relies on the original (unprocessed) data provided by the MousefuncI competition.

3.1.3 Protein Domain Data

For the *pfam* and *interpro* datasets, the Jaccard similarity coefficient was used to acquire a statistic (in the range $[0, 1]$) for each pair of genes. Let A and B represent sets for (respectively) genes a and b, where the set members are those protein domains identified by the dataset (*pfam* or *interpro*) assigned to the appropriate gene. Then the Jaccard similarity coefficient is defined as $\rho(a, b) = \frac{|A \cap B|}{|A \cup B|}$. Those gene-pairs (a, b) having $\rho(a, b) \geq 0.9$ are then assigned membership into a single set (a quantization method identical to that for the set $E_{0.9}$ described in §3.1.1). This processing results in two binary attributes for gene pairs (one for each of *interpro* and *pfam*).

3.1.4 Phenotype and Disease Data

The *phenotype* and *omim* datasets are given the same treatment as that applied for either of the *interpro* or *pfam* datasets (§3.1.3), resulting in two binary attributes for gene pairs (one each for *phenotype* and *omim*).

3.1.5 Phylogenetic Data

The *biomart* and *inparanoid* datasets are given the same treatment as that applied for either of the *interpro* or *pfam* datasets (§3.1.3), with a slight exception. Rather than creating only a single binary attribute for each dataset, three binary attributes are created instead, corresponding to Jaccard similarity coefficients equal to or greater than 0.7, 0.8, and 0.9. This is analogous to the quantization described in §3.1.1 for sets $E_{0.7}$, $E_{0.8}$, and $E_{0.9}$. Thus,

size binary attributes are constructed for phylogenetic data (three each for *biomart* and *inparanoid*).

3.1.6 Gene-Pair Attribute Summary

The previously identified gene-pair binary attributes are then combined into a single matrix, with gene-pairs as the rows and 26 distinct attributes representing the columns. This single matrix is further split into 12 sub-matrices (each disjoint row-wise, but sharing the same 26 columns), corresponding to the 12 distinct target GO term categories identified for performance assessment in the MousefuncI competition.

3.2 Target Data for Functional Linkage Approach

Since the MousefuncI competition target data provided for the training examples is simply a binary attribute assigned to each individual gene, some processing is needed for the functional linkage approach. A single target attribute is instead constructed for each sub-matrix constructed in §3.1.6. Two genes a and b (members of the training examples provided in MousefuncI) are considered to be "functionally linked" with respect to one of the 12 target GO categories if a and b are both annotated with a GO term in the same category (branch and cardinality) or same branch but with smaller cardinality. For example, two genes a and b are functionally linked with respect to the MF-$[11, 30]$ category if they share a GO term annotation that is in the MF branch with cardinality $[2, 30]$ (the lower bound is trivially at least 2, if both a and b have the annotation). Using this definition of functional linkage, a gene-pair binary response attribute is constructed for each of the twelve sub-matrices.

3.3 Decision Trees for Functional Linkage

3.3.1 Decision Tree Training

Twelve decision trees [2] (one for each GO prediction category) were then created for the $\binom{10542}{2}$ gene pairs (with some non-unknown GO annotation) using the 26 training attributes and the single functional linkage response attribute. Candidate node splits were assessed by computing the hypergeometric probability of the null hypothesis that the candidate split attribute and the response attribute are independent. The split with the lowest such

probability p^* emerges as the selected split. An early-stopping criteria was also employed to prevent further splits when $p^* > 10^{-8}$. This use of exact probabilities has been shown to produce simple trees with comparable error rates when compared to other selection and stopping criteria [3].

3.3.2 Decision Tree Classification

At each leaf node in the decision trees, the ratio of genes that are functionally linked to those that are not can then be treated as the probability of functional linkage for a new gene-pair being classified. A proportional pseudocount approach is used to handle pure leaf nodes, so the estimated probability of a gene-pair being functionally linked is equal to $\frac{T+\alpha}{T+F+1}$, where T is the number of functionally linked training gene-pairs found at that node, F is the number of non-functionally linked training gene-pairs found at that node, and α is the proportion of functionally linked training gene-pairs among all the training gene-pairs. Since the decision trees classify gene-pairs, and do not directly provide scores for single genes being annotated with single GO attributes, a further step is needed. To determine if an unknown (held-out) gene a is annotated with a particular GO term, we first collect all known genes (training examples) assigned to that GO term (call this set of genes B). The probability of functional linkage is then computed for all gene-pairs $(a, b)|b \in B$ using the appropriate (corresponding to the twelve categories)) decision tree. The top three such probabilities are then averaged and taken to be the probability that gene a is (or should be) annotated with the GO term.

4 Random Forests Classification

A variant on random forests is used to classify each of the specified target GO terms.

4.1 Random Forests Described

Random forests [1] are part of a machine learning family called *ensemble classifiers*. Each classifier (forest) consists of a set of individual decision trees [2]. Each tree is trained on a bootstrapped sample of the examples (training genes), with the (approximately 1/3) non-sampled genes used to estimate

performance — called out-of-bag error). At each internal node in a tree, a sampling (without replacement) of the remaining variables (columns) are considered (via the information gain measure) for a binary split. The training instances are partitioned accordingly, and the split variable is no longer considered for further splits (as it can only result in zero-gain future splits). Traditional random forests do not employ early-stopping (pre-pruning), instead forcing the splitting to continue until a pure node has been reached (a leaf), or until all possible splits (columns) have been exhausted.

4.1.1 Early Stopping Criteria

Unlike strict random forests, the classifiers here do employ an early-stopping criteria. Once the best candidate split has been found the likelihood of the split is considered against a null hypothesis that there is not dependence between the split variable and the response variable (the GO term annotations). This likelihood l is measured directly via a hypergeometric distribution, and is thus easily computable. A number of cutoff thresholds have been examined, with a final selection of $l \leq 0.05$ indicating that a split should be made, and $l > 0.05$ resulting in the construction of a (possibly impure) leaf. The early-stopping is used due to the very large number of predictive variables in the training matrices and the low probability that any one (or boolean combination of some) leads to a pure leaf. The practical result of this is many long branches extending to the depth of the number of possible split variables, increasing computation time significantly.

Using a statistical measure of likelihood as an early-stopping criteria has been observed to be effective, especially when combined with using the same measure for assessing the quality of a candidate split [3]. Computing the hypergemoetric probability for each candidate split at each node, however, proved to be too computationally costly, leading to the adoption of the information gain split measurement, with likelihoods (for early-stopping) computed only for winning candidates at each node.

4.1.2 Method of Classification

Classification of new instances is performed by traversing down each tree in the forest, making the appropriate splits according to the values of the training variables for the new instance. When a leaf is reached, the current tree "votes" by reporting the percentage of training instances at that leaf

that have a “1” for the GO annotation. Thus, a pure leaf will provide a vote of 0.0 or 1.0, while impure leafs will range between those two extremes. A tally of votes across all trees is made, and normalized to the range $[0.0, 1.0]$ via a division by the number of trees. This final score represents the strength of belief of the forest that the new instance has the GO annotation.

4.1.3 Parameter Selection

The number of trees used in each forest is 100, and the sample size of candidate split variables at each node is 500. These values were selected by repeated trial-and-error, examining the performance estimates after each trial and altering the parameters accordingly. A more algorithmic approach can certainly be used to search in the parameter space for an optimal configuration, but given the time limitation of MousefuncI, this hands-on parameter selection method was deemed more feasible.

4.1.4 Out-Of-Bag Performance Estimates

One can used a variety of methods to evaluate random forests performance, including cross-validation. The two primary difficulties with cross-validation are bias in the dataset partitioning and the increased computation time required. Bias can occur when the holdout partitions are not distributed identically as the true expected data. When considering GO terms with fewer than 30 annotations (amongst the ~ 20000 genes), biased splits can lead to performance estimates that deviate from true performance by a significant margin. A solution to this is to repeat the cross validation run n times, often stated as “n-times-m-fold” cross-validation (usually with $m = n$). Unfortunately, this only increases the computation time further.

Ensemble classifiers provide an alternative error estimation measure, using the out-of-bag examples that are not sampled during the bootstrapping (1-fold sampling *with* replacement) performed at the start of each tree’s (or other ensemble classifier primary unit) training run. Those out-of-bag examples are classified by each tree not using them as a training example. This leads to approximately 1/3 of the trees being used to classify each training example. Previous studies have described this measure as being unbiased and accurate for estimating performance on holdout data that is identically distributed with the training data.

	BP	CC	MF
3–10	*interpro*<br>*pfam*<br>*phenotype*	*interpro*<br>*pfam*<br>*sage*	*interpro*<br>*omim*
11–30	*interpro*<br>*pfam*	*interpro*<br>*pfam*<br>*omim*	*interpro*<br>*pfam*<br>*biomart*
31–100	*interpro*<br>*pfam*<br>*phenotype*	*interpro*<br>*pfam*	*interpro*<br>*pfam*<br>*biomart*<br>*inparanoid*
101–300	*interpro*<br>*pfam*	*interpro*<br>*pfam*<br>*inparanoid*	*interpro*<br>*pfam*<br>*biomart*

Table 2: Datasets used for training classifiers on each GO term category.

4.2 Term-Specific Datasets

When training a classifier for a specific target GO term, it may be best to used only a subset of all the possible training variables available. This amounts to selecting specific columns out of the training dataset matrices, concatenating the chosen columns, and training the classifier on the resulting sub-matrix. Since there are 2815 target GO terms to be predicted, such an approach (fine-tuning for the optimal predictive variables for each GO term) is infeasible (especially when considering the time limitation of MousefuncI).

As an alternative approach, one can instead select for a subset of predictive variables for each of the twelve categories of target GO terms. Moreover, rather than find the specific subset of optimal predictive variables for each category, the problem can be simplified by finding the subset of datasets. A greedy approach is used to identify the combinations of datasets that lead to the best out-of-bag performance estimates. The method begins by training classifiers for a number of target GO terms in each category using each dataset matrix as the lone training data. For each category, select the dataset offering the best performance estimate, and repeat by incrementally adding the remaining unselected datasets. Continue until no significant gain in performance is observed. The result of this procedure identifies a subset of the datasets that are then used for full classification. Table 2 shows the datasets assigned to each of the GO term categories.

4.3 Training Set Pruning (Example Selection)

The MousefuncI competition contains two distinct parts. The first task is to predict the target GO term annotations for the held-out set of $21603 - 19885 = 1718$ genes. This is the testing analogue to assessing the performance of a classifier in predicting the annotations of a gene for which one has experimental data (i.e. the attributes used as columns in the matrices identified in table 1), but no GO annotation yet. The 1718 held-out test genes are selected uniformly from the mouse genes for which experimental data is available. For constructing classifiers used to make predictions for the target GO terms in this portion of the competition, all 19885 training genes are used as examples.

The second task of the MousefuncI competition is to *update* the set of target GO annotations for the 19885 training genes. Since the GO annotation data (the target data) corresponds to an early-2006 snapshot of the GO, and the competition results are submitted in late-2006, one expects some of the target GO terms to have additional annotations (amongst the 19885 training genes). To build classifiers used to make predictions for this portion of the competition, only the 10542 genes having some non-unknown GO annotation are used as training examples. Note that *any* non-unknown GO annotation qualifies for membership in this training example set, not just those genes with a *target* GO annotation. This choice reflects the notion that the competition will only consider for performance measurement those genes that have had some update, thus practically guaranteeing at least one GO annotation. The selection of this training set then is logical as one would like to train on data that is as closely representative (distributed identically) to the eventual test data.

4.4 Technical Specifications

The random forests code was written in the Java programming language (version 1.5), and executed primarily on Linux-based machines.

5 Combination of Functional Linkage and Random Forests Approaches

Both the functional linkage approach and the random forest approach give conditional probability estimates of a gene i belonging to the annotation set for go term t; call these two estimates p^+_{rf} and p^+_{fl}, respectively. To combine these estimators, a logistic regression method is employed according to the following model:

$$\ln \frac{p^+}{1-p^+} = \alpha \cdot \ln \frac{p^+_{rf}}{1-p^+_{rf}} + (1-\alpha) \cdot \ln \frac{p^+_{fl}}{1-p^+_{fl}},$$

from which one can solve for p^+ as:

$$p^+ = \frac{\exp(\alpha \cdot \ln \frac{p^+_{rf}}{1-p^+_{rf}} + (1-\alpha) \cdot \ln \frac{p^+_{fl}}{1-p^+_{fl}})}{1+\exp(\alpha \cdot \ln \frac{p^+_{rf}}{1-p^+_{rf}} + (1-\alpha) \cdot \ln \frac{p^+_{fl}}{1-p^+_{fl}})}.$$

The single parameter α was found using the training data via out-of-bag estimates (for the random forests) and cross-validation (for the functional linkage decision trees). The area under the curve (AUC) of a precision-recall plot (precision $= \frac{TP}{TP+FP}$, recall $= \frac{TP}{TP+FN}$) was chosen as the metric for performance for each target GO term category, in an attempt to identify then the 12 values of α leading to the greatest performance. A "grid" method was adopted for this parameter search, starting first by ranging α over the choices $\{0, 0.1, 0.2, \ldots, 0.9, 1\}$, representing the best-performing value as α_{init}. Further refinement is perfomed by examining possible values within the range $[\alpha_{\text{init}} - 0.1, \alpha_{\text{init}} + 0.1]$, with increments of 0.2. The identified values for α using this method are shown in table 3.

6 Final Predictions

6.1 Test Set Predictions

Predictions for the held-out test set of genes were made by training the random forests on all provided training examples. The test set of genes were then classified using both the random forests and functional linkage approaches. The resulting scores were combined as described in §5.

	BP	CC	MF
3–10	0.52	0.58	0.72
11-30	0.62	0.62	0.80
31-100	0.66	0.48	0.76
101-300	0.50	0.34	0.86

Table 3: Selected values of α for the logisitic combination of random forest and functional linkage estimators.

6.2 Novel Predictions

Predictions for new annotations amongst the training examples were carried out by training the random forests only on those 10542 training examples with some non-unknown GO annotation. The training examples were then re-classified using both the random forests and functional linkage approaches. The resulting scores were combined as described in §5.

References

[1] Breiman, Leo. *Random Forests*. Machine Learning, 45 (1), 5–32, 2001.

[2] L. Breiman, J. Friedman, R. A. Olshen and C. J. Stone. *Classification and regression trees*. Wadsworth, 1984.

[3] Martin, J. Kent. *An Exact Probability Metric for Decision Tree Splitting and Stopping*. Machine Learning, 28, 257–291, 1997.

[4] Shannon, P., A. Markiel, O. Ozier, N.S. Baliga, J.T. Wang, D. Ramage, N. Amin, B. Schwikowski, and T. Ideker. 2003. *Cytoscape: A Software Environment for Integrated Models of Biomolecular Interaction Networks*. Genome Res. 13: 2498-2504.

[5] Bader, G.D. and C.W. Hogue. 2003. *An automated method for finding molecular complexes in large protein interaction networks*. BMC Bioinformatics 4: 2.

[6] de Hoon, M. 2003. *Cluster 3.0*

[7] R Development Core Team. 2006. *R: A Language and Environment for Statistical Computing*. R Foundation for Statistical Computing, Vienna, Austria.

[8] Cai, L., H. Huang, S. Blackshaw, J.S. Liu, C. Cepko, and W.H. Wong. 2004. *Clustering analysis of SAGE data using a Poisson approach*. Genome Biol 5: R51.

Supplementary Methods for Submission H

Protein Function Prediction Using 'Query Retrieval' Methods

Yanjun Qi [a], Judith Klein-Seetharaman [a,b], Ziv Bar-Joseph [a]

[a] School of Computer Science, Carnegie Mellon University, Pittsburgh, PA 15213 USA
[b]Department of Structural Biology, University of Pittsburgh School of Medicine, Pittsburgh, PA 15261 USA

Protein function prediction is one of the major challenges in computational biology. Here we present a 'Query Retrieval' method to predict the functions of proteins by combining a large number of high throughput biological datasets.

(Note that in this article with respect to the functional annotation task we use the term 'protein' and the term 'gene' exchangeably.)

1 Pairwise Similarity from Multiple Information Sources

Our underlying assumption for the prediction task is that similar proteins (with respect to some biological function) are more likely to share common attributes (features) when compared with proteins from different functions. Note that this does not imply that these shared properties are the same for all functional classes. For example, M phase cell cycle proteins may share similar expression patterns, but may differ in their domain contents, whereas transcription factors may have different expression patterns but similar sequence features. Thus, while same pairwise similarity scores are used for all functional categories, the importance of each specific similarity may depend on the specific functional category (see below where we discuss the classifier).

Table 1 lists the feature sources that we used for the "mouseFunc" [1] competition. We computed similarity scores for pairs of proteins with respect to each of the data sources. For instance, for gene expression data sets we applied the correlation coefficient to measure similarity for a given protein pair. For domain information we use the dot product as the similarity measure. For the remaining data sources, the dot product operations were applied similarly. Totally twelve similarity features were derived from six types of different data sources. Concatenating these features then gave us the feature vector describing a protein-protein pair.

Most biological datasets are noisy and contain many missing values. For example, the disease profile for most proteins is yet to be discovered. The fifth column in Table 1 describes the average coverage of the derived pairwise similarities from each feature source. As can be seen, different features have varied degrees of missing values. The coverage of the 12 features (Table 1) ranges from 2.6% for disease to over 83.1% for one of the gene expression features.

2 Protein Function Prediction through Query Retrieval

Here, we propose to handle this protein function prediction task by taking into account of two issues: (1) Considering the properties of protein function labels. (2) Combining evidence from multiple biological datasets.

The Gene Ontology (GO) [2] functional annotations define a set of terms (categories) for annotating proteins. We observe that for most GO terms only a very small number of positive examples exist currently. This naturally leads us to the 'Query Retrieval' scenario. The prediction of a test protein could rely on how similar this protein to the known positive examples for a particular GO category. Furthermore we could also extract some information from proteins not annotated to this category by considering the dissimilarity between a query protein to the negative examples.

Table 1: Pairwise similarity features collected for the protein function prediction task in M. musculus. Feature sources were downloaded from the "mouseFunc" competition website [1]. A total of twelve protein pairwise similarity features were extracted from six types of data sources. The first column lists the index number. The second column lists the name of the feature source. The third column lists the related file from which each feature is derived. The fourth column describes the method used to calculate the pairwise measure. The fifth column presents the percentage of pairs for which information is available using this feature.

No.	**Feature Name**	**Related File**	**Method**	**Coverage**
1	Co-expression	Zhang_expData.txt	Correlation Coeff.	0.4732
2	Co-expression	Su_expData.txt	Correlation Coeff.	0.8313
3	Co-expression	sage_expData_avgTagCounts.txt	Correlation Coeff.	0.7141
4	Domain Fusion	pfamA_domainData.txt	Dot Product	0.7356
5	Domain Fusion	interpro_domainData.txt	Dot Product	0.8266
6	Protein-protein Interaction	ppi_adjacency.txt	Dot Product	0.2746
7	Phenotype Similarity	MGI_phenotype.txt	Dot Product	0.0758
8	Phylogenetic Profile	phylogenetic_binary.txt	Dot Product	0.7116
9	Phylogenetic Profile	phylogenetic_scores.txt	Dot Product	0.7116
10	Phylogenetic Profile	inparanoid_binary.txt	Dot Product	0.6842
11	Phylogenetic Profile	inparanoid_scores.txt	Dot Product	0.6842
12	Disease Profile	omim_disease.txt	Dot Product	0.0264

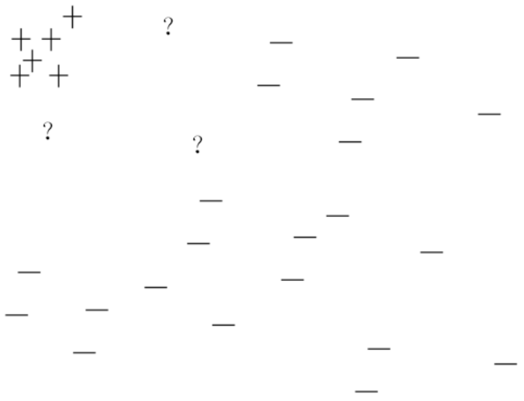

Figure 1: Query retrieval for the protein function prediction.

Figure 1 illustrates this basic idea. Treating a candidate test example as a query, our protein function classification problem aims to figure out how similar this query to the positive points. This objective is achieved by fitting the retrieval task into a classification framework. That is to distinguish between the positive example's similarity to other positive points and the negative examples' similarity to the positive set. If the similarity features between a query protein and positive proteins are quite similar to those pairwise features from positive-vs-positive pairs, and at the same time, quite different compared to those similarity features of the negative-vs-positive pairs, we would classify this query gene as a positive example for the current GO category.

Thus given a specific GO category A, we divide protein-protein pairs into two classes: positive pairs (both proteins belong to A) and negative pairs (one of the proteins belongs to A while the other does not). Negative examples are randomly selected from all proteins not assigned to category A. The positive and negative pairs are used to train a random forest (RF) classifier [3]. Given a test protein, we derive pairwise

features between that protein and all proteins assigned to A and use the RF model to classify the new pairs. If most pairs containing the test protein and a positive protein are classified as positive we assign that protein to A. Specifically the data examples related to this binary classification task include:

- Training Positive: protein pair with both proteins belong to A
- Training Negative: protein pair with one of the proteins belongs to A while the other does not
- Testing: protein pair with one of the proteins belongs to A while the other is the query protein

For each protein-protein pair, pairwise similarity features were derived from Table 1 and function as input attributes for the protein pair classification. In addition, in our submissions to the "mouseFunc" competition [1], the probability for a test gene to belong to a certain function uses the average value of all its evaluated pairs' RF predicted scores.

3 Random Forest Classifier

Random forest [3] uses a collection of independent decision trees instead of one tree. Denote by Θ the set of possible feature attributes and by $h(x, \Theta)$ a tree grown to classify a vector x. Using these notations a random forest f is defined as:

$$f = \{h(x, \Theta_k)\}, k = 1, 2, ..., K \quad (1)$$

Where $\Theta_k \subseteq \Theta$. That is, a random forest is a collection of trees, where each tree is grown using a subset of the possible attributes. For the k-th tree, Θ_k is randomly selected, and is independent of the past random vectors $\Theta_1, \Theta_2, ..., \Theta_{k-1}$. Each of the trees 'votes' for one of the classes and the most popular class is assigned to input x.

Specifically, the random forest is grown in the following way: Each tree is grown on a bootstrap sample of the training set (this helps in avoiding overfitting). A number m, which is smaller than d (d is the total number of attributes and $d = 12$ here) is specified, and for each node in the tree the split is chosen from m variables that are selected at random out of the total d attributes. Once the trees are grown, they can be used to estimate missing values by an iterative algorithm. If the extreme size imbalance exists between positive and negative examples, the RF sets different weights for the classes to balance the overall error rate. In general, we grew 300 trees in our experiments. For m, we used the default value that was equal to the square root of the feature dimension d.

The randomization and ensemble strategies within the RF make it robust to noise and allow it to handle missing values problems [3, 4]. In addition, the biological datasets used in this task are often correlated with each other and thus should not be treated as independent sources. The RF classifier does not make any assumptions about the correlations between the data, which makes it appropriate for the type of data available for the protein function prediction task.

In addition, the random forest classifier derives an implicit mapping relationship between input attributes and the target class label. The importance of each attribute depends on the training examples and the trained RF model. In our predictions, we consider only one GO functional category at a time. This means that for each GO term we have a distinct RF model tree trained for the corresponding protein pair classification task. Thus, while the same set of pairwise similarity features (Table 1) is used for all functional categories, the importance of each specific similarity depends on the particular category being evaluated.

4 Discussions

Though Gene Ontology annotations exhibit a hierarchy structure between various terms, in our prediction we consider only one category at a time. These GO categories exhibit large variations in the number of known proteins assigned to them. Table 2 adapted from "mouseFunc" [1] competition website presents the distribution over the sizes of the various GO categories. As can be seen, most GO categories have less than 30 genes. Thus, for the majority of GO categories, when determining if any of the test set genes should be assigned to these categories we can only rely on this small number of existing positive assignments.

For our 'Query Retrieval' method, the function prediction for a gene is based on the assumption that the similarity between a certain positive gene to the remaining positive genes is much greater than the similarity

Table 2: Size distribution of the training GO categories (from the "mouseFunc" [1] competition website. The first column represents the range of category size. Columns 2-4 represent the number of GO categories within this size range for the different GO terms.

Number of Assigned Genes	**BP**	**CC**	**MF**
3-10	952	151	475
11-30	435	97	142
31-100	239	48	111
101-300	100	30	35

between negative genes to the positive examples. That is, we assume that the proteins/genes assigned to a certain GO function term are similar to each other with respect to the collected data (features) (Table 1).

For those GO terms with only a few positive examples (less than thirty genes), this assumption is one of the very few reasonable frameworks for a learning strategy. However, this is not necessarily true for those GO terms with a larger positive set (more than thirty examples assigned). In this case, the collected data evidence (Table 1) could be transformed to describe a neighborhood structure among the proteins. For an unannotated protein, the functions of its neighbors contain information about the function of this unannotated one. Belief propagation or similar approaches could then be applied to deduce functions of a protein through the functions of its close graph neighborhoods.

Indeed, the evaluation results confirm our assumptions, that is: our predictions are reasonably well for the small GO categories (3-10 and 11-30 cases in Table 2) and not as good for the remaining cases (30-100 and 101-300 cases in Table 2). Apparently, most of the GO categories belongs to the small size cases where our algorithm performs well (we place in the top 3 for almost all such cases). These categories are arguably the hardest to predict and in many cases contain a more detailed description for proteins, making them more useful for researchers.

Considering the above facts, we believe that our 'Query Retrieval' strategy is useful for many functional prediction tasks.

5 Acknowledgements

The authors want to express sincere thanks to Naveena Yanamala for helping to run the code for the large number of GO functions on the Department of Structural Biology, University of Pittsburgh clusters.

References

[1] MouseFunc Competition: http://funspec.med.utoronto.ca/mouseFuncs_I/.

[2] The Gene Ontology Consortium. Gene ontology: tool for the unification of biology. *Nature Genet.*, 25:25–29, 2000.

[3] Breiman L. Random forests. *Machine Learning*, 45:5–32, 2001.

[4] Qi Y., Bar-Joseph Z., and Klein-Seetharaman J. Evaluation of different biological data and computational classification methods for use in protein interaction prediction. *PROTEINS: Structure, Function, and Bioinformatics.*, 63(3):490–500, 2006.

Supplementary Methods for Submission I

Methods

Michele Leone, Andrea Pagnani
ISI Foundation,
Turin, Italy

October 20, 2006

Introduction

This contribution is based on an adaptation of a message passing algorithm family - known in the literature as Belief Propagation (BP) - to the problem of assigning functions to a set of not annotated genes. The method was previously used in a benchmark study of partially annotated yeast protein-protein interaction network (PPI). Differently from that case, the graph used here was built using a mixture of PPI, Gene Arrays and Protein Domains information.

The method used relies on the topological properties of the interaction network built and on the analytic properties of a cost function defined on the basis on the network topology and the set of known functions assigned to a part of the vertices. Therefore, it is very flexible and portable on a wide variety of problems, but can only assign functions to genes/proteins drawing out of a given starting set. It cannot - in the present form - predict completely new functions.

It is in principle possible to use a message passing procedure also to optimize the heterogeneous graph construction from experimental data, as well as to assign meaningful weights to single links. This goes however beyond the scope of the present study.

Interactions

In this work we integrated three main source of data: the Protein Protein Interaction (PPI) graph, a graph inferred by the two Gene Arrays experiment (GA) (Zhang and Su dataset), and the graph inferred by Protein Domain data (PD).

- The GA graph has been inferred by means of the Pearson correlation coefficient between all couples of genes in the two sets of experiments. All couples with Pearson coefficient > 0.95 where associated with a possible functional interaction. We considered then the graph $\mathcal{G}_{GA}$ made from the intersection between the two graphs relative to the two different experiments: *i.e.* all edges in $\mathcal{G}_{GA}$ join two genes with a Pearson coefficient > 0.95 in both GA experiments. $\mathcal{G}_{GA}$ has 28393 edges and 1449 vertexes.

- Interpro and PfamA Protein Domains files were preprocessed in the following way: an interaction link was drawn between genes having at least one protein domain in common. A threshold was put on the significance of protein domains appearance, in the sense that domains that appeared a number of times greater than the threshold were considered too generic (i.e. not informative) from the functional point of view - and were therefore disregarded. In the present data files the threshold is set to 500. $\mathcal{G}_{PD}$ has 88419 edges and 11002 vertexes. In this case the interaction graph was taken as the union of the Interpro and the PfamA resulting graphs, after a compatibility test and a statistical comparison between the quality of the functional information contained if the PD and GA data sets (the test was based exclusively on the competition given data).

- For the PPI graph no preprocessing was needed. $\mathcal{G}_{PPI}$ has 32351 edges and 7125 vertexes.

Gene Array, PPI and Protein Domains originated graphs were subsequently merged in a union file used by the Belief Propagation algorithm. As a zeroth order hypothesis, all links were weighted uniformly, even if coming from different data sources and different pre-processing procedures. The graph $\mathcal{G} = \mathcal{G}_{GA} \cup \mathcal{G}_{PD} \cup \mathcal{G}_{PPI}$ has 147930 edges and 13590 vertexes.

Annotations

Given the large number of GO terms to be predicted we decided to prune backward each annotation, by leaving only the most specific term (*i.e.* the leaves in GO directed acyclic graph). More specifically the pruning is done as it follows: given a gene and starting from one of its most specific GO terms, the *is_a* and the *part_of* hierarchies are up followed pruning out all connected less specific GO terms.

Algorithm

Let $\mathcal{G}$ be our graph, with set of vertexes $V = \{1, \cdots, N\}$ representing the observed proteins, each protein name being assigned a numerical value form 1 to N. Let us also define a mapping between the set of all observed annotations and the numbered set $\mathcal{F} = \{1, \cdots, F\}$. Each protein i belonging to V can then be characterized via a discrete variable X_i that can take values $f \in \mathcal{F}$. One would like to compute the probability $P_i(f) = Pr(X_i = f)$ for each protein to have a given function f given the functions assigned to the proteins in the rest of the graph. The method is extensively explained in [2].

Given sets V, A and $V \backslash A$, the interaction graph $\mathcal{G}$, the graph of unclassified proteins $\mathcal{U} \subset \mathcal{G}$ and the set of observed function $\mathcal{F}$, a score function can be defined following Vazquez et al. [3] as

$$E[\{X_i\}_{i=1}^N] = -\sum_{ij} J_{ij} \delta(X_i; X_j) - \sum_i h_i(X_i) \tag{1}$$

where J_{ij} is the adjacency matrix of $\mathcal{U}$ ($J_{ij} = 1$ if i and $j \in V \backslash A$ and they interact with each other). $\delta(\sigma; \tau)$ is the Kronecker delta function measured between functions σ and τ assigned to the neighboring proteins and $h_i(\tau)$ is an external field that counts the number of classified neighbors of protein i in the original graph $\mathcal{G}$ that have at least function τ. The Gibbs potential can than be calculated as a variational way to compute the quantity

$$F = -\frac{1}{\beta N} \log \left(\sum_{\{X_i\}_{i=1,\ldots,N}} e^{-\beta E[\{X_i\}_{i=1,\ldots,N}]} \right) \tag{2}$$

called free-energy of the system. Configurations with a largest statistical weight can then be calculated as those maximizing this potential function. Using the message passing approach [1] under the assumption that correlations are low enough in the graph so that one can write $P_{ij}(X_i, X_j) \propto P_i(X_i) P_j(X_j)$ if proteins i and j are chosen at random, one can calculate each $P_j(X_j)$ as product of conditional probabilities contributions $M_{i \to j}(X_j)$ incoming

to j from all neighbors of protein j, conditional to the fact that j has function X_j:

$$P_j(X_j) \propto \prod_{i \in I(j)} M_{i \to j}(X_j) = e^{\beta \sum_{i \in I(j)} u_{i \to j}(X_j)} \tag{3}$$

where $I(j) \subset V \backslash A$ denotes the set of unclassified neighbors of j and $u_{i \to j}(\sigma)$ is a "message" that represents the field in direction $\sigma \in \mathcal{F}$ acting on protein j due to the presence of protein i when protein j has function σ. Equations for the message functions can be solved iteratively as fixed points of the system of equations

$$M_{i \to j}(\sigma) = \sum_{\tau=1}^{F} \left(\prod_{l \in I(i) \backslash j} M_{l \to i}(\tau) \right) e^{\beta J_{ij} \delta(\sigma;\tau) + \beta h_i(\tau)} \tag{4}$$

one for each link of $\mathcal{U}$, for both directions in the graph. Self consistent BP equations can be rewritten in terms of messages u's, and solved as explained in [2].

Results

We ran our algorithm solving at high enough but non-infinite β since we were interested in predicting functional assignments that could be biologically allowed although not strictly maximizing the score function. The function probabilities for each protein converge on a set of values organize themselves into hierarchies. The probability values are β-dependent, but not so the hierarchical structure, which is highly conserved if β is chosen above a certain critical transition threshold. The conserved functional ranking structure was exploited in the analysis of the results. Ranking was changed into a confidence value on the prediction in the following way: we computed the fraction of annotated genes that have at least either a first or a second neighbor they share at least one function with. This was taken as a raw estimate of the fidelity of the guilt-by-association procedure up to distance 2 on the interaction graph.

The procedure was repeated for all three GO categories. For each gene, the first and second best ranked functions were then assigned a confidence value equivalent to the fraction predicted by the above method on the distance 2 neighbors graph. The other ranked functions were given a decreasing confidence value, proportional to the above fraction divided by the number of overall predicted functions on that gene. Other more sophisticated interpretations of the ranking results are of course possible, but are not currently implemented.

Nothing can be said for the 453 genes in the test set that do not appear in the interaction graph.

Bibliography

[1] Yedidia, J.S., Freeman, W.T. and Weiss, Y. Understanding Belief Propagation and Its Generalizations, Exploring Artificial Intelligence in the New Millennium, ISBN 1558608117, Chap. 8, pp. 239-236, (2003).

[2] Leone, M. & Pagnani, A. Predicting protein functions with message passing algorithms, *Bioinformatics* 21: 239-247, (2005).

[3] Vazquez, A, Flammini, A., Maritan, A. & Vespignani, A Global protein function prediction in protein-protein interaction networks. *Nature Biotech.* **21**, 697-700 (2003).

Methods for the naïve Bayes MouseFunc I classifiers

Murat Taşan

November 16, 2006

1 The Naïve Bayes Model

The naïve Bayes model is one of the simplest classification models used in practice, which is a redeeming feature and makes it a useful "baseline" model by which other — more complex — models can be compared against. Let an example $\mathbf{x} = (x_1, x_2, \ldots, x_d)$ be a d-dimensional binary feature vector where $x_j \in \{-1, 1\}$ for all $j \in \{1, 2, \ldots, d\}$. The case $x_j = 1$ indicates that example $\mathbf{x}$ has a positive ("true") annotation for feature j. Similarly, $x_j = -1$ indicates that example $\mathbf{x}$ does *not* have a positive annotation for feature j (i.e. a "false" annotation). Let $y \in \{-1, 1\}$ be the response variable for a particular classification task (e.g. GO term annotation). If $y = 1$, then the annotation is true; if $y = -1$, then the annotation is false.

The naïve Bayes model assumes independence amongst the d features in $\mathbf{x}$, *conditioned on* y. Put formally,

$$P(\mathbf{x}|y) = \prod_{j=1}^{d} P(x_j|y). \tag{1}$$

The full naïve Bayes model then is *generative* as the parameters are chosen to maximize the likelihood of generating all of the data (the examples and their associated responses).

Here, the genes and their binary attributes are the examples, and the GO annotation is the response. Rather than computing the probability of a true GO annotation directly, it is instead easier to compute the log-likelihood of

a true GO annotation as

$$\begin{aligned}\ell = \log\left(\frac{P(y=1|\mathbf{x})}{P(y=-1|\mathbf{x})}\right) &= \log\left(\frac{P(\mathbf{x}|y=1)P(y=1)}{P(\mathbf{x}|y=-1)P(y=-1)}\right)\\ &= \log\left(\frac{P(y=1)}{P(y=-1)}\right) + \log\left(\frac{P(\mathbf{x}|y=1)}{P(\mathbf{x}|y=-1)}\right)\\ &= \log\left(\frac{P(y=1)}{P(y=-1)}\right) + \sum_{j=1}^{d}\log\left(\frac{P(x_j|y=1)}{P(x_j|y=-1)}\right).\end{aligned} \tag{2}$$

Letting there be m training examples and associated responses, the likelihoods in equation 2 can be estimated by enumerating the training data as in the following example. To compute $P(x_j = 1|y = 1)$, one simply takes the ratio of the number (over the m cases) of training examples with *both* a positive GO annotation and a positive j-th feature annotation to the number of training examples with a positive GO annotation. An analogous counting measure is made for each combination (4 such measures per feature: $P(x_j = 1|y = 1), P(x_j = -1|y = 1), P(x_j = 1|y = -1), P(x_j = -1|y = -1)$). A single pseudo-count is added to each of the four counts to prevent the computation of infinite values.

The log-likelihood ℓ of a positive GO annotation is converted back to a probability of annotation via

$$P(y = 1|\mathbf{x}) = \frac{e^\ell}{1 + e^\ell}, \tag{3}$$

although a loss in precision may result if the log-likelihood values are too extreme. The two values ($P(y = 1|\mathbf{x})$ and ℓ) are monotonically related however, thus confidence-rated classification can be based on ℓ alone.

2 Training Dataset Organization

All of the data for the naïve Bayes model was obtained directly from the shared Mousefunc dataset (i.e. that data available to all participants). Table 2 enumerates the datasets used by the model, and provides the short names that will be used throughout the remainder of this document. Any post-processing performed on each dataset will be described in the following subsections (each devoted to one dataset).

matrix name(s)	full name
pfam	Sanger Institute Pfam domain data
interpro	EBI InterPro domain data
phenotype	MGI phenotype ontology data
biomart	bioMart phylogenetic profile data
inparanoid	Inparanoid phylogenetic profile data
omim	OMIM (via homology) disease data

Table 1: Datasets (full names and short names used throughout the methods description) employed by the random forests classifiers.

The training data is provided for all of the 21603 *Mus musculus* genes, although for many of the genes particular datasets in the training data are missing.

2.1 Protein domain data

Protein domain data was provided only in binary form via the files `interpro_domainData.txt` and `pfamA_domainData.txt`. The data in these files was mapped directly to the *interpro* and *pfam* matrices, with 5405 and 3133 columns, respectively. Each column corresponds to a distinct InterPro or Pfam protein domain, which are then viewed as features in the naïve Bayes model.

2.2 Phenotype data

Phenotype data was processed in binary form via the file `MPheno_OBO.ontology`. The organizational structure of the phenotype ontology (i.e. the definitions and structure found in the `MPheno_OBO.ontology` file) is ignored. The *phenotype* matrix then is a direct mapping of the data, and consists of 33 columns corresponding to each distinct "leaf" phenotype characteristic, which is then viewed as a feature in the naïve Bayes model.

2.3 Phylogenetic profile data

The phylogenetic data is provided in both binary are real-valued forms, from two data sources: bioMart and Inparanoid. For both data sources, the real-valued data is ignored and only the data in the `phylogenetic_binary.txt`

and inparanoid_binary.txt files are used. Each file maps directly to the *biomart* and *inparanoid* binary matrices with 18 and 24 columns, respectively; each viewed as a feature in the naïve Bayes model.

2.4 Disease data

The OMIM disease data is provided only in binary form, and no further post-processing is performed on it. The *omim* matrix then consists of 2488 columns, corresponding to each distinct disease identified in the omim_disease.txt file; each also being viewed as a feature in the naïve Bayes model.

3 Target Dataset Organization

Provided in the MousefuncI datasets is the complete GO annotation record for 19885 of the 21603 *Mus musculus* genes. 5416 distinct GO terms are identified as having one of these genes annotated to them, although only 2815 of these terms are considered as targets for MousefuncI prediction. These target GO terms are broken up into twelve categories by a combination of branch and annotation count. The three branches are biological process (BP), molecular function (MF), and cellular component (CC); the annotation count groupings across each branch are $[3, 10]$, $[11-30]$, $[31-100]$, $[101-300]$.

It should be noted here that of the 19885 training examples, only 10542 have some GO annotation (not including the terms "molecular function unknown", "biological process unknown", and "cellular component unknown"). This subset plays an important role in the training of the models (described below).

3.1 Training Set Pruning (Example Selection)

The MousefuncI competition contains two distinct parts. The first task is to predict the target GO term annotations for the held-out set of $21603 - 19885 = 1718$ genes. This is the testing analogue to assessing the performance of a classifier in predicting the annotations of a gene for which one has experimental data (i.e. the attributes used as columns in the matrices identified in table 2), but no GO annotation yet. The 1718 held-out test genes are selected uniformly from the mouse genes for which experimental data is available. For constructing the models used to make predictions for

the target GO terms in this portion of the competition, all 19885 training genes are used as examples.

The second task of the MousefuncI competition is to *update* the set of target GO annotations for the 19885 training genes. Since the GO annotation data (the target data) corresponds to an early-2006 snapshot of the GO, and the competition results are submitted in late-2006, one expects some of the target GO terms to have additional annotations (amongst the 19885 training genes). To train models used to make predictions for this portion of the competition, only the 10542 genes having some non-unknown GO annotation are used as training examples. Note that *any* non-unknown GO annotation qualifies for membership in this training example set, not just those genes with a *target* GO annotation. This choice reflects the notion that the competition will only consider for performance measurement those genes that have had some update, thus practically guaranteeing at least one GO annotation. The selection of this training set then is logical as one would like to train on data that is as closely representative (distributed identically) to the eventual test data.

4 Naïve Bayes Model with Feature Selection

The first model constructed used all features described in table 2 (i.e. no feature selection). Cross-validation performance showed that the model's ability to discriminate between positive and negative annotations was fairly weak, especially for GO terms with low cardinality. Essentially, much of the discriminative power is being "drowned out in the noise" of the thousands of non-useful predictor features. To combat this, a feature selection method was adopted. Before training each model, the d features are sorted into order by mutual information with the response. Consider a feature vector $\mathbf{f} \in \{-1, +1\}^m$ representing the m values for the feature amongst the training data. Similarly, consider the response vector $\mathbf{y} \in \{-1, +1\}^m$ representing the m responses (where $\mathbf{f}$ and $\mathbf{y}$ are ordered together, corresponding to the training examples). These two vectors then represent distributions over two random variables F and Y (both of which only taking values -1 or $+1$). Then the mutual information between random variables F and Y ($I(F;Y)$) is defined as:

$$I(F;Y) = \sum_{f \in F} \sum_{y \in Y} P(F = f, Y = y) \log \frac{P(F = f, Y = y)}{P(F = f)P(Y = y)}. \qquad (4)$$

The probabilities in equation 4 are estimated in the same fashion (i.e. counting) as that described above for the naïve Bayes model.

With each feature's mutual information with the response, the features can now be added to the model in order of decreasing "importance". A series of thresholds were tested, with the final adopted number of features being 64 for all categories. That is, the 64 top-scoring (via mutual information with the response) features are incorporated into the naïve Bayes model. This has the effect of setting $d = 64$ in equation 2.

5 Final Predictions

5.1 Test Set Predictions

Predictions for the held-out test set of genes were made by training (via the counting procedure) the two naïve Bayes models (full-featured, and the 64-feature model) on all provided training examples.

5.2 Novel Predictions

Predictions for new annotations amongst the training examples were carried out by training the two naïve Bayes models only on those 10542 training examples with some non-unknown GO annotation.
